# *Mechanometallaphotoredox* Catalysis: Utilizing Increased Throughput Mechanochemistry to Develop Solvent-Minimized Aryl Amination and C(sp2)-C(sp3) Cross-Coupling Reactions with Increased Tolerance to Aerobic Conditions

**DOI:** 10.1021/jacs.5c05503

**Published:** 2025-05-22

**Authors:** Francis Millward, Eli Zysman-Colman

**Affiliations:** Organic Semiconductor Centre, EaStCHEM School of Chemistry, 7486 University of St Andrews, St Andrews KY16 9ST, U.K.

## Abstract

Photocatalysis as a tool used in organic synthesis has predominantly relied on the use of solvents, be it under homogeneous or heterogeneous conditions. In particular, metallaphotoredox catalysis reactions commonly use toxic organic solvents such as DMA and DMF. Herein, we demonstrate how mechanophotocatalysis, the synergistic union of mechanochemistry and photocatalysis, is compatible with this class of dual catalysis reactions involving both photocatalyst and nickel­(II) cocatalysts. Using ball milling, these mechanistically complex reactions can be conducted in the absence of a bulk solvent and under air, affording high-yielding aryl aminations and C­(sp2)-C­(sp3) cross-couplings with alkyl carboxylic acids, alkyl trifluoroborate salts, and alkyl bromides. These advances are facilitated by the introduction of a novel reaction vessel design for conducting four mechanophotocatalysis reactions simultaneously. This work highlights the promise of solvent-minimized photocatalysis reactions, demonstrating that in these examples bulk solvent is redundant, thus significantly reducing this waste stream. Through time-resolved photoluminescence studies, we observed that the excited states of five different photocatalysts were quenched by oxygen more significantly in solution than in the solid state, providing evidence for the origin of the increased tolerance to aerobic conditions that these mechanophotocatalysis reactions experience.

## Graphical abstract

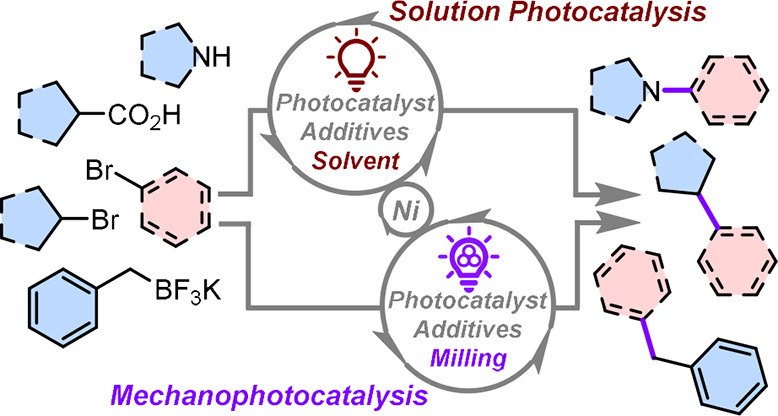

## Introduction

Solution-state photocatalysis is an established and widely used synthetic methodology, employing visible light to drive energy and electron transfer reactions.^1–6^ In particular, metallaphotoredox catalysis, involving the synergistic dual use of a transition metal catalyst and a photocatalyst, has dramatically expanded the utility of light-driven transformations, and is one of the most valuable applications of photocatalysis toward the synthesis of industrially relevant small molecules.^7^ While copper and palladium complex cocatalysts have seen some use in metallaphotoredox reactions,^8,9^ nickel complexes remain the cornerstone of these approaches, enabling a diverse range of new cross-coupling strategies.^10,11^ The key value of this approach stems from its complementary reactivity to traditional palladium-catalyzed cross-coupling reactions: palladium catalysis excels in the construction of C­(sp2)-C­(sp2) bonds, while photocatalyzed/nickel-mediated C­(sp3)-C­(sp2) and C­(sp3)-C­(sp3) cross-couplings allow access to saturated substructures using a wider diversity of coupling partners.^3,12^ Within these reactions, typically the nickel catalyst undergoes oxidative insertion into an aryl or alkyl halide bond, traps radicals generated by the photocatalyst, and ultimately undergoes reductive elimination to afford the cross-couped product, while the photocatalyst is primarily responsible for generating radical species and for turning over the nickel cycle by modulating the oxidation state of the nickel catalyst via photoinduced electron transfer (PET), or by sensitizing the nickel catalyst via photoinduced energy transfer (PEnT), Figure 1a. A wide range of oxidizable carbon-centered radical precursors are available; including carboxylic acids (following deprotonation),^3,13–19^ trifluoroborate salts,^20,21^ Hantzsch ester derivatives,^22^ α-amino carbon centers,^13,23^ and alcohols (primed as oxalates or NHC derivatives).^24–28^ Alkyl halides^12,29–34^ and alkanes^35,36^ can also be employed as carbon-centered radical precursors using an additional halogen atom transfer (XAT) or hydrogen atom transfer (HAT) agent, in selective reactions governed by the relative bond dissociation energies of the abstractant and substrate. Furthermore, boronic acids^37^ and boronic esters^38^ can function as alkyl radical precursors, and amines can serve as radical precursors as Katritzky salts^39^ or imines.^40^ Metallaphotoredox can also mediate carbon-heteroatom bond formations; including amination,^41–45^ etherification,^42,46^ thioetherification,^47,48^ esterification,^49,50^ and sulfonylation^51^ strategies, among others. The use of ancillary ligands (frequently 2,2′-bipyridine derivatives) or additives such as 1,4-diazabicyclo[2.2.2]­octane (DABCO), 1,1,3,3-tetramethylguanidine (TMG) or *tert*-butylamine, are often required to promote reactions.^41,52,53^ Yet despite the versatility of solution-state metallaphotoredox catalysis, this approach has almost exclusively been applied under strictly anaerobic, homogeneous catalysis conditions, requiring the use of toxic, unsustainable, and particularly flammable organic solvents such as *N,N*-dimethylacetamide (DMA), *N,N*-dimethylformamide (DMF), acetonitrile (MeCN), dioxane, or dimethyl sulfoxide (DMSO).^54–56^ The use of these at scale presents a combination of safety and sustainability concerns, ultimately limiting the impact and utility of metallaphotoredox catalysis in industry. Reaction screening for generating compound libraries is also hampered by the need to rigorously exclude oxygen from the system, and by the challenging removal of high boiling point solvents like DMA and DMSO.

**Figure 1:**
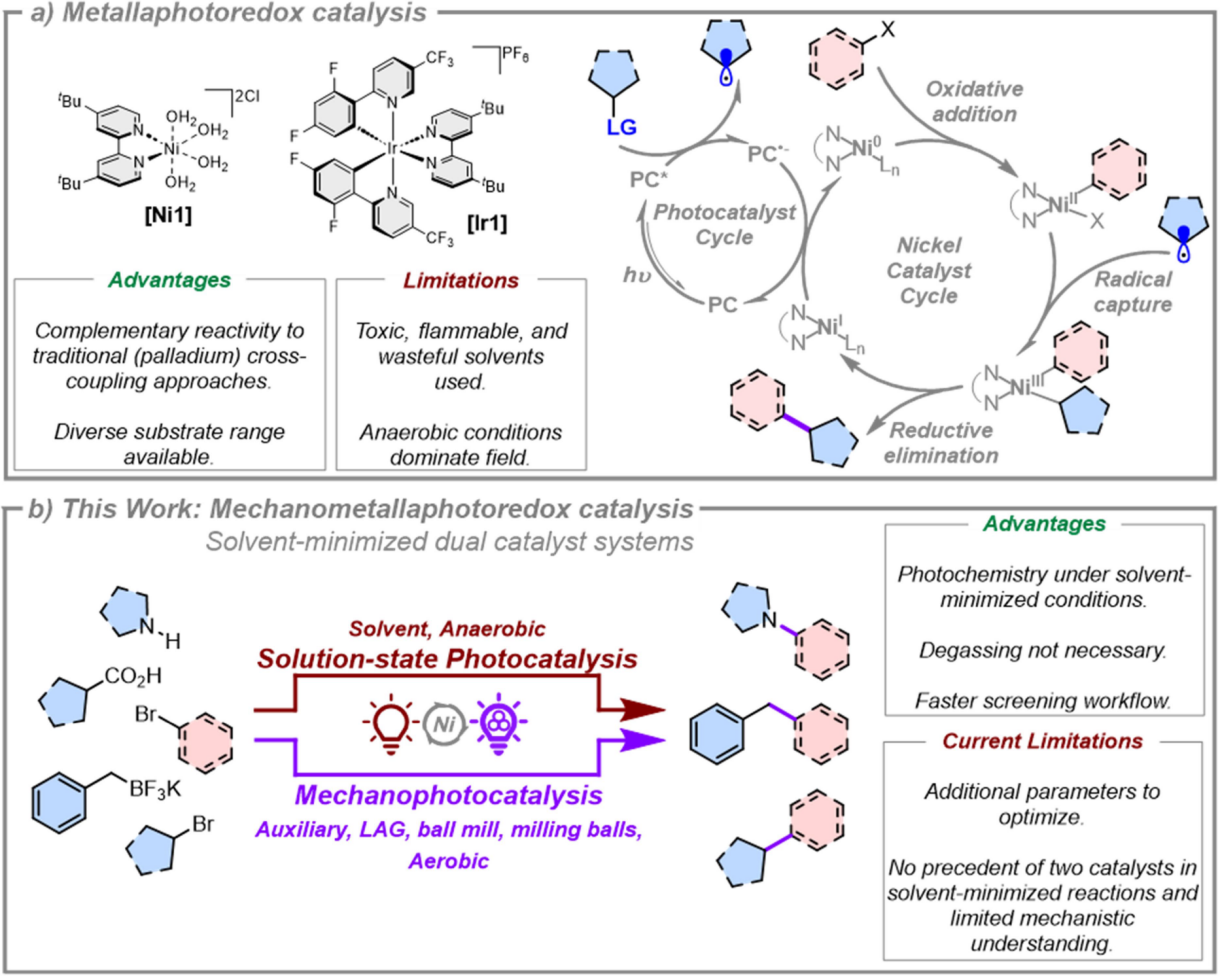
(a) Commonly presented putative mechanism for nickel/photoredox dual catalyst systems.^3^ Many variants of this mechanism are possible, however, due to the large number of photoactive species present that can initiate reactions with different substrates.^10,18,82,83^ It is reasonable to hypothesize that multiple mechanisms may be operational simultaneously, and the dominant mechanism is likely to be substrate and reaction specific, and dependent on the relative rates and the nature and concentration of the species present.^10,82^ (b) This work: utilizing mechanochemistry to develop solvent-minimized metallaphotoredox catalysis reactions.

Separately, mechanochemistry has rapidly evolved to become a robust and time-efficient tool for conducting a wide range of ground-state thermally controlled reactions. While many solution-state reactions require an inert atmosphere to function, mechanochemical reactions usually do not. Thus, mechanochemistry reactions can often be simpler to set up, faster, and produce much less solvent waste.^57–63^ However, while mechanochemistry is readily amenable to two-electron ground-state chemistry, single-electron transfer mechanochemistry is more difficult to achieve without using reactive zerovalent metals,^64–68^ or via mechanoredox chemistry.^69–73^ For instance, Ito and co-workers recently reported a metallaphotoredox-like aryl amination reaction using BaTiO_3_;^71^ however, due to the relatively small redox windows of current piezoelectric materials, and without the capacity to access PEnT reactions, this approach is potentially limited in terms of the range of substrates, particularly when compared to the versatility offered by photocatalytic methodologies. In efforts to address this limitation, several groups have attempted to combine mechanochemistry and photochemistry within a single reactor.^74–81^ Our group has previously disclosed the development of four archetypal photocatalysed reactions using ball milling;^74^ however, these reported examples are of modest value to industry, and reaction throughput is limited by the current equipment used in the field. Here, we report the development of *mechanometallaphotoredox catalysis* reactions, harnessing the synergistic reactivity of photocatalysts and nickel cocatalysts to conduct aryl aminations and C­(sp^2^)-C­(sp^3^) cross-couplings, which are of particular interest to industry, under solvent-minimized, aerobic conditions, Figure 1b. A new open-source reaction vessel design allows for increased screening throughput, and mechanistic investigations are conducted to identify the origin of the improved tolerance to air that is a feature of these mechanophotocatalysis reactions.^74^

## Results and Discussion

### Increased Throughput Mechanophotocatalysis

In our previous report, we were limited to conducting two mechanophotocatalysis reactions per ball mill. In an effort to accelerate reaction screening and simplify the vessel design, we have developed new reaction vessels and holders designed to increase the throughput of mechanophotocatalysis reactions, Figure 2.^74^ We used transparent polypropylene Eppendorf vials as chemically resistant, cheap, single-use reaction vessels. As an initial proof-of-concept, and in line with our previous report, we encapsulated the Eppendorfs inside poly­(methyl methacrylate) (PMMA) jars to allow for safe, secure clamping in the mill, which we refer to as the ‘V1 holder’. This vessel design was used for much of the optimization of the reactions presented in this study. Subsequently, we developed an improved ‘V2 holder’, that permits four Eppendorf reaction vessels to be clamped per mill, doubling the throughput compared to our first-generation mechanophotoreactor setup.

**Figure 2:**
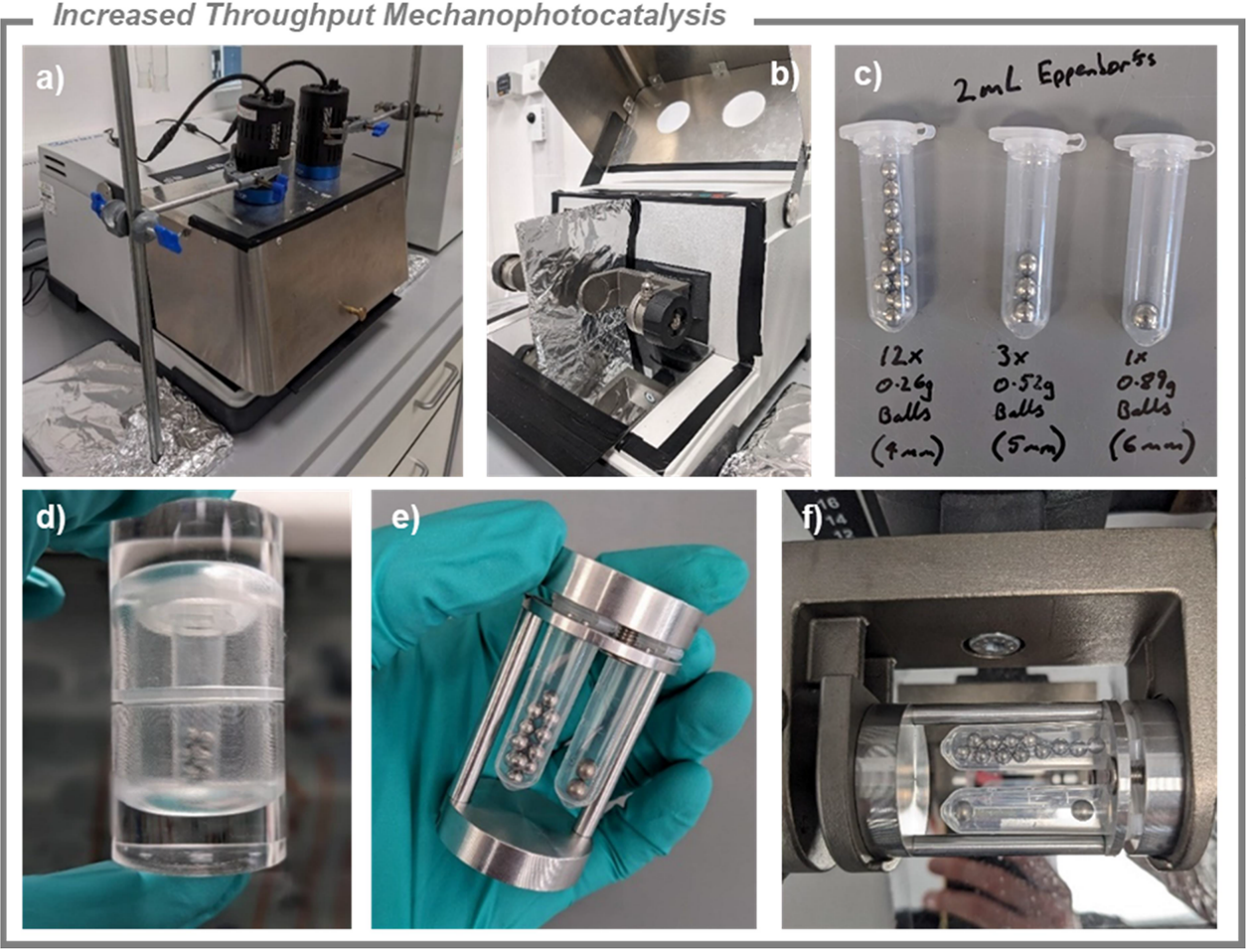
Mechanophotocatalysis reactor and dual throughput holder. (a) Retsch MM400 mill retrofitted with a stainless-steel cover enabling light irradiation of each jar holder position with *Kessil* LEDs. (b) The interior of the reactor. (c) Eppendorfs used as single-use reaction vessels, containing 4 mm (0.26 g each), 5 mm (0.52 g each), and 6 mm (0.89 g) diameter milling balls. (d) ‘V1 holder’: PMMA milling jar used to encapsulate the reaction vessel and allow clamping in the mill. (e) ‘V2 holder’ with Eppendorf reaction vials. (f) ‘V2 holder’ clamped in the mechanophotocatalysis reactor. Design schematics for the ‘V2 holder’ are available in the SI.

### Aryl Amination

Metallaphotoredox catalysis is a capable tool for constructing C­(sp2)-N bonds,^41–44,83,84^ complementing thermally controlled SNAr, Buchwald Hartwig, Ullmann, and Cham-Lam couplings. Typical conditions include the use of nickel salts such as nickel­(II) bromide glyme (NiBr_2_•DME), and DABCO as a multifunctional additive: a stabilizing monodentate ligand, an electron donor, hydrogen atom transfer (HAT) agent, and as a base.^41^ We began by exploring the amination of methyl 4-bromobenzoate (**1a**) with mono-Boc-protected piperazine (**2a**), using NiBr_2_•DME as the nickel catalyst and [Ir­(dF­(CF_3_)­ppy)_2_(d*t*bbpy)]­PF_6_ (**[Ir1]**) as the photocatalyst.^41^ In accordance with our previously reported conditions, all mechanophotocatalysis reactions were conducted under air.^74^ As the solution-state reaction typically uses DMA as a solvent, we decided to employ this as a liquid assisted grinding (LAG) agent.^85^ Solvent-minimized photocatalysis reactions that we have explored in the past typically manifest as ‘*sticky-to-slushy*’ organic pastes. Thus, in order to control the rheology of the mixture and facilitate efficient mixing, we selected sodium sulfate as an inert grinding auxiliary (Aux.). We found that higher auxiliary loadings were beneficial, with 15 or 18 equiv of sodium sulfate giving superior NMR yields (71 and 70%) than obtained using only 12 equiv (52%), Figure 3a. DMSO, another solvent used in the solution-state literature,^41^ as a LAG agent gave an inferior yield (39%). Decreasing the LAG loading from 6 to 1.5 equiv did not result in a reduction in the yield (obtaining 67, 70, 69, and 68% for 6, 4.5, 3, and 1.5 equiv of DMA, respectively); however, removal of the LAG agent decreased the yield significantly (24%), potentially the result of either less efficient mixing of reagents or because DMA is noninnocent in the reaction mechanism. Indeed, in Ito’s mechanoredox nickel-catalyzed amination and Browne’s manganese- and zinc-driven nickel-catalyzed reactions in ball mills, DMA at comparable loadings was a beneficial additive.^64,65,71^ Control experiments involving the removal of the photocatalyst, nickel cocatalyst, or light resulted in no reaction, demonstrating that a light-driven dual catalyst system is functioning under a solvent-minimized environment. Interestingly, removal of the milling balls while maintaining the milling speed still afforded a high yield (67%), meaning that the shaking action of the mill alone was sufficient to mix the reagents, which is highly promising for the development of lower energy milling technologies to enable the future scale-up of this technology. Using the auxiliary and LAG loadings optimized for the 0.1 mmol scale reaction, we observed a noticeable increase in yield when changing to a 0.3 mmol scale using larger milling balls (76% with three 0.52 g balls), perhaps as a function of an improved milling environment with a larger portion of the reaction vessel full and larger milling balls facilitating improved mixing. This highlights the complicated relationship between the yield of a mechanochemical reaction and the myriad reaction parameters that need to be congruently optimized. Further scale-up in the current reaction vessels is limited by the internal volume of the vials. Removal of the milling balls from the 0.3 mmol scale reaction while maintaining milling at 25 Hz resulted in a decreased yield (58%), demonstrating that the increase in scale demands more efficient mixing. Finally, we tested the reaction using our V2 holder with a larger (0.89 g) milling ball, which afforded a comparable NMR yield of 76 ± 1%. Having identified optimal conditions for this aerobic mechanometallaphotoredox reaction, we proceeded to benchmark it against the corresponding solution-state reaction. Under both aerobic and anaerobic conditions, the solution-state reaction is complete within the same 3-h time window used in the mechanophotocatalysis experiment (80 ± 1 and 83 ± 4%, respectively), Figure 3a. The mechanophotocatalysis reaction provides a competitive yield (76 ± 1%) while mediating a > 28-fold reduction in the consumption and waste of the reaction solvent.

**Figure 3:**
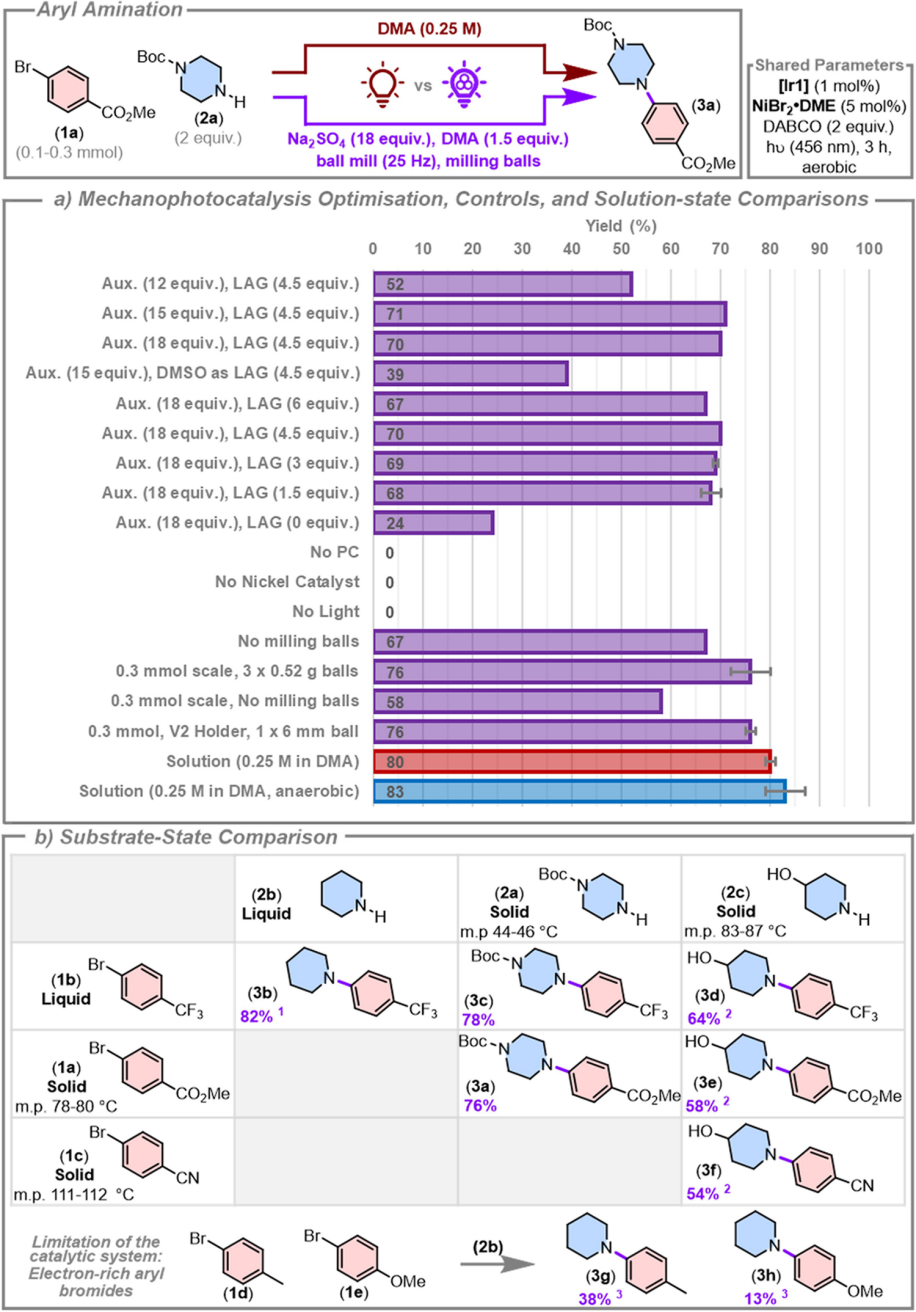
Aryl aminations enabled by mechanometallaphotoredox catalysis. Yields are from quantitative ^1^H NMR spectroscopy experiments using 1,3,5-trimethoxybenzene as an internal standard. Key experiments, marked with error bars, are the average result of two experiments. (a) Mechanophotocatalysis reaction optimization and control reactions, and solution comparisons. Unless stated otherwise, mechanophotocatalysis reactions used the V1 holder on a 0.1 mmol scale, with auxiliary (18 equiv), LAG (1.5 equiv), and milling balls (12 × 0.26 g). Solution reactions were conducted at a 0.3 mmol scale at 0.25 M concentration in DMA. (b) Substrate-state comparison. Conducted at 0.3 mmol scale using the V2 holder with 1 × 0.89 g ball for 3 h under aerobic conditions. ^1^0.3 mol % photocatalyst. ^2^0.1 mol % photocatalyst. ^3^Modified conditions, see SI for details.

As the rheology of the mixture in a mechanochemical environment significantly impacts the mixing efficiency of the reagents, we assessed whether the use of liquid substrates or higher melting point solids, in place of the low melting point solids **1a** and **2a**, would impede the efficiency of the solvent-minimized reaction, Figure 3b. Under the previously optimized LAG and auxiliary loading conditions, all reactions proceeded regardless of the physical state and melting points of the starting materials. For instance, the reaction of liquid 4-bromobenzotrifluoride (**1b**) afforded arylated amine products with the liquid piperidine (**2b**), the lower melting point **2a**, and the higher melting 4-hydroxypiperidine (**2c**) in high yields (82, 78, and 64%, respectively). Compound **2c** was also coupled with lower melting point **1a** and higher melting point solid 4-bromobenzonitrile (**1c**) in good yields (58 and 54%, respectively). Nickel-mediated aryl aminations generally perform poorly with electron-rich aryl bromides without significant modifications to the catalyst system.^42,45,86,87^ Unfortunately, this issue is not solvable via the removal of the bulk solvent from the reaction system, and despite attempts to optimize mechanophotoredox conditions for the coupling of 4-bromotoluene (**1d**) and 4-bromoanisole (**1e**) with **2b**, only poor yields of 38 and 13%, respectively, were obtained (see SI for details and further discussion).

### C­(sp2)-C­(sp3) Decarboxylative and Deborylative Cross-Coupling

Having demonstrated an efficient proof-of-principle solvent-minimized metallaphotoredox catalysis reaction, we turned our attention to a reaction where an ancillary ligand is required for the nickel catalyst. Oxidatively generated radical precursors like carboxylic acids and potassium alkyl trifluoroborate salts were among the first substrates to be used in metallaphotoredox reactions.^13,21^ These bench-stable and readily synthetically accessible or commercially available substrates can be oxidized by a photocatalyst (*E*_ox_(Boc-proline carboxylate) = 0.95 V vs SCE in MeCN, *E*_ox_(potassium benzyltrifluoroborate) = 1.05 V vs SCE in MeCN),^15,20,88^ leaving the photocatalyst free to reduce the nickel catalyst to turn over both cycles. We have previously shown that photocatalytic decarboxylative alkylations in a solvent-minimized environment are feasible, and thus we anticipated that a decarboxylative arylation under a metallamechanophotoredox framework would also be viable.^74^ We began our investigations using the decarboxylative arylation of Boc-protected proline (**2c**) with **1a** using **[Ir1]** as the photocatalyst, Figure 4a. For ease of use, we opted to use the preformed [Ni­(d*t*bbpy)­(OH_2_)_4_]­Cl_2_ (**[Ni1]**) as a bench-stable preformed nickel catalyst.^22,51^ Initially, we obtained a poor yield (37%) using low auxiliary and LAG loadings (3 and 1.5 equiv of sodium sulfate and DMA, respectively). Progressive improvements in yield were made using 6 equiv of auxiliary (55%) and subsequently 3 equiv of LAG (64%). A higher LAG loading gave negligible improvements (66% with 4.5 equiv DMA), as did the use of 9 or 12 equiv of auxiliary (66 and 68%, respectively), suggesting that a plateau was reached as a function of the optimization of both parameters. Notably, the nickel catalyst loading could be reduced to 1 mol % while maintaining high yields (68%); a remarkable outcome considering the low loadings of each catalyst used in the absence of bulk solvent. Removal of the nickel catalyst resulted in a nonzero yield (9%), suggesting the presence of a small purely photocatalytic background reaction, while controls without the photocatalyst, light, or LAG agent resulted in no formation of the target product. It is possible the LAG agent may play a dual role in stabilizing transient nickel species as a ligand while also facilitating more efficient mixing of the reagents. Removal of the milling balls led to a more significant yield reduction (45%), showing that the different rheology of this reaction mixture relative to the arylation described above necessitates an increased mechanochemical input for efficient reactivity. It was not necessary to use the preformed nickel cocatalyst, as use of NiCl_2_•DME and 4,4′-di-*tert*-butyl-2,2′-bipyridine (d*t*bbpy) resulted in a yield of 62%, indicating that the active nickel cocatalyst can be formed in situ in the absence of bulk solvent. Finally, a yield of 77 ± 0% was obtained utilizing the V2 holder and a single larger milling ball. We then cross-compared the efficiency of these optimized conditions to the solution-state reaction, Figure 4a. At a literature-reported concentration of 0.02 M,^13,89^ and under air, the reaction is complete within the same 2 h window as the mechanophotocatalysis reaction (82 ± 1%). Once again, the solvent-minimized version provides a competitive yield (77 ± 0%), with an approximately 180-fold reduction in reaction solvent use. It should be noted that the dilute concentration used in this reaction^13,89^ (>500 equiv of DMA with respect to the limiting reagent) can be modified to a more concentrated version at 0.1 M for this system without significantly impacting the yield over the 2 h window (81 ± 2%); however, even under these conditions the mechanophotocatalysis reaction still proceeds with an approximately 36-fold reduction in the usage and waste of the reaction solvent.

**Figure 4:**
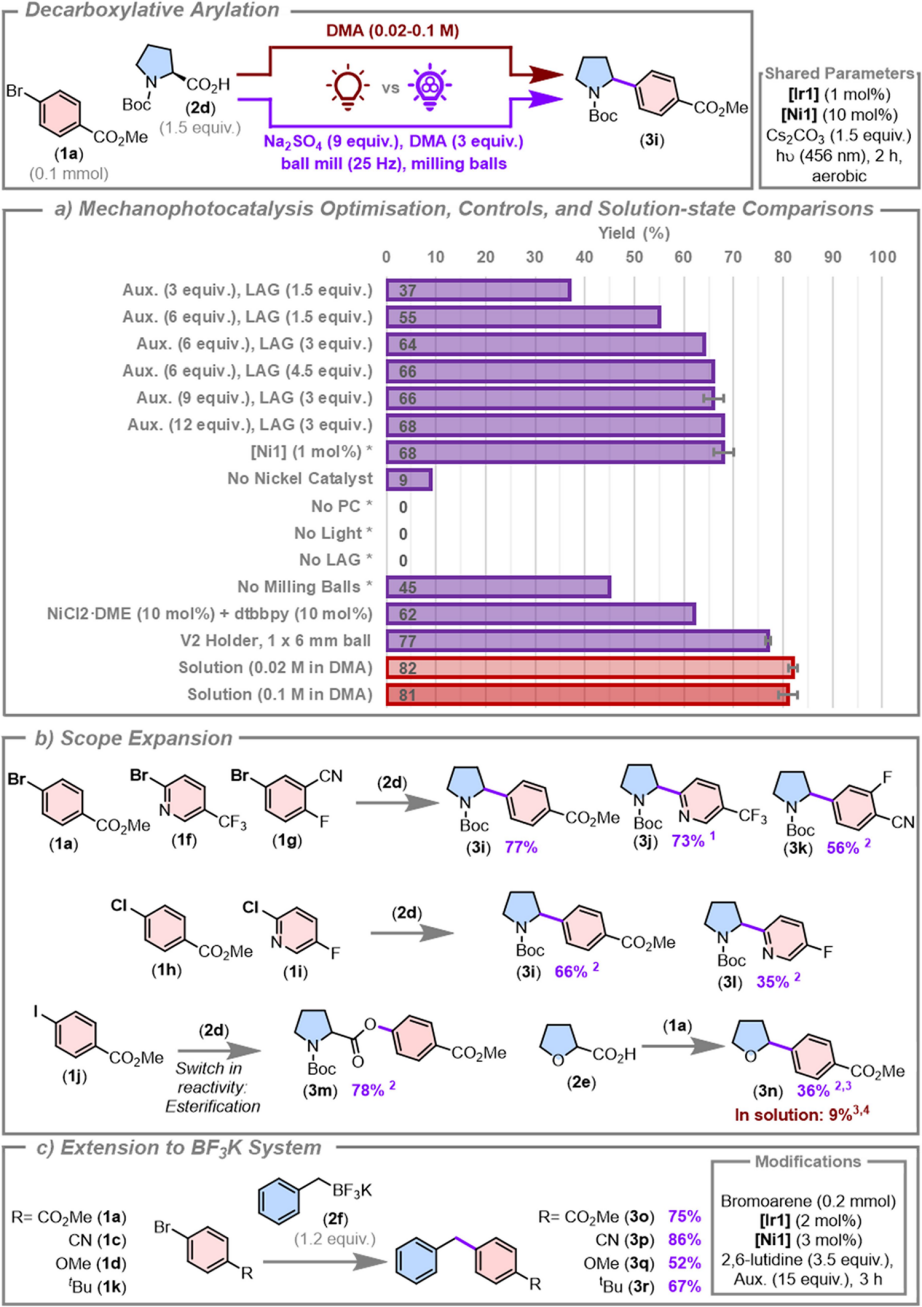
C­(sp2)-C­(sp3) decarboxylative and deborylative cross-couplings via mechanometallaphotoredox catalysis. Yields are from quantitative ^1^H NMR spectroscopic experiments using 1,3,5-trimethoxybenzene as an internal standard. Key experiments, marked with error bars, are the average result of two experiments. (a) Mechanophotocatalysis reaction optimization and control reactions, and solution comparisons. Unless stated otherwise, mechanophotocatalysis reactions used the V1 holder on a 0.1 mmol scale, with auxiliary (9 equiv), LAG (3 equiv), milling balls (12 × 0.26 g). * **[Ni1]** (1 mol %). Solution-state reactions were conducted under aerobic conditions in DMA. (b) Reaction scope study, highlighting the use of different aryl halides, and the modifications required to activate a different carboxylic acid substrate. ^1^Aux. (15 equiv), 3 h. ^2^Aux. (15 equiv), LAG (6 equiv), 1 × 0.71 g ball, 4.5 h. ^3^Carboxylic acid (3 equiv), potassium phthalimide as the base (3 equiv), **[Ir1]** (2 mol %).^4^ Solution-state comparison: DMA (0.1 M), in air, 4.5 h. (c) The use of potassium benzyltrifluoroborate as the radical precursor substrate.

We next proceeded to explore a substrate scope of different aryl halides for this reaction, Figure 4b. In general, the original lower auxiliary and LAG loadings (9 and 3 equiv, respectively) used for the optimization afforded lower yields, and increasing the auxiliary loading to 15 equiv and LAG loading to 6 equiv, along with increased reaction times, were required to increase the reaction yield. This observation indicates that rheology parameters such as these need to be tailored to individual substrate pairs in order to optimize the reaction yield. Various aryl bromides containing methyl ester (**3i**), trifluoromethylpyridine (**3j**), and fluoro and cyano groups (**3k**) were coupled to **2d** in generally high yields (77, 73, and 56%, respectively). Aryl chlorides **1h** and **1i** were also compatible, affording yields of 66 and 35% at a reaction time of 4.5 h. However, the use of methyl 4-iodobenzoate (**1j**) resulted in an unexpected esterification, giving the product **3m** in a high yield of 78%. The solution-state reaction comparison under aerobic conditions in the same time gave the decarboxylated and esterified products in 15 and 10% yield, respectively, with 59% recovery of the starting material. The origin of this switch in reactivity is unclear, and further studies are underway to investigate this. Finally, we explored the use of tetrahydrofuran-2-carboxylic acid (**2e**) as a substrate. The use of cesium carbonate gave a solid reaction mixture that resulted in poor mixing; thus, the base was changed to potassium phthalimide. Not only was mixing more facile, but this also generates phthalimide in situ, which is a stabilizing additive in decarboxylative arylation type reactions,^52^ with these changes giving a yield of 36%. In the same reaction time, the aerobic solution-state analogue of this reaction gave only 9% product yield.

Finally, we explored whether potassium benzyl trifluoroborate (**2d**) could be used as the radical precursor under solvent-minimized conditions, Figure 4c. Using slightly modified reaction conditions (including increasing the auxiliary loading to account for using 2,6-lutidine as a liquid base) electron-withdrawing aryl bromides were tolerated well in the coupling with potassium trifluoroborate (75 and 86% yield for the methyl ester (**1a**) and nitrile (**1c**) substrates, respectively). Substrates with electron-donating methoxy (**1d**) and *tert*-butyl (**1e**) groups were also tolerated, though at slightly decreased yields (67 and 52%, respectively), with the lower yield of the latter reaction likely reflecting a slower reaction (39% of **1d** remained after 3 h).

### XAT-Enabled Cross-Electrophile Coupling

We wanted to push this approach further by demonstrating that complex reaction mechanisms involving the sequential generation of multiple radical species are still compatible with a solvent-minimized methodology. To this end, we selected the cross-electrophile coupling of aryl and alkyl bromides mediated by a XAT agent, such as tris­(trimethylsilyl)­silane (TTMSS).^12^ Our model reaction system included **1a** and Boc-protected 4-bromopiperidine (**2g**) as the coupling partners, sodium carbonate as a base, **[Ir1]** as the photocatalyst, and **[Ni1]** as the nickel cocatalyst, Figure 5a. Using 2 equiv of 1,2-dimethoxyethane (DME) as a LAG agent and a 2-h reaction time, we found that auxiliary loadings of 12, 15, or 18 equiv of sodium sulfate afforded similar yields (56–61%), while the use of 21 equiv of auxiliary led to a moderate decrease in yield (44%). The removal of the LAG agent also led to a significant reduction in yield (31%), while increasing the LAG loading from 2 to 3 and 4 equiv gave comparable yields (61–64%). Control reactions in the absence of photocatalyst, nickel cocatalyst, or light produced no product. Removal of the milling balls gave a much lower yield of 35%, suggesting the greater importance of more efficient mixing in this more complex system, and the yield could be increased to 68 ± 1% in 3 h using the V2 holder at a 0.3 mmol scale with one larger ball (0.89 g). We then compared the efficiency of the mechanophotocatalysis protocol with the solution-state reaction conducted at 0.1 M concentration in DME under both aerobic and anaerobic conditions. While the anaerobic solution-state reaction gave the product in 77 ± 0% yield in 3 h, the yield of the solution-state reaction under air was lower (51 ± 4%); further, the yield did not improve over a longer reaction time (47 ± 2% in 24 h). Thus, under air, the solution-state reaction was less efficient than the mechanophotocatalysis reaction (68 ± 1%). This reduction in yield is a result of the aerobic solution-state reaction conditions producing a significant protodehalogenated byproduct (41 ± 4%), while both the mechanophotocatalysis and anaerobic solution-state reactions produced significantly smaller amounts of this byproduct (20 ± 3 and 22 ± 0%, respectively). We tentatively assign this to be a function of more efficient quenching of the triplet excited state of the photocatalyst by oxygen in solution, encouraging protodehalogenation following oxidative insertion of the nickel catalyst to the aryl bromide. The improved efficiency of the mechanophotocatalysis experiment relative to the solution-state reaction under aerobic conditions is consistent with the results from our previous study.^74^

**Figure 5:**
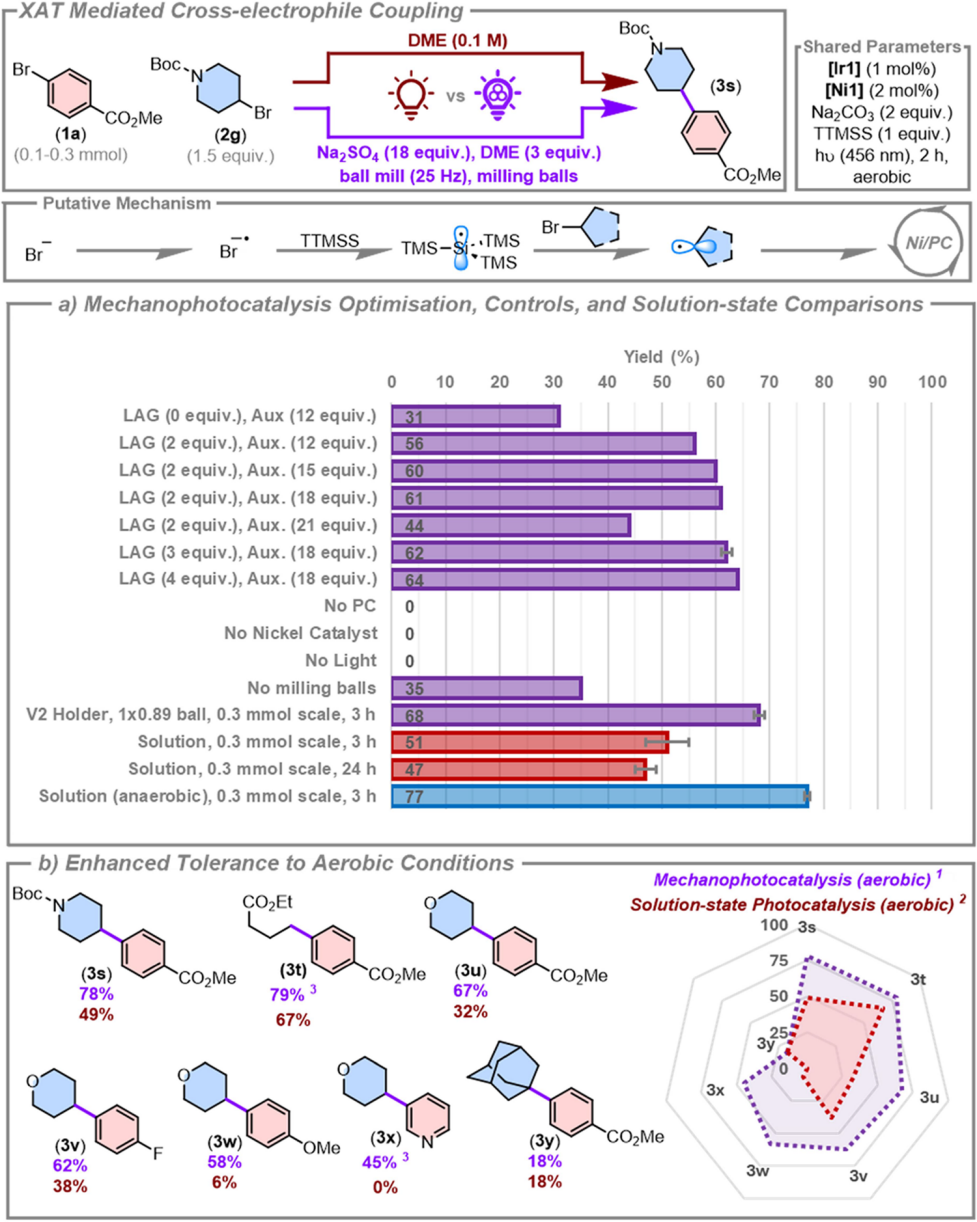
Cross-electrophile coupling via XAT using mechanometallaphotoredox catalysis. Yields are from quantitative ^1^H NMR spectroscopy experiments using 1,3,5-trimethoxybenzene as an internal standard. Key experiments, marked with error bars, are the average of two experiments. (a) Mechanophotocatalysis reaction optimization and control reactions, and solution-state photocatalysis comparison. Unless stated otherwise, mechanophotocatalysis reactions used the V1 holder on a 0.1 mmol scale for 2 h, with **[Ir1]** (1 mol %), **[Ni1]** (2 mol %), auxiliary (18 equiv), LAG (3 equiv), milling balls (12 × 0.26 g). Solution-state photocatalysis reactions were conducted at 0.1 M concentration in DME. (b) Substrate scope with mechanophotocatalysis and solution-state photocatalysis under aerobic conditions. ^1^Optimal mechanophotocatalysis conditions: 0.3 mmol scale, **[Ir1]** (2 mol %), **[Ni1]** (1 mol %), 1 × 0.89 g ball, 3 h. ^2^Solution-state comparison of optimal mechanophotocatalysis conditions, with DME (0.1 M). ^3^1 × 0.71 g ball used.

Noting the significantly improved performance of the mechanophotocatalysis methodology over the solution-state reactions conducted in air, we proceeded to explore whether this phenomenon was generalizable across a range of different substrates, Figure 5b. Noting the significant protodehalogenation byproduct obtained using the original conditions, we modified the catalyst loading ratio of the reaction to 2 mol % **[Ir1]** and 1 mol % **[Ni1]**, which afforded **3s** in a yield of 78 ± 4%; under these optimized conditions the protodehalogenated biproduct formed in only 9 ± 3% yield. By comparison, the aerobic solution-state reaction using these catalyst loadings still gave a much lower yield of 49%. A series of different primary and secondary alkyl bromides were then coupled with different aryl bromides under air both in solution and using the optimized mechanophotocatalysis methodology. Across 6 different coupling pairs, the mechanophotocatalysis conditions afforded a 12–52% increase in the product yield. Of particular note was the coupling between 3-bromopyridine with 4-bromotetrahydropyran; no product was obtained under the aerobic solution-state conditions, while under the mechanophotocatalysis conditions the target product **3x** was afforded in a yield of 45%. Unfortunately, the use of tertiary radicals remains an outstanding issue for this catalytic system, with both the solution-state and mechanophotocatalysis reaction conditions achieving low yields of 18% in 3 h. Interestingly, the solvent-minimized reaction had 54% of the starting material remaining, whereas there was almost full conversion of the starting material in the solution-state reaction. Together, these results indicate the potential value of mechanophotocatalysis to obviate the requirement to degas photocatalysis reactions, thereby streamlining reaction setup protocols.

### Preliminary Mechanistic Studies

Following these results, we considered how the photophysical properties of the photocatalyst could differ between solution and solvent-minimized conditions. Comparing photophysical parameters between the solid and solution states is understandably challenging; for instance, the Beer–Lambert law cannot be applied to a solvent-minimized environment, where light scattering from the surface of nontransparent materials is a dominating factor. The charge-transfer (CT) states of popular photocatalysts are also sensitive to both the environment (i.e., solvatochromism is observed as a function of solvent polarity) and concentration (due to possible aggregation), and these factors will influence their photophysical properties and thereby their reactivity.

We began by probing the photophysics of **[Ir1]**, Figure 6. The photoluminescence (PL) spectrum of **[Ir1]** remains broadly the same in both neat powder form and as a solution in DMA or DME, with only a slight red-shift of the emission onset for the solid sample, which would constitute a slight decrease in the excited-state energy of the photocatalyst in the absence of solvent, Figure 6a. This PL band reflects emission from a monomolecular species from a locally excited, ligand-centered (LC) state, as has previously been described for this complex.^90^ As an anaerobic solution in DMA, the emission of **[Ir1]** decays with monoexponential kinetics, with a lifetime, τ_PL_, of 1.8 μs (lit. τ_PL_ of 2.3 μs in MeCN^91^). Upon exposure to air, the τ_PL_ decreases by an order of magnitude to 190 ns, Figure 6b. Similarly, significant quenching of the PL lifetime was observed for a solution of **[Ir1]** in DME, with the τ_PL_ of **[Ir1]** decreasing by an order of magnitude from 2.1 μs to 200 ns under anaerobic and aerobic conditions, respectively. In contrast, in powder form, the intensity of the PL is much less attenuated in the presence of oxygen, with the τ_PL_ of **[Ir1]** decreasing by a factor of 1.55 (from 1.1 μs to 710 ns under anaerobic and aerobic conditions, respectively), Figure 6c. It is likely that this reduced quenching of the excited state under aerobic conditions in the absence of bulk solvent contributes to the improved performance of **[Ir1]** under mechanophotocatalysis conditions in the halogen atom transfer reaction, Figure 5. It is important to consider, however, that a potential improved tolerance to air of the nickel catalyst or some transient intermediate in the reaction system may also contribute to the increased yields observed from the metallaphotocatalysis reaction.

**Figure 6:**
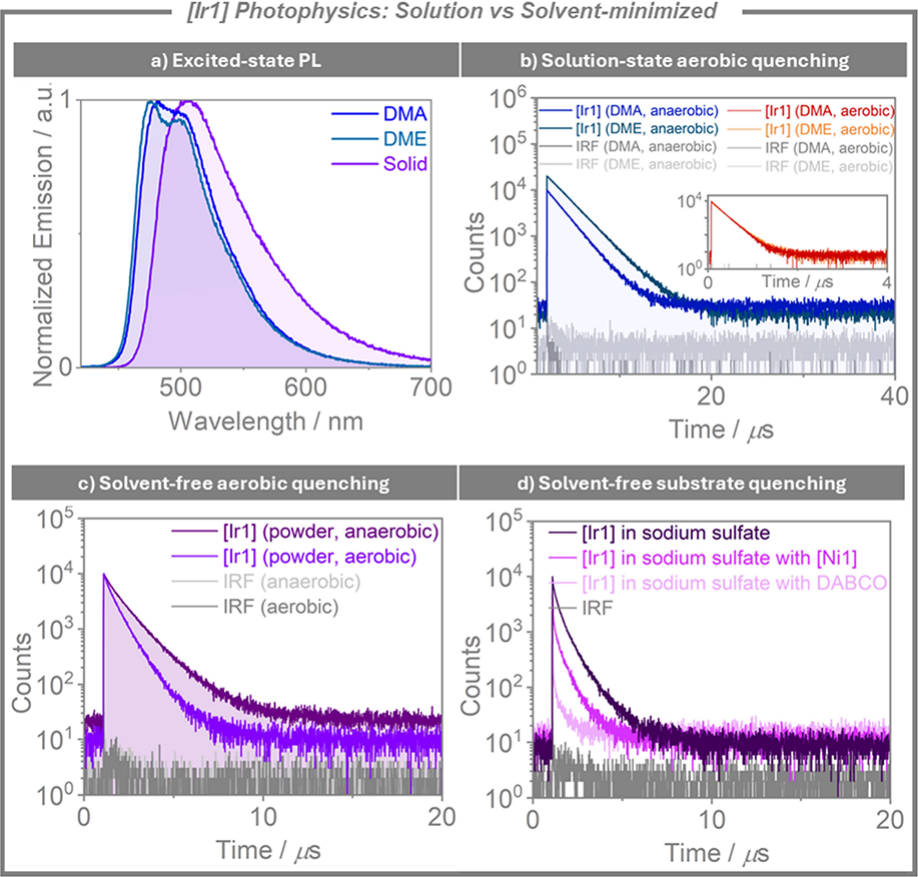
Photophysical property comparison of **[Ir1]** under solution-state and solid-state conditions. (a) Steady-state PL of **[Ir1]** as a powder and as a solution in DMA or DME (λ_exc_ = 380). (b) Time-resolved PL decay of **[Ir1]** in DMA or DME under degassed conditions and upon exposure to air (inset). Degassed solutions were prepared via bubbling with solvent-saturated nitrogen (λ_exc_ = 375 nm). (c) Time-resolved PL decay of **[Ir1]** powder under degassed conditions and under air. Powder samples were measured in a Young’s J NMR tube first under air, then under vacuum for the anaerobic measurements (λ_exc_ = 375 nm). (d) Quenching studies of **[Ir1]** with DABCO and **[Ni1]**. Conducted inside Eppendorfs (λ_exc_ = 375 nm). For full experimental details, see SI.

Subsequently, we sought to develop an approach to qualitatively observe quenching as a function of the interaction with different reaction components in the absence of solvent, Figure 6d. **[Ir1]** and the grinding auxiliary sodium sulfate were milled together, and the time-resolved PL-decay was measured. This was then repeated in the presence of different substrates (see SI for full details). Significant quenching was observed in the presence of DABCO, and in the presence of **[Ni1]**, while minimal quenching was observed for **2f**, and no quenching was observed for **1a**, which is consistent with the redox potentials and mechanistic roles of these substrates in reactions (see SI for details).

The aerobic quenching study was then expanded to other popular photocatalysts to explore how general this phenomena was, Figure 7. *fac*-Tris­(2-phenylpyridinato)­iridium­(III), *fac*-Ir­(ppy)_3_, (**[Ir2]**) possesses a τ_PL_ of 1.8 μs in MeCN (lit. τ_PL_ of 1.9 μs in 2-MeTHF^92^), which decreases by a factor of 95 to 19 ns upon exposure to air. In contrast, the τ_PL_ of a powder sample of this complex remains essentially the same under both aerobic and anaerobic conditions (0.26–0.27 μs). Tris­(2,2′-bipyridine)­ruthenium­(II) hexafluorophosphate, [Ru­(bpy)_3_]­[PF_6_]_2_ (**[Ru1**]) experiences a 6-fold decrease of its τ_PL_ upon exposure to air in MeCN (from 860 to 140 ns; lit. τ_PL_ of 825 ns under degassed conditions in MeCN^93^) while the τ_PL_ remains essentially unchanged as a powder (1.2 μs). The PL of **4CzIPN**, a compound that is thermally activated delayed fluorescent (TADF), decays with biexponential kinetics in both DMA solution and as a neat powder. Notably, the delayed fluorescence component, which originates from intersystem crossing/reverse intersystem crossing cycling between singlet and triplet excited states prior to emission, is much less quenched in powder form (1.9 and 1.6 μs under anaerobic and aerobic conditions, respectively) than in solution (2.3 μs and 790 ns under anaerobic and aerobic conditions, respectively; lit. τ_PL_ of 1.6 μs under anaerobic conditions in DMF^94^). Earth-abundant transition metal photocatalysts, especially those based on copper­(I), are popular alternatives to both expensive organic dyes such as **4CzIPN** and precious metal photocatalysts containing iridium or ruthenium.^8^ We selected [Cu­(dmp)­(xantphos)]­PF_6_ (**[Cu1]**) as an archetypal example of this class of photocatalysts. The behavior of the time-resolved PL decays of **[Cu1]** under degassed and aerated conditions mirrors that of the other catalysts; when going from degassed to aerated MeCN, the lifetime of **[Cu1]** decreases significantly from 2.1 × 10^2^ to 5.7 × 10^1^ ns (lit. τ_PL_ of 1.7 × 10^2^ ns in degassed MeCN^95^), whereas the powder sample possesses a significantly longer τ_PL_ that is insensitive to the presence of O_2_, (τ_PL_ of 2.3 × 10^4^ ns under both air and vacuum; lit. τ_PL_ of 3.0 × 10^4^ ns with tetrakis­(bis-3,5-trifluoromethylphenylborate) as the counteranion^96^). Thus, all five photocatalysts showed similar quenching behavior, and this attenuated PL quenching of these photocatalysts under aerobic conditions in the absence of bulk solvent likely contributes to mechanophotocatalysis unlocking an increased tolerance to aerobic reaction conditions.^74^

**Figure 7:**
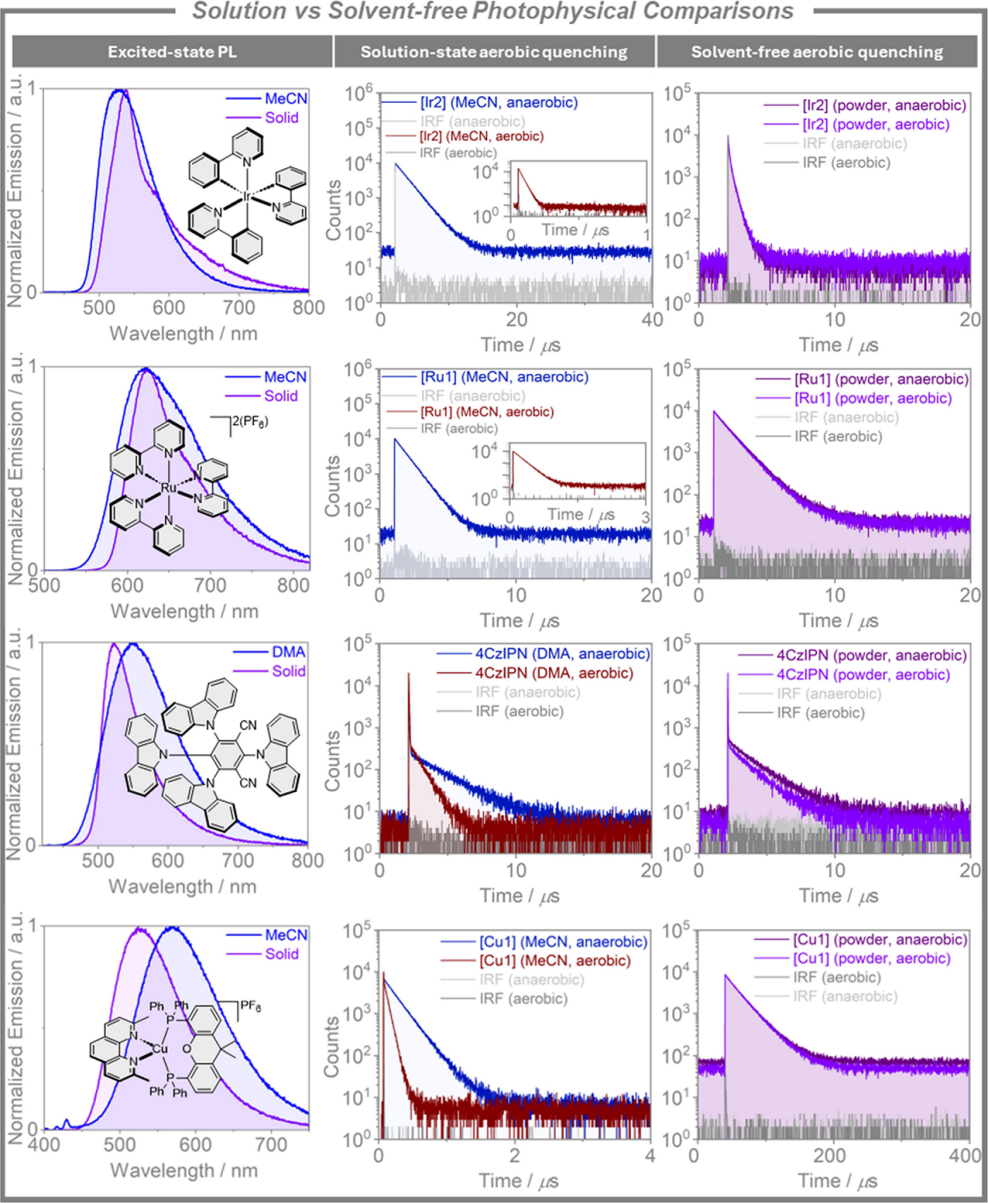
Comparison of the steady-state PL spectra (left column), solution-state time-resolved PL decays (center column) and solid-state (neat powder) time-resolved PL decays (right column) under aerobic and anaerobic conditions for four photocatalysts: **[Ir2]**, **[Ru1]**, **4CzIPN**, and **[Cu1]**. Degassed solutions were prepared by bubbling with solvent-saturated nitrogen for 30 min. Neat powder samples were measured in a Young’s J NMR tube first under air, then under vacuum for the anaerobic measurements. λ_exc_ = 380 or 375 nm for the steady-state PL and time-resolved PL measurements, respectively. For full experimental details, see SI.

## Conclusions

This report demonstrates the first examples of metallaphotoredox reactions being transmuted from the solution-state to a solvent-minimized mechanochemically mediated environment. High-yielding aryl aminations and C­(sp2)-C­(sp3) cross-couplings with carboxylic acids, trifluoroborate salts, and alkyl bromides were conducted under aerobic conditions with significant reductions in the use and waste of reaction solvents. Notably, these C­(sp2)-C­(sp3) cross-coupling partners have not yet been successfully employed as radical precursors in other solvent-minimized methodologies (such as mechanoredox chemistry and mechanochemistry using zerovalent metal reductants), showcasing the value of photocatalysis in offering a more diverse substrate range as radical precursors. Optimization of the reaction rheology and mixing efficiency by varying the auxiliary and LAG loadings was an important consideration for improving reaction efficiency, increasing the number of parameters that must be considered for optimizing reactions. However, the screening process of small-scale photocatalysis reactions is expedited by this new approach. High boiling point solvents such as DMA are challenging to remove and typically require extraction workups before analysis, whereas the solvent-minimized approach can be rapidly processed for analysis via a simple filtration. The ability to forego the degassing of reactions, as exemplified by the cross-electrophile coupling reaction study, also enables potentially faster screening via a simplified reaction setup procedure. The disclosure of increased throughput holder designs and cheap reaction vessels increases the accessibility of this type of chemistry. Through time-resolved photoluminescence quenching studies, we observed that five popular photocatalysts were much more sensitive to oxygen in solution as compared to as powders, which likely contributes to the improved performance of the mechanometallaphotocatalysis reactions conducted in air. This work solidifies mechanophotocatalysis as an accessible and versatile tool for mediating solvent-minimized photocatalysis reactions.

## Supplementary Material



## Data Availability

The research data supporting this publication can be accessed at https://doi.org/10.17630/dac13f46-01e9-467d-8366-0f4e7499c0ca.

## Notes

Experimental methodology, details of the mechanophotocatalysis and solution-state reactors and reaction vessels, design specifications for the increased throughput holder, measurement details, ^1^H and ^19^F NMR spectra (PDF)
