# Supplementary material for: Mechanometallaphotoredox Catalysis: Utilizing Increased Throughput Mechanochemistry to Develop Solvent-Minimized Aryl Amination and C(sp2)-C(sp3) Cross-Coupling Reactions with Increased Tolerance to Aerobic Conditions
Source: J Am Chem Soc. 2025 May 22;147(26):22919–31. doi: 10.1021/jacs.5c05503 (PMC12232315; doi:10.1021/jacs.5c05503)
Supplement: Supplementary file 1 [file ja5c05503_si_001.pdf]

Supplementary Materials

Mechanometallaphotoredox Catalysis: Utilizing Increased Through-put Mechanochemistry to Develop Solvent-minimized Aryl Amination and C(sp2)-C(sp3) Cross-coupling Reactions with Increased Tolerance to Aerobic Conditions

Francis Millward, Eli Zysman-Colman*

*Corresponding author: eli.zysman-colman@st-andrews.ac.uk

Organic Semiconductor Centre, EaStCHEM School of Chemistry, University of St Andrews; St Andrews, KY16 9ST, United Kingdom.

Materials and Methods

General synthetic procedures

The following catalysts and starting materials were synthesised according to literature procedures: [Ir(dF(CF$_3$)ppy)$_2$(dtbbpy)]PF$_6$ (**[Ir1]**),[1] [Ni(dtbbpy)(OH$_2$)$_4$]Cl$_2$ (**[Ni1]**),[2] [Cu(dmp)(xantphos)]PF$_6$ (**[Cu1]**).[3] All other reagents and solvents were obtained from commercial suppliers and used as received. Air-sensitive reactions were performed under a nitrogen atmosphere using Schlenk techniques. No special precautions were taken to exclude air or moisture during work-ups or purifications for mechanochemical reactions. Mechanophotocatalysis reactions were conducted using a modified *Retch MM400* ball mill equipped with a steal cover that permits light irradiation from above.[4] Solution-state reactions were conducted in microwave vials using a holder designed by the Leonori group[5] inside a custom photoreactor.[6] Flash column chromatography was carried out using a CombiFlash NextGen automated column from Teledyne ISCO, with solid loading of samples using silica gel (Silia-P from Silicycle, 60 Å, 40-63 µm). Analytical thin-layer chromatography (TLC) was performed with silica plates with aluminium backings (250 µm with F-254 indicator). TLC visualization was accomplished by 254/365 nm UV lamp. Melting points were measured using open-ended capillaries on an Electrothermal 1101D Mel-Temp apparatus and are uncorrected. GCMS analysis was conducted using a Shimadzu QP2010SE GC-MS equipped with a Shimadzu SH-Rtx-1 column (30 m × 0.25 mm). ^{1}H spectra were recorded on a Bruker Advance spectrometer (400 or 500 MHz for ^{1}H, 470 or 377 MHz for ^{19}F, 126 MHz for ^{13}C). The following abbreviations have been used for multiplicity assignments: "s" for singlet, "d" for doublet, "t" for triplet, "q" for quartet, "m" for multiplet, "br" for broad, and qC for quaternary carbon. ^{1}H spectra were referenced against residual solvent peaks with respect to TMS (δ = 0 ppm).

General procedure for aerobic mechanophotocatalysis experiments. A 2 mL transparent polypropylene Eppendorf was charged with the catalysts and reagents (solids and liquids), sodium sulfate as a grinding auxiliary, and milling balls; either 12 × 0.26 g, 3 × 0.52 g, 1 × 0.71 g, or 1 × 0.89 g. The Eppendorf was closed, secured in the V1 or V2 holder, (Figure **S4**), briefly shaken by hand and tapped on the bench to premix the reagents, and locked into a *Retch MM400* ball mill modified with a steal cover that permits light irradiation from above. The mixture was pre-milled for 5-10 seconds in the absence of light, before being milled at 25 Hz while being irradiated by a *Kessil* LED (λ_{max} = 456 nm) for the desired reaction time.

General procedure for aerobic solution-state photocatalysis reactions. An oven-dried microwave reactor vial was charged with the catalysts and reagents (solids and liquids), solvent, and a magnetic stir bar. The vial was plugged with a septum with a bleed needle, placed into the solution-state photoreactor, and stirred while being irradiated by a *Kessil* LED (λ_{max} = 456 nm).

General procedure for anaerobic solution-state photocatalysis reactions. An oven-dried microwave reactor vial was charged with the catalysts and reagents (solids only), and a magnetic stir bar. The vial was closed with a crimp lid, and purged with nitrogen (5 minutes). Liquid reagents and solvents were added via syringe under the flow of nitrogen, before the solution was bubbled with nitrogen (10 minutes). The vial was placed into the solution-state photoreactor, and stirred while being irradiated by a *Kessil* LED (λ_{max} = 456 nm).

Quantitative ¹H NMR spectroscopy analysis and isolation. Crude reaction mixtures were filtered through a celite plug with the minimal volume of ethyl acetate, and the solvent was removed under reduced pressure. Alternatively, the crude mixtures can be diluted with water/brine (1:1) and washed with ethyl acetate, dried over anhydrous sodium sulfate, filtered, and the solvent removed under reduced pressure. The crude could then be subjected to either quantitative ^{1}H NMR spectroscopy analysis or purification. Quantitative ^{1}H NMR spectroscopy analysis was conducted using 1,3,5-trimethoxybenezene as an internal standard. The crude products were purified by flash column chromatography (slowly increasing gradient of ethyl acetate:petroleum ether). Isolated product ^{1}H and ^{19}F NMR spectra are from mechanophotocatalysis experiments.

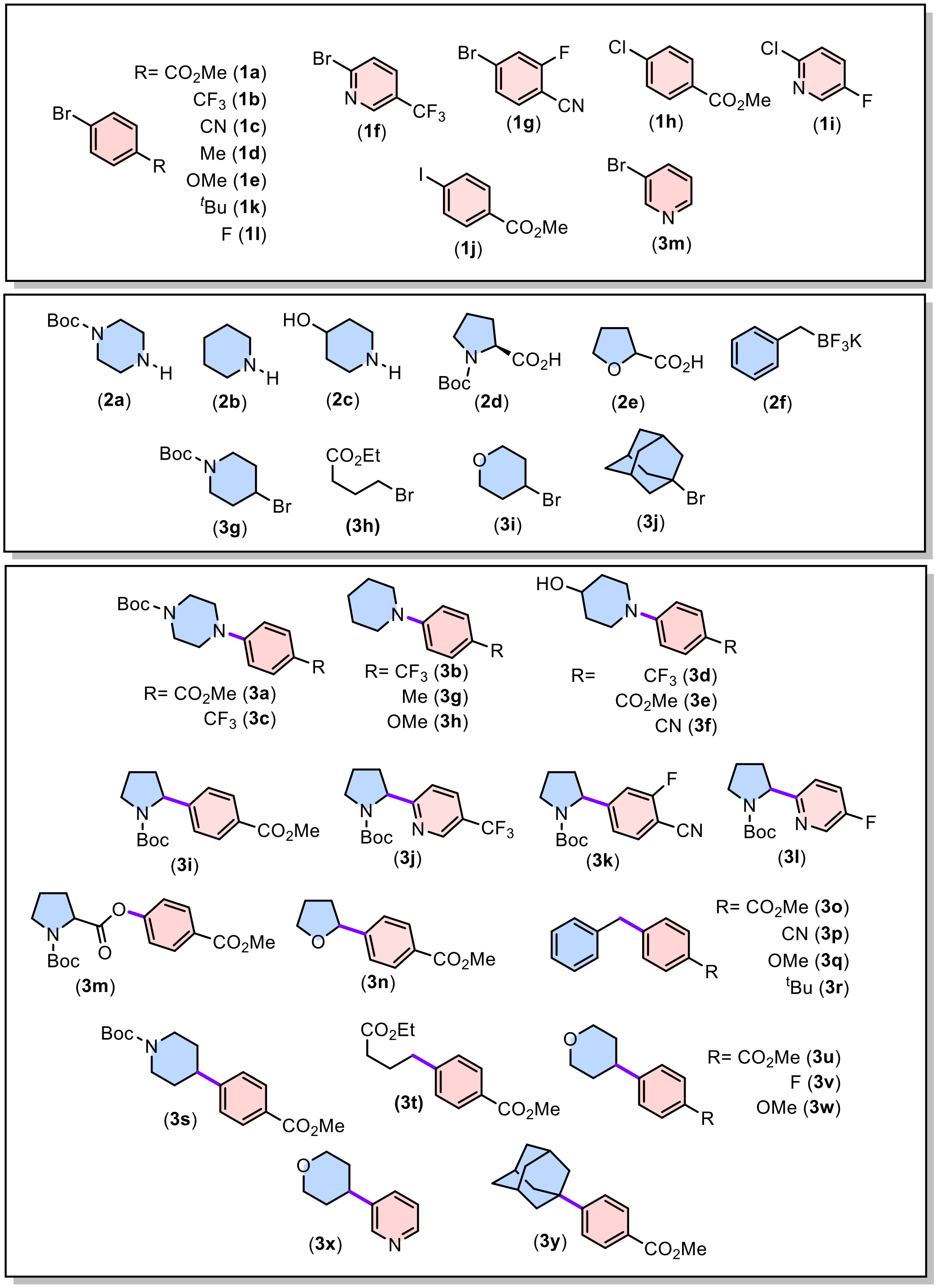

Figure **S1.** Substrates and products in this study.

*Synthesis of (**3a**).* Conducted according to the general mechanophotocatalysis and solution-state procedures. Optimised mechanophotocatalysis procedure utilised methyl 4-bromobenzoate (64.5 mg, 1 equiv., 0.3 mmol), *tert*-butyl-1-piperazinecarboxylate (111.8 mg, 2 equiv., 0.6 mmol), DABCO (67.3 mg, 2 equiv., 0.6 mmol), [Ir(dF(CF_3)ppy))$_2$(dtbbpy)]PF_6 (3.4 mg, 0.01 equiv., 0.003

mmol), nickel(II) bromide•DME (4.63 mg, 0.05 equiv., 0.015 mmol), *N,N*-dimethylacetamide (39.2 mg, 42 µL, 1.5 equiv., 0.45 mmol), sodium sulfate (767.0 mg, 18 equiv., 5.4 mmol), and 1 × 0.89 g milling ball for 3 hours. Solution-state photocatalysis experiments used *N,N*-dimethylacetamide (1.2 mL, 0.25 M with respect to the limiting reagent) instead of sodium sulfate. The crude product was purified by flash column chromatography (0% → 10% ethyl acetate:petroleum ether) to afford the final product, *tert*-butyl 4-(4-(methoxycarbonyl)phenyl)piperazine-1-carboxylate, as a white solid. **^{1}H NMR (400 MHz, $CDCl_3$) δ (ppm)**: 7.96-7.89 (m, 2H), 6.89-6.82 (m, 2H), 3.86 (s, 3H), 3.62-3.51 (m, 4H), 3.35-3.23 (m, 4H), 1.48 (s, 9H). The ^{1}H NMR spectrum is consistent with the literature.[7]

*Synthesis of (**3b**).* Conducted according to the general mechanophotocatalysis procedure. Optimised mechanophotocatalysis procedure utilised 1-bromo-4-(trifluoromethyl)benzene (67.5 mg, 1 equiv., 0.3 mmol), piperidine (51.1 mg, 2 equiv., 0.6 mmol), DABCO (67.3 mg, 2 equiv., 0.6 mmol), $[Ir(dF(CF_3)ppy))_2(dtbbpy)]PF_6$ (1.0 mg, 0.003 equiv., 0.9 µmol), nickel(II) bromide• DME (4.63 mg, 0.05 equiv., 0.015 mmol), *N,N*-dimethylacetamide (39.2 mg, 42 µL, 1.5 equiv., 0.45 mmol), sodium sulfate (767.0 mg, 18 equiv., 5.4 mmol), and 1 × 0.89 g milling ball for 3 hours. The crude product was purified by flash column chromatography (100% petroleum ether) to afford the final product, 1-(4-(trifluoromethyl)phenyl)piperidine, as a colourless oil. **^{1}H NMR (400 MHz, $CDCl_3$) δ (ppm)**: 7.48-7.42 (m, 2H), 6.94-6.88 (m, 2H), 3.30-3.32 (m, 4H), 1.73-1.58 (m, 6H). **^{19}F NMR (470 MHz, $CDCl_3$) δ (ppm):** -61.21 (s, 3F). The ^{1}H and ^{19}F NMR spectra are consistent with the literature.[8]

*Synthesis of (**3c**).* Conducted according to the general mechanophotocatalysis procedure. Mechanophotocatalysis procedure utilised 1-bromo-4-(trifluoromethyl)benzene (67.5 mg, 1 equiv., 0.3 mmol), *tert*-butyl-1-piperazinecarboxylate (111.8 mg, 2 equiv., 0.6 mmol), DABCO (67.3 mg, 2 equiv., 0.6 mmol), $[Ir(dF(CF_3)ppy))_2(dtbbpy)]PF_6$ (3.4 mg, 0.01 equiv., 0.003 mmol), nickel(II) bromide DME (4.63 mg, 0.05 equiv., 0.015 mmol), *N,N*-dimethylacetamide (39.2 mg, 42 µL, 1.5 equiv., 0.45 mmol), sodium sulfate (767.0 mg, 18 equiv., 5.4 mmol), and 1 × 0.89 g milling ball for 3 hours. The crude product was purified by flash column chromatography (0% → 5% ethyl acetate:petroleum ether) to afford the final product, *tert*-butyl 4-(4-(trifluoromethyl)phenyl)piperazine-1-carboxylate, as a white solid. **^{1}H NMR (400 MHz, $CDCl_3$) δ (ppm)**: 7.52-7.46 (m, 2H), 6.95-6.89 (m, 2H), 3.62-3.55 (m, 4H), 3.28-3.21 (m, 4H), 1.49 (s, 9H). **^{19}F NMR (470 MHz, $CDCl_3$) δ (ppm):** -61.45 (s, 3F). The ^{1}H and ^{19}F NMR spectra are consistent with the literature.[9]

*Synthesis of (**3d**).* Conducted according to the general mechanophotocatalysis procedure. The mechanophotocatalysis procedure utilised 1-bromo-4-(trifluoromethyl)benzene (67.5 mg, 1 equiv., 0.3 mmol), 4-hydroxypiperidine (60.7 mg, 2 equiv., 0.6 mmol), DABCO (67.3 mg, 2 equiv., 0.6 mmol), $[Ir(dF(CF_3)ppy))_2(dtbbpy)]PF_6$ (0.34 mg, 0.001 equiv., 0.3 µmol), nickel(II) bromide DME (4.63 mg, 0.05 equiv., 0.015 mmol), *N,N*-dimethylacetamide (39.2 mg, 42 µL, 1.5 equiv., 0.45 mmol), sodium sulfate (767.0 mg, 18 equiv., 5.4 mmol), and 1 × 0.89 g milling ball for 3 hours. The crude product was purified by flash column chromatography (0% → 34% ethyl acetate:petroleum ether) to afford the final product, 1-(4-(trifluoromethyl)phenyl)piperidin-4-ol, as a white solid. **^{1}H NMR (400 MHz, $CDCl_3$) δ (ppm)**: 7.49-7.46 (m, 2H), 6.96-6.90 (m, 2H), 3.96-3.87 (m, 1H), 3.72-3.60 (m, 2H), 3.10-3.00 (m, 2H), 2.05-1.96 (m, 2H), 1.72-1.60 (m, 2H),

1.47 (d, $J = 4.2$ Hz, 1H). **^{19}F NMR (377 MHz, $CDCl_3$) δ (ppm):** -61.32. The ^{1}H and ^{19}F NMR spectra are consistent with the literature.[8]

Synthesis of (3e). Conducted according to the general mechanophotocatalysis procedure. The mechanophotocatalysis procedure utilised methyl 4-bromobenzoate (64.5 mg, 1 equiv., 0.3 mmol), 4-hydroxypiperidine (60.7 mg, 2 equiv., 0.6 mmol), DABCO (67.3 mg, 2 equiv., 0.6 mmol), $[Ir(dF(CF_3)ppy))_2(dtbbpy)]PF_6$ (0.34 mg, 0.001 equiv., 0.3 μmol), nickel(II) bromide DME (4.63 mg, 0.05 equiv., 0.015 mmol), *N,N*-dimethylacetamide (39.2 mg, 42 μL, 1.5 equiv., 0.45 mmol), sodium sulfate (767.0 mg, 18 equiv., 5.4 mmol), and 1 × 0.89 g milling ball for 3 hours. The crude product was purified by flash column chromatography (0% → 40% ethyl acetate:petroleum ether) to afford the final product, methyl 4-(4-hydroxypiperidin-1-yl)benzoate, as a white solid. **^{1}H NMR (400 MHz, $CDCl_3$) δ (ppm)**: δ 7.93-7.87 (m, 2H), 6.90-6.84 (m, 2H), 3.97-3.89 (m, 1H), 3.86 (s, 3H), 3.78-3.68 (m, 2H), 3.15-3.04 (m, 2H), 2.04-1.95 (m, 2H), 1.71-1.59 (m, 2H), 1.49 (d, $J = 4.2$ Hz, 1H). **^{13}C NMR (126 MHz, $CDCl_3$) δ (ppm):** 167.33 (qC), 153.96 (qC), 131.42 (CH), 119.29 (qC), 113.89 (CH), 67.69 (CH or CH_3), 51.75 (CH or CH_3), 45.54 (CH_2), 33.78 (CH_2). The ^{1}H spectrum differs from the literature report,[10] but the ^{1}H, ^{13}C NMR and GCMS spectra are consistent with molecular structure.

Synthesis of (3f). Conducted according to the general mechanophotocatalysis procedure. The mechanophotocatalysis procedure utilised 4-bromobenzonitrile (54.6 mg, 1 equiv., 0.3 mmol), 4-hydroxypiperidine (60.7 mg, 2 equiv., 0.6 mmol), DABCO (67.3 mg, 2 equiv., 0.6 mmol), $[Ir(dF(CF_3)ppy))_2(dtbbpy)]PF_6$ (0.34 mg, 0.001 equiv., 0.3 μmol), nickel(II) bromide DME (4.63 mg, 0.05 equiv., 0.015 mmol), *N,N*-dimethylacetamide (39.2 mg, 42 μL, 1.5 equiv., 0.45 mmol), sodium sulfate (767.0 mg, 18 equiv., 5.4 mmol), and 1 × 0.89 g milling ball for 3 hours. The crude product was purified by flash column chromatography (0% → 40% ethyl acetate:petroleum ether) to afford the final product, 4-(4-hydroxypiperidin-1-yl)benzonitrile, as a white solid. **^{1}H NMR (400 MHz, $CDCl_3$) δ (ppm)**: 7.51-7.44 (m, 2H), 6.89-6.82 (m, 2H), 3.99-3.90 (m, 1H), 3.76-3.63 (m, 2H), 3.19-3.05 (m, 2H), 2.05-1.94 (m, 2H), 1.70-1.55 (m, 3H). The ^{1}H spectrum is consistent with the literature.[11]

Synthesis of (3g). Conducted according to the general mechanophotocatalysis procedure. The mechanophotocatalysis procedure utilised 4-bromotoluene (51.3 mg, 1 equiv., 0.3 mmol), piperidine (204 mg, 8 equiv., 0.6 mmol), DABCO (67.3 mg, 2 equiv., 0.6 mmol), $[Ir(dF(CF_3)ppy))_2(dtbbpy)]PF_6$ (0.34 mg, 0.00017 equiv., 0.051 μmol), nickel(II) bromide DME (9.26 mg, 0.1 equiv., 0.03 mmol), *N,N*-dimethylacetamide (39.2 mg, 42 μL, 1.5 equiv., 0.45 mmol), sodium sulfate (767.0 mg, 18 equiv., 5.4 mmol), and 1 × 0.89 g milling ball for 4.5 hours. The crude product was purified by flash column chromatography (0% → 5% ethyl acetate:petroleum ether) to afford the final product, 1-(*p*-tolyl)piperidine, as a colourless oil. **^{1}H NMR (400 MHz, $CDCl_3$) δ (ppm)**: 7.09-7.03 (m, 2H), 6.90-6.83 (m, 2H), 3.14-3.04 (m, 4H), 2.27 (s, 3H), 1.76-1.67 (m, 4H), 1.62-1.52 (m, 2H). The ^{1}H spectrum is consistent with the literature.[8]

Synthesis of (3h). Conducted according to the general mechanophotocatalysis procedure. The mechanophotocatalysis procedure utilised 4-bromotoluene (51.3 mg, 1 equiv., 0.3 mmol), piperidine (204 mg, 8 equiv., 0.6 mmol), DABCO (67.3 mg, 2 equiv., 0.6 mmol), $[Ir(dF(CF_3)ppy))_2(dtbbpy)]PF_6$ (0.34 mg, 0.00017 equiv., 0.051 μmol), nickel(II) bromide DME (9.26 mg, 0.1 equiv., 0.03 mmol), *N,N*-dimethylacetamide (39.2 mg, 42 μL, 1.5 equiv., 0.45

mmol), sodium sulfate (767.0 mg, 18 equiv., 5.4 mmol), and 1 × 0.89 g milling ball for 4.5 hours. Due to the low yield and small scale of the reaction, the product was not isolated. The following signals in the ^{1}H spectrum were used to calculate the yield, and is consistent with the literature:[12] **^{1}H NMR (400 MHz, $CDCl_3$) δ (ppm)**: 6.91 (m, 2H). The presence of the desired compound, 1-(4-methoxyphenyl)piperidine, was confirmed via GCMS.

Method for low catalyst loading addition. During the synthesis of **3d-h**, the small masses of **[Ir1]** were too inaccurate to weigh out directly. Thus, these were added as a stock solution in the LAG agent. A stock mixture of DMA (210 μL) and **[Ir1]** (1.6 mg) were mixed via sonication. For 0.1 mol%: 42 μL of the stock was added for the correct LAG and photocatalyst loadings. For 0.017 mol%: 7 μL of the stock and 35 μL DMA were added for the correct LAG and photocatalyst loadings.

Synthesis of (3i). Conducted according to the general mechanophotocatalysis and solution-state procedures. Optimised mechanophotocatalysis procedure utilised methyl 4-bromobenzoate (21.5 mg, 1 equiv., 0.1 mmol), (*tert*-butoxycarbonyl)proline (32.3 mg, 1.5 equiv., 0.15 mmol), caesium carbonate (48.9 mg, 1.5 equiv., 0.15 mmol), $[Ir(dF(CF_3)ppy))_2(dtbbpy)]PF_6$ (1.1 mg, 0.01 equiv., 1.0 μmol), $[Ni(dtbbpy)(OH_2)_4]Cl_2$ (4.7 mg, 0.1 equiv., 10 μmol), sodium sulfate (128 mg, 9 equiv., 0.9 mmol), *N,N*-dimethylacetamide (26.1 mg, 28 μL, 3 equiv., 0.3 mmol), and 1 × 0.89 g milling ball for 2 hours. Solution-state photocatalysis experiments used *N,N*-dimethylacetamide (1 mL for 0.1 M, 5 mL for 0.02 M) instead of sodium sulfate. The crude product was purified by flash column chromatography (0% → 12% ethyl acetate:petroleum ether) to afford the final product, tert-butyl 2-(4-(methoxycarbonyl)phenyl)pyrrolidine-1-carboxylate, as a colourless oily solid. **^{1}H NMR (400 MHz, $CDCl_3$) δ (ppm)**: 8.01-7.93 (m, 2H), 7.25-7.20 (m, 2H), 4.96 (br s, 0.3H, rotamer), 4.79 (br s, 0.7H, rotamer), 3.90 (s, 3H), 3.69-3.47 (m, 2H), 2.45-2.23 (m, 1H), 1.97-1.74 (m, 3H), 1.44 (s, 3H), 1.16 (s, 6H). The ^{1}H NMR spectrum is consistent with the literature.[13] Note: **3i** could also be synthesised starting from methyl 4-chlorobenzoate (17.1 mg, 1 equiv., 0.1 mmol) using the modified conditions: aux. (15 equiv.), LAG (6 equiv.), 1 x 0.71 g ball, 4.5 h.

Synthesis of (3j). Conducted according to the general mechanophotocatalysis and solution-state procedures. Mechanophotocatalysis procedure utilised 2-bromo-5-(trifluoromethyl)pyridine (22.6 mg, 1 equiv., 0.1 mmol), (*tert*-butoxycarbonyl)proline (32.3 mg, 1.5 equiv., 0.15 mmol), caesium carbonate (48.9 mg, 1.5 equiv., 0.15 mmol), $[Ir(dF(CF_3)ppy))_2(dtbbpy)]PF_6$ (1.1 mg, 0.01 equiv., 1.0 μmol), $[Ni(dtbbpy)(OH_2)_4]Cl_2$ (4.7 mg, 0.1 equiv., 10 μmol), sodium sulfate (213 mg, 15 equiv., 1.5 mmol), *N,N*-dimethylacetamide (26.1 mg, 28 μL, 3 equiv., 0.3 mmol), and 1 × 0.89 g milling ball for 3 hours. The crude product was purified by flash column chromatography (0% → 9% ethyl acetate:petroleum ether) to afford the final product, *tert*-butyl-2-(5-(trifluoromethyl)pyridin-2-yl)pyrrolidine-1-carboxylate, as a colourless oily solid. **^{1}H NMR (500 MHz, $CDCl_3$) δ (ppm)**: 8.79 (s, 1H), 7.92-7.80 (m, 1H), 7.34-7.27 (m, 1H), 5.07-4.97 (m, 0.4H, rotamer). 4.92 (1H, br s, rotamer), 3.72-3.47 (m, 2H), 2.49-2.26 (m, 1H), 2.11-1.80 (m, 3H), 1.44 (s, 4H), 1.19 (s, 5H). **^{19}F NMR (470 MHz, $CDCl_3$) δ (ppm)**: -62.19 (s), -62.26 (s) (rotamer mixture (3:2)). The ^{1}H NMR spectrum is consistent with the literature.[13]

Synthesis of (3k). Conducted according to the general mechanophotocatalysis procedure. The mechanophotocatalysis procedure utilised 4-bromo-2-fluorobenzonitrile (20.0 mg, 1 equiv., 0.1

mmol), (tert-butoxycarbonyl)proline (32.3 mg, 1.5 equiv., 0.15 mmol), caesium carbonate (48.9 mg, 1.5 equiv., 0.15 mmol), $[Ir(dF(CF_3)ppy))_2(dtbbpy)]PF_6$ (1.1 mg, 0.01 equiv., 1.0 μmol), $[Ni(dtbbpy)(OH_2)_4]Cl_2$ (4.7 mg, 0.1 equiv., 10 μmol), sodium sulfate (213 mg, 15 equiv., 1.5 mmol), *N,N*-dimethylacetamide (52.3 mg, 55.5 μL, 6 equiv., 0.6 mmol), and 1 × 0.71 g milling ball for 4.5 hours. The crude product was purified by flash column chromatography (0% → 20% ethyl acetate:petroleum ether) to afford the final product, tert-butyl 2-(4-cyano-3-fluorophenyl)pyrrolidine-1-carboxylate, as a colourless oil. **^{1}H NMR (400 MHz, $CDCl_3$) δ (ppm)**: 7.56 (t, *J* = 7.3 Hz, 1H), 7.03 (d, *J* = 10 Hz, 1H), 7.08 (dd, *J* = 8.1, 1.2 Hz, 1H), 4.92 (s, 0.4H, rotamer), 4.78 (s, 0.6H, rotamer), 3.74-3.50 (m, 2H), 2.45-2.28 (m, 1H), 1.98-1.84 (m, 2H), 1.84-1.70 (m, 1H), 1.45 (br s, 4H, rotamer), 1.21 (br s, 5H, rotamer). **^{19}F NMR (377 MHz, $CDCl_3$) δ (ppm)**: -106.55 (s), -106.50 (s) (rotamer mixture (4:5)). The ^{1}H NMR spectrum is consistent with the literature.[14]

*Synthesis of (**3l**).* Conducted according to the general mechanophotocatalysis procedure. The mechanophotocatalysis procedure utilised 2-chloro-5-fluoropyridine (13.2 mg, 1 equiv., 0.1 mmol), (tert-butoxycarbonyl)proline (32.3 mg, 1.5 equiv., 0.15 mmol), caesium carbonate (48.9 mg, 1.5 equiv., 0.15 mmol), $[Ir(dF(CF_3)ppy))_2(dtbbpy)]PF_6$ (1.1 mg, 0.01 equiv., 1.0 μmol), $[Ni(dtbbpy)(OH_2)_4]Cl_2$ (4.7 mg, 0.1 equiv., 10 μmol), sodium sulfate (213 mg, 15 equiv., 1.5 mmol), *N,N*-dimethylacetamide (52.3 mg, 55.5 μL, 6 equiv., 0.6 mmol), and 1 × 0.71 g milling ball for 4.5 hours. The crude product was purified by flash column chromatography (0% → 20% ethyl acetate:petroleum ether) to afford the final product, *tert*-butyl-2-(5-fluoropyridin-2-yl)pyrrolidine-1-carboxylate, as a colourless oil. **^{1}H NMR (400 MHz, $CDCl_3$) δ (ppm)**: 8.38 (d, *J* = 2.9 Hz, 1H), 7.35 (m, 1H), 7.17 (m, 1H), 4.97 (br s, 0.4H, rotamer), 4.86 (br s, 0.6H, rotamer), 3.72-3.45 (m, 2H), 2.44-2.18 (m, 1H), 2.10-1.81 (m, 3H), 1.45 (s, 4H, rotamer), 1.21 (s, 5H, rotamer). **^{19}F NMR (377 MHz, $CDCl_3$) δ (ppm)**: -130.56 (s), -130.75 (s) (rotamer mixture (1.7:1)). The ^{1}H NMR spectrum is consistent with the literature.[14]

*Synthesis of (**3m**).* Conducted according to the general mechanophotocatalysis procedure. The mechanophotocatalysis procedure utilised methyl 4-iodobenzoate (26.2 mg, 1 equiv., 0.1 mmol), (tert-butoxycarbonyl)proline (32.3 mg, 1.5 equiv., 0.15 mmol), caesium carbonate (48.9 mg, 1.5 equiv., 0.15 mmol), $[Ir(dF(CF_3)ppy))_2(dtbbpy)]PF_6$ (1.1 mg, 0.01 equiv., 1.0 μmol), $[Ni(dtbbpy)(OH_2)_4]Cl_2$ (4.7 mg, 0.1 equiv., 10 μmol), sodium sulfate (213 mg, 15 equiv., 1.5 mmol), *N,N*-dimethylacetamide (52.3 mg, 55.5 μL, 6 equiv., 0.6 mmol), and 1 × 0.71 g milling ball for 4.5 hours. The crude product was purified by flash column chromatography (0% → 10% ethyl acetate:petroleum ether) to afford the final product, 1-(*tert*-butyl)-2-(4-(methoxycarbonyl)phenyl) pyrrolidine-1,2-dicarboxylate, as a colourless oil. **^{1}H NMR (400 MHz, $CDCl_3$) δ (ppm)**: 8.11-8.00 (m, 2H), 7.24-7.12 (m, 2H), 4.55-4.41 (m, 1H), 3.91 and 3.90 (s, 3H, rotamer), 3.68-3.40 (m, 2H), 2.47-2.26 (m, 1H), 2.26-2.10 (m, 1H), 2.10-1.88 (m, 2H), 1.47 and 1.44 (s, 9H, rotamer). The ^{1}H NMR spectrum is consistent with the literature.[15]

*Synthesis of (**3n**).* Conducted according to the general mechanophotocatalysis procedure. The mechanophotocatalysis procedure utilised methyl 4-bromobenzoate (21.5 mg, 1 equiv., 0.1 mmol), tetrahydrofuran-2-carboxylic acid (34.8 mg, 3 equiv., 0.3 mmol), potassium phthalimide (55.6 mg, 3 equiv., 0.3 mmol), $[Ir(dF(CF_3)ppy))_2(dtbbpy)]PF_6$ (1.1 mg, 0.01 equiv., 1.0 μmol), $[Ni(dtbbpy)(OH_2)_4]Cl_2$ (4.7 mg, 0.1 equiv., 10 μmol), sodium sulfate (213 mg, 15 equiv., 1.5 mmol), *N,N*-dimethylacetamide (52.3 mg, 55.5 μL, 6 equiv., 0.6 mmol), and 1 × 0.71 g milling

ball for 4.5 hours. The crude product was purified by flash column chromatography (0% → 3% ethyl acetate:petroleum ether) to afford the final product, methyl 4-(tetrahydrofuran-2-yl)benzoate, as a colourless oil. **^{1}H NMR (400 MHz, $CDCl_3$) δ (ppm)**: 8.03-7.97 (m, 2H), 7.45-7.35 (m, 2H), 4.94 (t, *J* = 7.2 Hz, 1H), 4.17-4.05 (m, 1H), 4.00-3.93 (m, 1H), 3.91 (s, 3H), 2.45-2.30 (m, 1H), 2.10-1.95 (m, 2H), 1.88-1.71 (m, 1H). The ^{1}H NMR spectrum is consistent with the literature. [13]

*Synthesis of (**3o**).* Conducted according to the general mechanophotocatalysis and solution-state procedures. Mechanophotocatalysis procedure utilised methyl 4-bromobenzoate (43.0 mg, 1 equiv., 0.2 mmol), potassium benzyltrifluoroborate (47.5 mg, 1.2 equiv., 0.24 mmol), [Ir(dF(CF3)ppy))2(dtbbpy)]PF_6 (4.5 mg, 0.02 equiv., 4 μmol), [Ni(dtbbpy)$(OH_2)_4$]Cl_2 (2.8 mg, 0.03 equiv., 6 μmol), 2,6-lutidine (75.0 mg, 3.5 equiv., 0.7 mmol), *N,N*-dimethylacetamide (52.3 mg, 56 μL, 3 equiv., 0.6 mmol), sodium sulfate (426 mg, 15 equiv., 3 mmol), and 1 × 0.89 g milling ball for 3 hours. The crude product was purified by flash column chromatography (100% petroleum ether) to afford the final product, methyl 4-benzylbenzoate, as a colourless oil. **^{1}H NMR (400 MHz, $CDCl_3$) δ (ppm)**: 7.99-7.93 (m, 2H), 7.33-7.19 (m, 5H), 7.19-7.15 (m, 2H), 4.03 (m, 2H), 3.90 (s, 3H). The ^{1}H NMR spectrum is consistent with the literature.[13]

*Synthesis of (**3p**).* Conducted according to the general mechanophotocatalysis and solution-state procedures. Mechanophotocatalysis procedure utilised 4-bromobenzonitrile (36.4 mg, 1 equiv., 0.2 mmol), potassium benzyltrifluoroborate (47.5 mg, 1.2 equiv., 0.24 mmol), [Ir(dF(CF3)ppy)$)_2$(dtbbpy)]PF_6 (4.5 mg, 0.02 equiv., 4 μmol), [Ni(dtbbpy)$(O_2H)_4$]Cl_2 (2.8 mg, 0.03 equiv., 6 μmol), 2,6-lutidine (75.0 mg, 3.5 equiv., 0.7 mmol), *N,N*-dimethylacetamide (52.3 mg, 56 μL, 3 equiv., 0.6 mmol), sodium sulfate (426 mg, 15 equiv., 3 mmol), and 1 × 0.89 g milling ball for 3 hours. The crude product was purified by flash column chromatography (100% petroleum ether) to afford the final product, 4-benzylbenzonitrile, as a colourless oil. **^{1}H NMR (500 MHz, $CDCl_3$) δ (ppm)**: 7.61-7.58 (m, 2H), 7.37-7.25 (m, 5H), 7.21-7.17 (m, 2H), 4.06 (s, 2H). The ^{1}H NMR spectrum is consistent with the literature.[13]

*Synthesis of (**3q**).* Conducted according to the general mechanophotocatalysis and solution-state procedures. Mechanophotocatalysis procedure utilised 1-bromo-4-methoxybenzene (37.4 mg, 1 equiv., 0.2 mmol), potassium benzyltrifluoroborate (47.5 mg, 1.2 equiv., 0.24 mmol), [Ir(dF(CF3)ppy)$)_2$(dtbbpy)]PF_6 (4.5 mg, 0.02 equiv., 4 μmol), [Ni(dtbbpy)$(OH_2)_4$]Cl_2 (2.8 mg, 0.03 equiv., 6 μmol), 2,6-lutidine (75.0 mg, 3.5 equiv., 0.7 mmol), *N,N*-dimethylacetamide (52.3 mg, 56 μL, 3 equiv., 0.6 mmol), sodium sulfate (426 mg, 15 equiv., 3 mmol), and 1 × 0.89 g milling ball for 3 hours. The crude product was purified by flash column chromatography (100% petroleum ether) to afford the final product, 1-benzyl-4-methoxybenzene, as a colourless oil. **^{1}H NMR (400 MHz, $CDCl_3$) δ (ppm)**: 7.32-7.27 (m, 2H). 7.21-7.16 (m, 3H), 7.13-7.07 (m, 2H), 6.86-6.80 (m, 2H), 3.93 (s, 2H), 3.78 (s, 3H). The ^{1}H NMR spectrum is consistent with the literature.[16]

*Synthesis of (**3r**).* Conducted according to the general mechanophotocatalysis and solution-state procedures. Mechanophotocatalysis procedure utilised 1-bromo-4-(*tert*-butyl)benzene (42.6 mg, 1 equiv., 0.2 mmol), potassium benzyltrifluoroborate (47.5 mg, 1.2 equiv., 0.24 mmol), [Ir(dF(CF3)ppy)$)_2$(dtbbpy)]PF_6 (4.5 mg, 0.02 equiv., 4 μmol), [Ni(dtbbpy)$(OH_2)_4$]Cl_2 (2.8 mg, 0.03 equiv., 6 μmol), 2,6-lutidine (75.0 mg, 3.5 equiv., 0.7 mmol), *N,N*-dimethylacetamide (52.3 mg, 56 μL, 3 equiv., 0.6 mmol), sodium sulfate (426 mg, 15 equiv., 3 mmol), and 1 × 0.89 g milling

ball for 3 hours. The crude product was purified by flash column chromatography (100% petroleum ether) to afford the final product, 1-benzyl-4-(*tert*-butyl)benzene, as a colourless oil**. ^{1}H NMR (400 MHz, $CDCl_3$) δ (ppm)**: 7.35-7.27 (m, 4H), 7.25-7.18 (m, 3H), 7.18-7.11 (m, 2H), 3.97 (s, 2H), 1.32 (s, 9H). The ^{1}H NMR spectrum is consistent with the literature.[13]

Synthesis of (3s). Conducted according to the general mechanophotocatalysis and solution-state procedures. General mechanophotocatalysis procedure utilised methyl 4-bromobenzoate (64.5 mg, 1 equiv., 0.3 mmol), *tert*-butyl 4-bromopiperidine-1-carboxylate (119 mg, 1.5 equiv., 0.45 mmol), tris(trimethylsilyl)silane (74.6 mg, 93 μL, 1 equiv., 0.3 mmol), sodium carbonate (63.6 mg, 2 equiv., 0.6 mmol), [Ni(dtbbpy)(OH_2)$_4$]Cl_2 (2.8 mg, 0.02 equiv., 6 μmol), [Ir(dF(CF_3)ppy))$_2$(dtbbpy)]PF_6 (3.4 mg, 0.01 equiv., 3 μmol), sodium sulfate (767 mg, 18.0 equiv., 5.4 mmol), 1,2-dimethoxyethane (81.1 mg, 94 μL, 3 equiv., 0.9 mmol), and 1 × 0.89 g milling ball for 3 hours. Solution-state photocatalysis experiments used 1,2-dimethoxyethane (3 mL for 0.1 M) instead of sodium sulfate. Note: a higher yield was obtained for the mechanophotocatalysis reaction when using modified catalyst loadings: [Ir(dF(CF_3)ppy))$_2$(dtbbpy)]PF_6 (6.7 mg, 0.02 equiv., 6 μmol) and [Ni(dtbbpy)(OH_2)$_4$]Cl_2 (1.4 mg, 0.01 equiv., 3 μmol). The crude product was purified by flash column chromatography (0% → 6% ethyl acetate:petroleum ether) to afford the final product, *tert*-butyl 4-(4-(methoxycarbonyl)phenyl)piperidine-1-carboxylate, as an initially colourless oil that solidifies to a white solid. **^{1}H NMR (400 MHz, $CDCl_3$) δ (ppm)**: 8.00-7.94 (m, 2H), 7.29-7.23 (m, 2H), 4.25 (br s, 2H), 3.89 (s, 3H), 2.88-2.74 (m, 2H), 2.74-2.61 (m, 1H), 1.89-1.73 (m, 2H), 1.71-1.55 (m, 2H), 1.47 (s, 9H). The ^{1}H NMR spectrum is consistent with the literature.[17]

Synthesis of (3t). Conducted according to the general mechanophotocatalysis and aerobic solution-state procedures. General mechanophotocatalysis procedure utilised methyl 4-bromobenzoate (64.5 mg, 1 equiv., 0.3 mmol), ethyl 4-bromobutyrate (87.8 mg, 1.5 equiv., 0.45 mmol), tris(trimethylsilyl)silane (74.6 mg, 93 μL, 1 equiv., 0.3 mmol), sodium carbonate (63.6 mg, 2 equiv., 0.6 mmol), [Ni(dtbbpy)(OH_2)$_4$]Cl_2 (1.4 mg, 0.01 equiv., 3 μmol), [Ir(dF(CF_3)ppy))$_2$(dtbbpy)]PF_6 (6.7 mg, 0.02 equiv., 6 μmol), sodium sulfate (767 mg, 18.0 equiv., 5.4 mmol), 1,2-dimethoxyethane (81.1 mg, 94 μL, 3 equiv., 0.9 mmol), and 1 × 0.71 g milling ball for 3 hours. Solution-state photocatalysis experiments used 1,2-dimethoxyethane (3 mL for 0.1 M) instead of sodium sulfate. The crude product was purified by flash column chromatography (0% → 3% ethyl acetate:petroleum ether) to afford the final product, methyl 4-(4-ethoxy-4-oxobutyl)benzoate, as a colourless oil. **^{1}H NMR (400 MHz, $CDCl_3$) δ (ppm)**: 7.96-7.91 (m, 2H), 7.25-7.20 (m, 2H), 4.15-4.04 (m, 3H), 3.87 (s, 3H) 2.68 (t, J = 8.0 Hz, 2H), 2.29 (t, J = 7.5 Hz, 2H), 2.00-1.88 (m, 2H), 1.23 (t, J = 7.28 Hz, 2H). The ^{1}H NMR spectrum is consistent with the literature.[18]

Synthesis of (3u). Conducted according to the general mechanophotocatalysis and aerobic solution-state procedures. General mechanophotocatalysis procedure utilised methyl 4-bromobenzoate (64.5 mg, 1 equiv., 0.3 mmol), 4-bromotetrahydropyran (74.3 mg, 1.5 equiv., 0.45 mmol), tris(trimethylsilyl)silane (74.6 mg, 93 μL, 1 equiv., 0.3 mmol), sodium carbonate (63.6 mg, 2 equiv., 0.6 mmol), [Ni(dtbbpy)(OH_2)$_4$]Cl_2 (1.4 mg, 0.01 equiv., 3 μmol), [Ir(dF(CF_3)ppy))$_2$(dtbbpy)]PF_6 (6.7 mg, 0.02 equiv., 6 μmol), sodium sulfate (767 mg, 18.0 equiv., 5.4 mmol), 1,2-dimethoxyethane (81.1 mg, 94 μL, 3 equiv., 0.9 mmol), and 1 × 0.89 g milling ball for 3 hours. Solution-state photocatalysis experiments used 1,2-dimethoxyethane (3 mL for 0.1 M)

instead of sodium sulfate. The crude product was purified by flash column chromatography (0% → 5% ethyl acetate:petroleum ether) to afford the final product, methyl 4-(tetrahydro-2H-pyran-4-yl)benzoate, as a white solid. **^{1}H NMR (400 MHz, $CDCl_3$) δ (ppm)**: 8.04-7.96 (m, 2H), 7.33-7.27 (m, 2H), 4.13-4.06 (m, 2H), 3.91 (s, 3H), 3.54 (td, *J* = 11.6, 2.6 Hz, 2H), 2.90-2.75 (m, 1H), 1.92-1.73 (m, 4H). The ^{1}H NMR spectrum is consistent with the literature.[18]

Synthesis of (3v). Conducted according to the general mechanophotocatalysis and aerobic solution-state procedures. General mechanophotocatalysis procedure utilised 1-bromo-4-fluorobenzene (52.5 mg, 1 equiv., 0.3 mmol), 4-bromotetrahydropyran (74.3 mg, 1.5 equiv., 0.45 mmol), tris(trimethylsilyl)silane (74.6 mg, 93 µL, 1 equiv., 0.3 mmol), sodium carbonate (63.6 mg, 2 equiv., 0.6 mmol), $[Ni(dtbbpy)(OH_2)_4]Cl_2$ (1.4 mg, 0.01 equiv., 3 µmol), $[Ir(dF(CF_3)ppy))_2(dtbbpy)]PF_6$ (6.7 mg, 0.02 equiv., 6 µmol), sodium sulfate (767 mg, 18.0 equiv., 5.4 mmol), 1,2-dimethoxyethane (81.1 mg, 94 µL, 3 equiv., 0.9 mmol), and 1 × 0.89 g milling ball for 3 hours. Solution-state photocatalysis experiments used 1,2-dimethoxyethane (3 mL for 0.1 M) instead of sodium sulfate. The crude product was purified by flash column chromatography (0% → 3% ethyl acetate:petroleum ether) to afford the final product, 4-(4-fluorophenyl)tetrahydro-2H-pyran, as a colourless oil. **^{1}H NMR (400 MHz, $CDCl_3$) δ (ppm)**: 7.22-7.13 (m, 2H), 7.04-6.96 (m, 2H), 4.13-4.03 (m, 2H), 3.58-3.47 (m, 2H), 2.79-2.67 (m, 1H), 1.82-1.71 (m, 4H). **^{19}F NMR (377 MHz, $CDCl_3$) δ (ppm)**: -117.04 (s). The ^{1}H and ^{19}F NMR spectra are consistent with the literature.[18]

Synthesis of (3w). Conducted according to the general mechanophotocatalysis and aerobic solution-state procedures. General mechanophotocatalysis procedure utilised 4-bromoanisole (56.1 mg, 1 equiv., 0.3 mmol), 4-bromotetrahydropyran (74.3 mg, 1.5 equiv., 0.45 mmol), tris(trimethylsilyl)silane (74.6 mg, 93 µL, 1 equiv., 0.3 mmol), sodium carbonate (63.6 mg, 2 equiv., 0.6 mmol), $[Ni(dtbbpy)(OH_2)_4]Cl_2$ (1.4 mg, 0.01 equiv., 3 µmol), $[Ir(dF(CF_3)ppy))_2(dtbbpy)]PF_6$ (6.7 mg, 0.02 equiv., 6 µmol), sodium sulfate (767 mg, 18.0 equiv., 5.4 mmol), 1,2-dimethoxyethane (81.1 mg, 94 µL, 3 equiv., 0.9 mmol), and 1 × 0.89 g milling ball for 3 hours. Solution-state photocatalysis experiments used 1,2-dimethoxyethane (3 mL for 0.1 M) instead of sodium sulfate. The crude product was purified by flash column chromatography (0% → 3% ethyl acetate:petroleum ether) to afford the final product, 4-(4-methoxyphenyl)tetrahydro-2H-pyran, as a colourless oil. **^{1}H NMR (400 MHz, $CDCl_3$) δ (ppm)**: 7.17-7.12 (m, 2H), 6.90-6.83 (m, 2H), 4.10-4.04 (m, 2H), 3.80 (s, 3H), 3.57-3.47 (m, 2H), 2.77-2.65 (m, 1H), 1.85-1.70 (4H, m). The ^{1}H NMR spectrum is consistent with the literature.[18]

Synthesis of (3x). Conducted according to the general mechanophotocatalysis and aerobic solution-state procedures. General mechanophotocatalysis procedure utilised methyl 3-bromopyridine (47.4 mg, 1 equiv., 0.3 mmol), 4-bromotetrahydropyran (74.3 mg, 1.5 equiv., 0.45 mmol), tris(trimethylsilyl)silane (74.6 mg, 93 µL, 1 equiv., 0.3 mmol), sodium carbonate (63.6 mg, 2 equiv., 0.6 mmol), $[Ni(dtbbpy)(OH_2)_4]Cl_2$ (1.4 mg, 0.01 equiv., 3 µmol), $[Ir(dF(CF_3)ppy))_2(dtbbpy)]PF_6$ (6.7 mg, 0.02 equiv., 6 µmol), sodium sulfate (767 mg, 18.0 equiv., 5.4 mmol), 1,2-dimethoxyethane (81.1 mg, 94 µL, 3 equiv., 0.9 mmol), and 1 × 0.71 g milling ball for 3 hours. Solution-state photocatalysis experiments used 1,2-dimethoxyethane (3 mL for 0.1 M) instead of sodium sulfate. The crude product was purified by flash column chromatography (0% → 80% ethyl acetate:petroleum ether) to afford the final product, 3-(tetrahydro-2H-pyran-4-yl)pyridine, as a light brown oil. **^{1}H NMR (400 MHz, $CDCl_3$) δ (ppm)**: 8.50 (s, 1H), 8.49-8.42

(m, 1H), 7.59-7.49 (m, 1H), 7.26-7.21 (m, 1H), 4.16-4.03 (m, 2H), 3.54 (td, J = 11.7, 2.4 Hz, 2H), 2.87-2.71 (m, 1H), 1.88-1.74 (m, 4H). The ^{1}H NMR spectrum is consistent with the literature.[19]

*Synthesis of (**3y**).* Conducted according to the general mechanophotocatalysis and aerobic solution-state procedures. General mechanophotocatalysis procedure utilised methyl 4-bromobenzoate (64.5 mg, 1 equiv., 0.3 mmol), 1-bromoadamantane (96.8 mg, 1.5 equiv., 0.45 mmol), tris(trimethylsilyl)silane (74.6 mg, 93 µL, 1 equiv., 0.3 mmol), sodium carbonate (63.6 mg, 2 equiv., 0.6 mmol), $[Ni(dtbbpy)(OH_2)_4]Cl_2$ (1.4 mg, 0.01 equiv., 3 µmol), $[Ir(dF(CF_3)ppy))_2(dtbbpy)]PF_6$ (6.7 mg, 0.02 equiv., 6 µmol), sodium sulfate (767 mg, 18.0 equiv., 5.4 mmol), 1,2-dimethoxyethane (81.1 mg, 94 µL, 3 equiv., 0.9 mmol), and 1 × 0.89 g milling ball for 3 hours. Solution-state photocatalysis experiments used 1,2-dimethoxyethane (3 mL for 0.1 M) instead of sodium sulfate. Due to the low yield and small scale of the reaction, the product was not isolated. The following signals in the ^{1}H spectrum were used to calculate the yield, and is consistent with the literature:[18] **^{1}H NMR (400 MHz, $CDCl_3$) δ (ppm)**: 8.08-7.99 (m, 2H), 7.45-7.36 (m, 2H). The presence of the desired compound, methyl 4-(adamantan-1-yl)benzoate, was confirmed via GCMS.

*Optimization trials for **3g** and **3h***. We assessed the reaction efficiency using the relatively electron-rich substrates 4-bromotoluene and 4-bromoanisole. The results of these trials are shown in Table **S1**. Consulting the literature, electron-rich aryl bromides do not couple efficiently in many nickel-mediated aryl amination reactions, frequently returning low product yields.[20-22] Indeed, conducting solution-state reactions under the standard anaerobic conditions afforded poor yields (15 and 12% for R=Me and OMe, respectively) in 3 hours. The mechanophotocatalysis reactions (Entries 1 and 2) also gave comparably poor yields. We subsequently decided to try and optimize the mechanophotocatalysis reaction using 4-bromotoluene. Reportedly, quinuclidine as a base can help with similar substrates in nickel-catalyzed aminations,[8] but using a 0.1 mol% photocatalyst loading saw no improvement in yield (Entry 3), even over a 4-hour reaction time. Doubling the nickel catalyst loading did not increase the yield, suggesting this is not the limiting parameter of the reaction. Increasing the photocatalyst loading to 1 mol% gave only trace product formation (Entries 5 and 6). Indeed, the literature suggests trying very low photocatalyst loadings for challenging substrates.[23] Reverting back to DABCO as the base and increasing the reaction time to 4.5 hours gave a minimal increase in yield to 13% (Enty 7). Reducing the catalyst loading further to 0.017 mol% did not improve the yield (Entry 8); however, increasing the amine coupling partner loading to 5 equivalents gave a significantly improved yield of 35% (Entry 9). Increasing the amine loading to 8 equivalents did not improve the yield further (Entry 10). Further attempts to improve the reaction yield, such as increasing the nickel catalyst loading, were ineffective, giving maximum ^{1}H NMR yields of 38 and 13% for the methyl and methoxy substrates, respectively (Entries 11 and 12). 7-Methyl-1,5,7-triazabicyclo[4.4.0]dec-5-ene (MTBD) has been reported to improve yields with these challenging substrates;[23] however, this additive is relatively expensive and detracts from the appeal of the photocatalyzed amination procedure, thus we did not test the reaction with this additive.[23] Clearly electron-rich substrates remain a limitation of the current catalytic system, and a transmutation to solvent-minimized conditions does not address the underlying issues.

Table **S1.** Optimization attempts for substrates **3g** and **3h**. Using standard conditions (see **3b**) with modifications as specified.

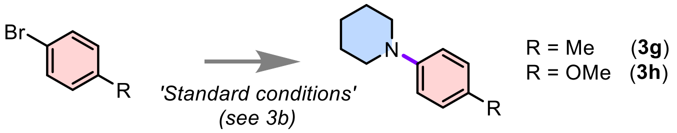

Entry	R =	Base (2 equiv.)	Amine equiv.	PC loading (mol%)	Ni loading (mol%)	Reaction time (h)	Yield (%)
[Anaerobic solution comparison DMA]	Me	DABCO	2	0.3	5	3	15
	OMe	DABCO	2	0.3	5	3	12
1	Me	DABCO	2	0.3	5	3	8
2	OMe	DABCO	2	0.3	5	3	Trace
3	Me	Quinuclidine	2	0.1	5	4	8
4	Me	Quinuclidine	2	0.1	10	4	9
5	Me	Quinuclidine	2	1	5	4	Trace
6	Me	Quinuclidine	2	1	10	4	Trace
7	Me	DABCO	2	0.1	5	4.5	13
8	Me	DABCO	2	0.017	5	4.5	13
9	Me	DABCO	5	0.017	5	4.5	35
10	Me	DABCO	8	0.017	5	4.5	35
11	Me	DABCO	8	0.017	10	4.5	38
12	OMe	DABCO	8	0.017	10	4.5	13

Yields are from quantitative ^{1}H NMR spectroscopy experiments using 1,3,5-trimethoxybenzene as an internal standard.

Additional Experimental Details

<u>Details of the Solution-State Photoreactor</u>

Solution-state photocatalysis reactions were conducted utilising a custom-built photoreactor using a 3D printed vessel holder based on a design from Leonori and co-workers, Figure **S2**.[5] The holder was then placed in a custom made photoreactor made from mirrored panels.[6] A magnetic stirrer plate allows for continuous mixing throughout the course of the reaction. Fans in a push-pull configuration limit heating of reactions by LEDs. *Kessil PR160L* LED lights were used as the excitation source, set at their maximum intensity for all experiments. The LED is approximately 7-8 cm away from the reaction vials. The enclosed nature of the reactor limits light leakage and risk to researchers.

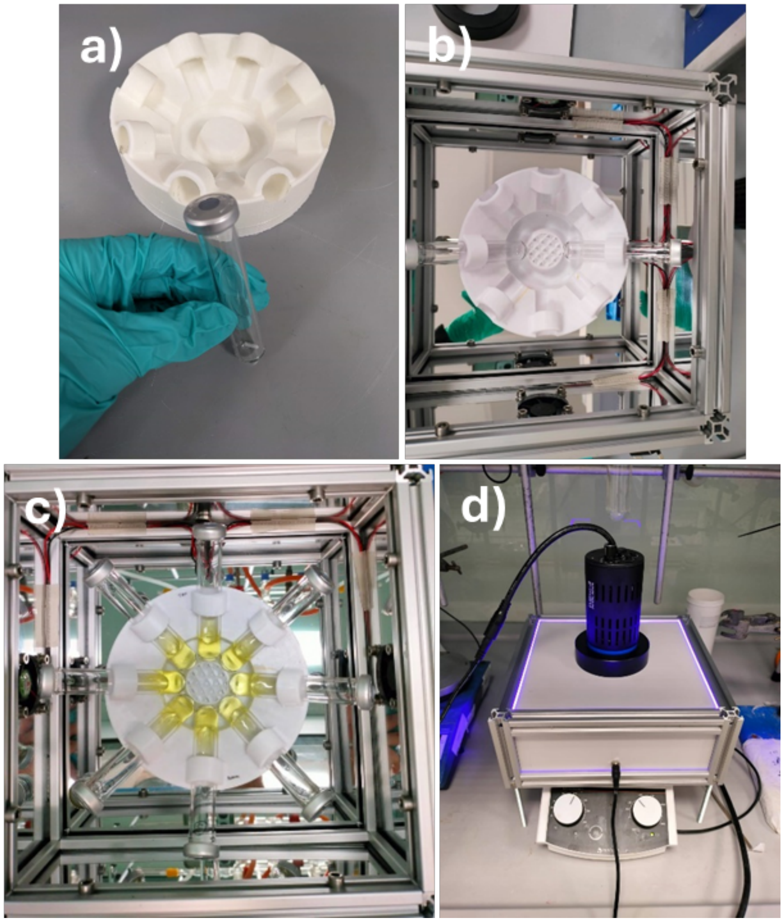

Figure **S2**. Solution-state photocatalysis reactor used in this study. a) Microwave vials with crimp lids and the 3D printed holder designed by Leonori and co-workers.[5] b) Holder is placed inside a custom photoreactor; a mirrored box with 2 fans in a push pull configuration. c) Up to 8 reactions can be conducted inside the reactor simultaneously. d) The outside of the reactor with *Kessil* LED and stirrer plate.

Details of the Mechanophotocatalysis Reactor

The mechanophotocatalysis reactor used in this study is shown in Figure **S3**. A commercially available *Retsch MM400* shaker ball mill was modified with a custom stainless steel safety shield that minimises undesired light leakage. The interior surface is reflective. A hole in the milling shield above each jar holder allows for the irradiation of the reaction from above from a *Kessil PR160L* LEDs, set at its maximum intensity setting for all experiments. The LED is approximately 8-10 cm away from the milling vessels during milling. A mirror on the base of the mill underneath the milling vessels reflects light from the LED to the underside of the milling vessels. A divider attached to the mill shield separates each side of the mill, so that only light from one LED illuminates each milling vessel, providing a fair comparison with the solution-state reactions, and allowing reactions using different light sources to be conducted simultaneously on different sides of the reactor.

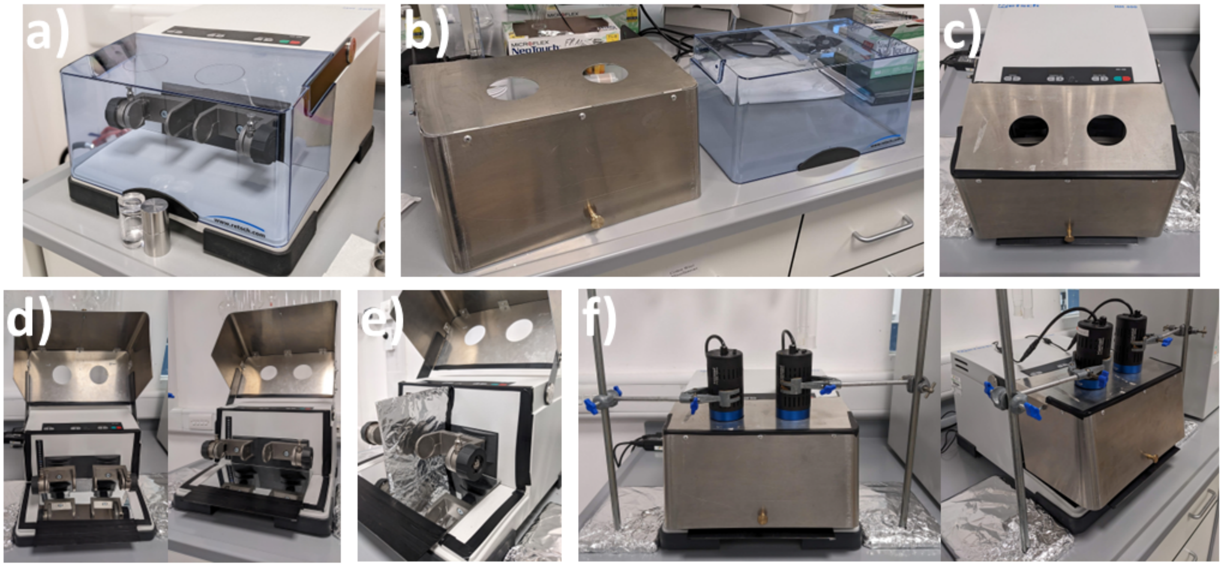

Figure **S3**. Prototype mechanophotocatalysis reactor. a) Commercially available *Retsch MM400* ball mill with its original plastic safety shield. b) Custom stainless steel safety shield (left) next to original plastic safety shield (right). c) Safety shield has a cutout above each jar holder. d) Mirror on base of the mill. e) Dividing reflective insert separating the reactor into two distinct sections irradiated by one light each. f) Irradiation of reactions during milling is achieved using a *Kessil* LED above each hole in the safety shield, held in position using a clamp stand. Figure adapted from our previous report.[4]

Details of the Mechanophotocatalysis Reaction Vessels

Our previous report on mechanophotocatalysis used small 2 mL glass vials as reaction vessels, which were incased in a poly(methyl methacrylate) (PMMA) milling jar to allow for secure clamping in a ball mill.[4] While this approach works, we recognized the potential for improving this methodology. This work pioneers the use of 2 mL polypropylene Eppendorfs as transparent, chemically resistant, cheap reaction vessels for conducting small scale solvent-minimized photochemistry within a ball mill. The Eppendorfs used in this work were obtained from Sigma Aldrich (product code Z628034). Initially we tested their viability as reaction vessels by again encasing them in the PMMA shells previously used; we refer to this as the 'V1 Eppendorf holder'. Subsequently, we designed a new holder that could replace the PMMA encapsulation approach. The 'V2 Eppendorf holder' is made from 3 machined metal (aluminum) disks, 2 rods, and 6 screws, and was made by our university's workshop (design specifications provided). This is the most accessible methodology in the field thus far for attempting light driven reactions using milling. 316-grade stainless steel ball bearings were obtained from https://simplybearings.co.uk.

Some important considerations are advised for other researchers conducting work with the Eppendorfs and the V2 holder. We have tested a maximum milling frequency of 30 Hz (using a MM400 mixer mill), and the maximum ball sized used was 1 × 6 mm ball (0.89 g). Under these conditions, over a 2-3 hour reaction, the lids of the Eppendorfs break and material deposits onto the top lid disk of the holder. Additionally, if the Eppendorf is not filled sufficiently (at least 1/8 full), larger balls or higher milling speeds also lead to damage and potential loss of material. We found that a milling speed of 25 Hz and the use of additional grinding auxiliary to bulk out the reaction mixture prevents damage of the Eppendorfs, even over extended reaction times of 4.5 hours. The Eppendorfs are optimal for 0.1-0.3 mmol scale reactions; above this they become filled past 50% filling volume; the balls do not mix the reagents satisfactorily and too much light is blocked by the caking of material to the walls of the Eppendorfs. We would strongly recommend the use of smaller milling balls (for example, a 1 × 0.71 g ball) for reactions at smaller scales and filling volumes, or for reactions with a high liquid content, or for reactions requiring extended milling times, in order to limit the potential for leakages (for example, the halogen atom transfer reaction benefits from this modification). Using the smaller 0.71 g ball, no leaks have yet been found, over multiple different reactions at 25 Hz over 4.5-hour time periods. In general, we suggest the use of one larger ball rather than multiple smaller balls, as these frequently become immobilized in 'sticky' reaction mixtures, leading to poor milling efficiency.

In some reactions, gas evolved. If not planned for, a buildup of gas can cause the Eppendorf lid to suddenly 'pop' open once released from the V2 holder. This can be prevented by rapidly piercing the lid of the Eppendorf with a bleed needle as soon as it has been released from the holder. The decarboxylative arylations and the XAT cross-coupling both required this venting, while the aryl amination and deborylative arylations produced no gas. The evolution of CO_2 from the decarboxylative arylation was expected, and thus this reaction was limited to a 0.1 mmol scale for this proof-of-concept.

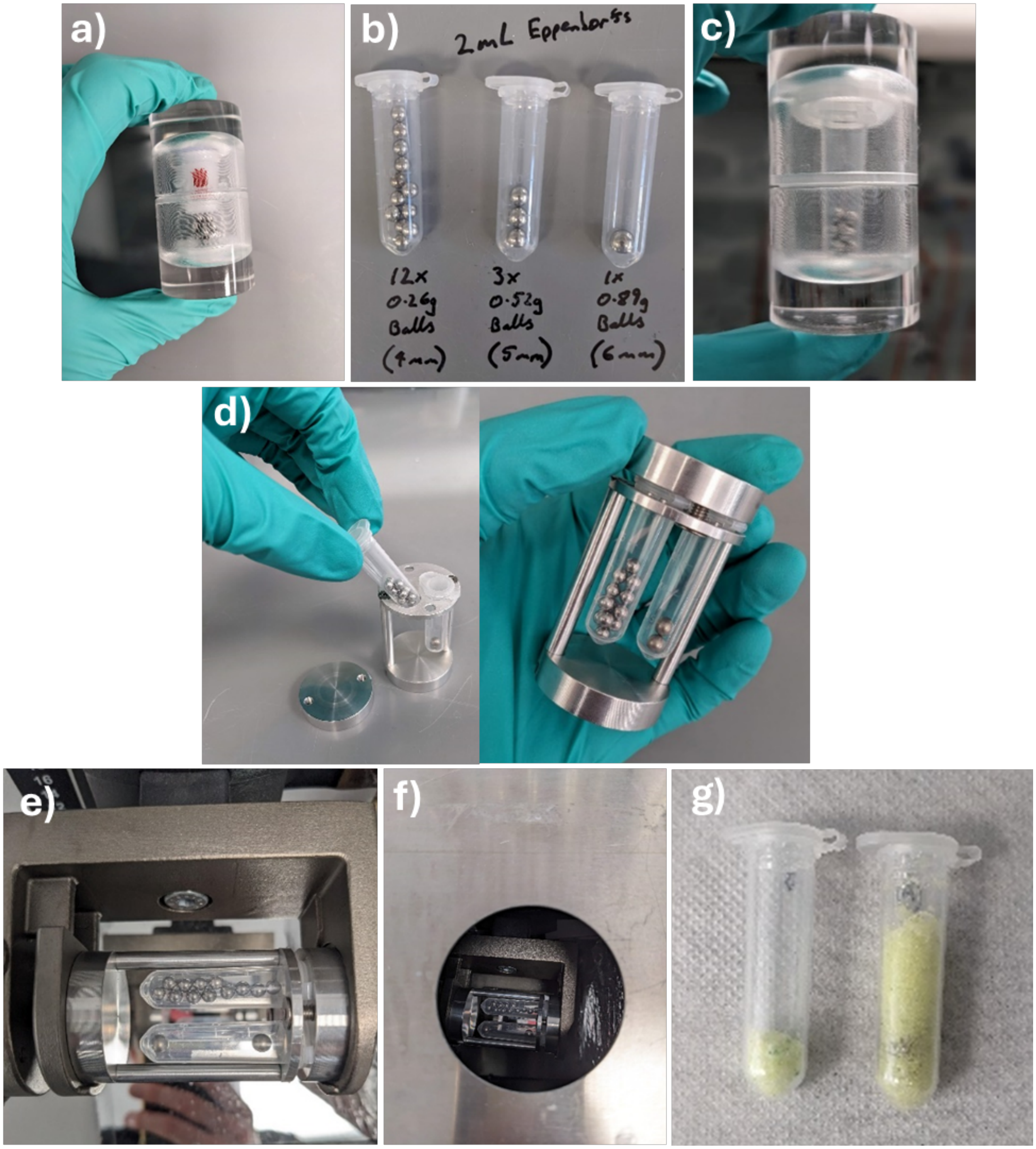

Figure **S4**. Mechanophotocatalysis reaction vessels. a) Our previous reaction vessels;[4] 2 mL glass vials (within which the reaction takes place) held inside a transparent PMMA capsule (allowing for secure clamping within the mill). b) 2 mL transparent Eppendorfs used in this study, containing different milling balls; either 12 × 4 mm diameter balls (0.26 g each), 3 × 5 mm diameter balls (0.52 g each), or 1 × 6 mm diameter balls (0.89 g each). c) The 'V1 holder'; encasing the reaction vessel Eppendorf inside the PMMA jar for secure clamping in the mill. d) The 'V2 holder'; allows the secure clamping of the Eppendorfs without the PMMA jar, and allows for increased screening

of reactions. e) The V2 holder clamped in the mill. f) View of V2 holder clamped in mill through the hole in the mill cover. g) The Eppendorfs containing the starting reaction mixture for the aryl amination for the synthesis of product **3a**. The vial on the left is a 0.1 mmol scale reaction and the vial is approximately 1/8 full. The vial on the right is at a 0.3 mmol scale and is approximately 1/2 full.

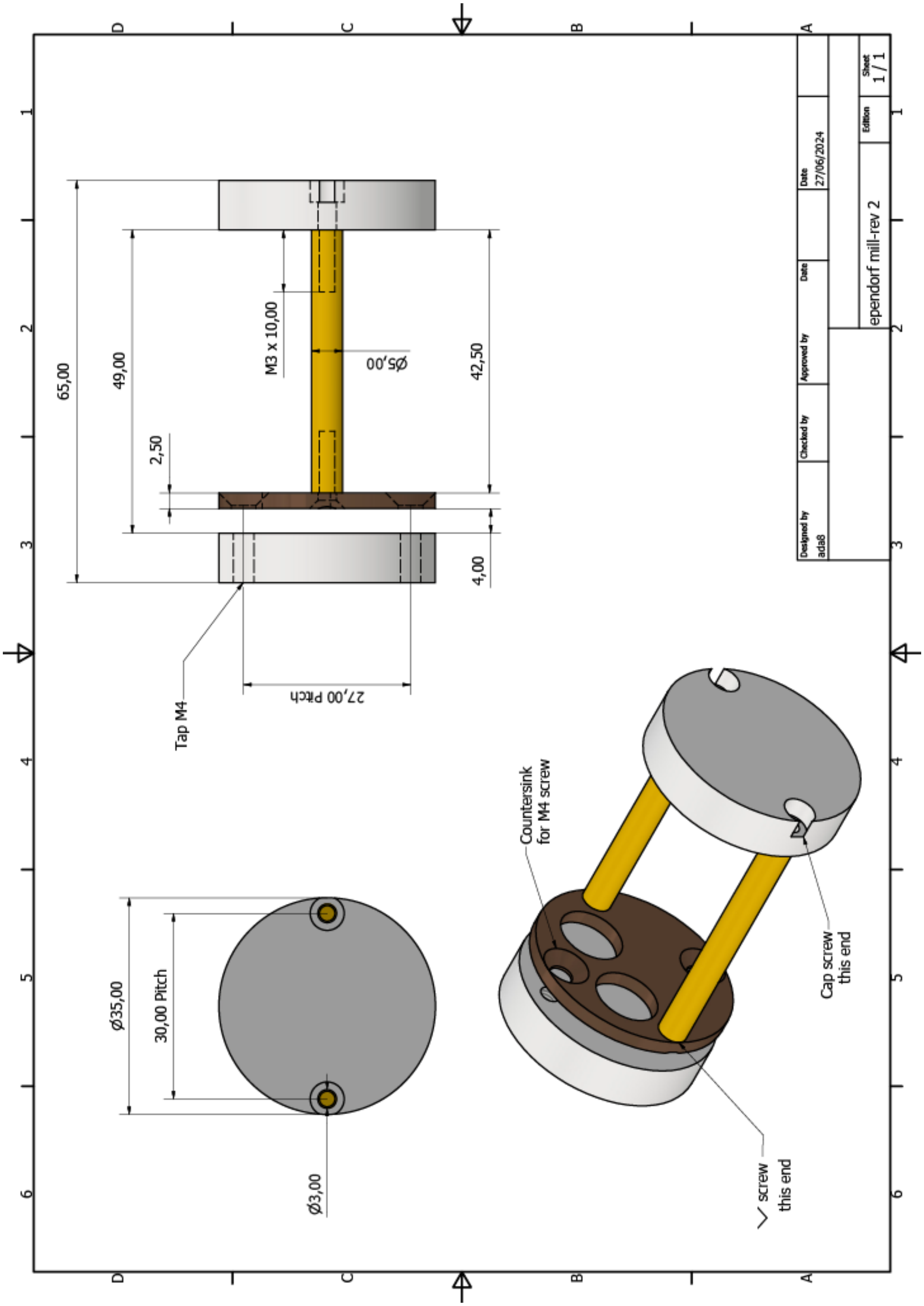

Figure **S5**. V2 Eppendorf holder design schematics.

Mechanophotocatalysis Reactor Temperature Control Studies

Initial temperature tests were conducted using an *HYGIPLAS* infrared temperature sensor. Black non-reflective tape was attached to the surface of reaction vessel to ensure that accurate readings were taken. Following 2-hour mechanophotocatalysis reactions, the temperature of the reaction vial can be measured to be between 40-50 ˚C. The global temperature underneath the mill shield was found to climb towards 38-40 ˚C (also confirmed using colour changing temperature strips). At present, it is unclear to what extent the thermal heating during mechanophotocatalysis contributes to the reactivity observed. This elevated temperature may prove advantageous for certain photocatalysis reactions, and there are examples of solution-state photocatalysis reactions that are conducted at elevated temperatures.[24] However, in other photocatalysis reactions the mechanochemical thermal contribution may limit control over selectivity and may limit substrate compatibility. As with all new synthetic protocols, the boundaries of these applications need to be considered.

'No light' control experiments were conducted, where reaction vessels were wrapped in foil to maintain darkness, while the external lights were still employed to maintain identical temperatures to the standard conditions.

Photoluminescence studies

Photophysical studies were conducted using an FS5 Spectrofluorometer from Edinburgh Instruments. Solution-state measurements were conducted at optically dilute concentrations on the order of 10^{-5} M for **[Ir1], 4CzIPN, [Ir2]**, and **[Ru1],** or 10^{-4} M for **[Cu1]** in spectroscopic or HPLC-grade solvents using a standard cuvette fitted with a rubber septum. The SC-05 cuvette holder module from Edinburgh Instruments was used. Degassed (anaerobic) solution-state samples were obtained via bubbling the solution inside the cuvette with solvent saturated nitrogen for at least 30 minutes. Aerated solutions were obtained from non-degassed stock solutions (if the original solvent was a non-degassed solvent bottle), or by bubbling the degassed solution inside the cuvette with compressed air for 1 minute. Solid (powder) samples were measured inside a Young's J NMR tube using the SC-70 NMR tube holder (Dewar) from Edinburgh Instruments. Aerobic samples were measured first. Degassed (anaerobic) solid samples were then obtained via 3 vacuum/nitrogen cycles and left under vacuum. Steady-state photoluminescence measurements (SS PL) were excited at λ_{exc} of 380 nm under anaerobic conditions. Time-resolved PL measurements (TR PL) were conducted using an EPL laser excitation source from Edinburgh Instruments (λ_{exc} = 375 nm) using TCSPC or MCS. The lifetime data are reported in Table **S2**.

Initial qualitative quenching studies. The original aerobic quenching studies for the solid powder samples were conducted in Young's J NMR tubes, Figure **S6a-b**. However, noting the transparent nature of the Eppendorfs we were using as reaction vessels, we questioned whether these could be used for photoluminescence measurements to rapidly analyze reaction mixtures, Figure **S6c-d**. The time-resolved PL spectra of **[Ir1]** in the NMR tube and in the Eppendorf overlay, and there is no time resolved PL signal from the empty NMR tube, or Eppendorf (empty or containing sodium sulfate), Figure **S6e**. Using the Eppendorf, the steady-state and time-resolved PL of **[Ir1]** as a neat solid, and as a mixture of **[Ir1]** (3.4 mg) in sodium sulfate (767 mg) before and after milling 25 Hz with 1 x 0.52 g ball for 15 minutes, were measured, **S6f-g**. The milled sample retained the emission onset of neat **[Ir1]**, but the PL spectrum broadened. Similarly, a small decrease in the lifetime was observed. These photophysical changes were initially considered to be a result of the sensitivity to environmental changes of the CT excited state of **[Ir1]**; however, 3 minor new signals in the ^{19}F NMR spectrum suggest that a small portion of **[Ir1]** potentially underwent a chemical change during the milling process, Figure **S6h**, conjectured to be a partial salt exchange between the PF_6^- and SO_4^{2-} counter anions. The dominant species present remains **[Ir1]**, and thus as a proof-of-concept to qualitatively observe quenching by substrates, **[Ir1]** (3.4 mg), sodium sulfate (767 mg) were milled together (25 Hz with 1 x 0.52 g ball for 15 minutes), and the time-resolved PL-decay was measured. This was then repeated in the presence of different substrates at loadings similar to those used in the reactions explored in this study. We began with DABCO (67.3 mg), which induced significant quenching of the excited state, Figure **S6i**. The ^{19}F NMR spectrum of the quenched sample suggested no structural changes to the **[Ir1]** core, Figure **S6h** (purple), showed that the observed quenching indeed arose from a bimolecular electronic interaction, rather than a chemical modification. Considering the excited-state redox potentials of **[Ir1]** (E^*_{ox} = -1.05 V, E^*_{red} = 1.37 V vs SCE in MeCN)[25] and the oxidation potential of DABCO (0.71 V vs SCE in DMF)[26], such quenching is expected to occur via oxidation of the amine by **[Ir1]** in its excited state.[26] Quenching with DABCO was so efficient that shining a torch on the sample led to dramatically reduced emission brightness, Figure **S6j**. Significant quenching was also observed after milling with **[Ni1]** (2.8 mg), Figure **S6i**. Based on the reduction potential of

[Ni1] (-1.36 V vs SCE for Ni(bpy)Cl_2 in DMF),[27] and mechanistic studies,[28] the quenching mechanism of **[Ni1]** in the absence of additional substrates likely occurs via photoinduced energy transfer. Minimal quenching was observed when milling with potassium benzyltrifluoroborate (**2f**, 119 mg), and no quenching was observed when milling with methyl-4 bromobenzoate (**1a**, 129 mg), Figure **S6k**. Despite the oxidation potential of potassium benzyltrifluoroborate suggesting that single electron transfer is thermodynamically feasible (1.05 V vs SCE in MeCN),[29, 30] there is precedent that this reaction may occur via transmetallation of the BF_3K salt with the nickel catalyst.[28] Furthermore, due to the high reduction potential of methyl 4-bromobenzoate (-1.88 V vs SCE in MeCN),[31] quenching would not be expected. Interestingly, we observed an increase in the excited state lifetime upon milling with methyl 4-bromobenzoate; the origin of this remains perplexing. Efforts are underway in our laboratory to develop a quantitative methodology based on these initial results in order to study mechanophotocatalysis reaction mechanisms.

Table **S2.** Lifetimes of different photocatalysts under aerobic and anaerobic conditions in solution and as neat solids. Lifetimes reported to 2 significant figures with the χ^2 reported in [brackets].

	**[Ir1]**	**[Ru1]**	**[Ir2]**	**4CzIPN**	**[Cu1]**
Anaerobic Solution-state τ_{PL} / ns	1.8×10^3 [1.1] [1]<br>2.1×10^3 [1.0] [2]	8.6×10^2 [1.0] [3]	1.8×10^3 [1.1] [3]	3.0×10^1,<br>2.3×10^3 [1.2] [1]	2.1×10^2 [1.4] [3]
Aerobic Solution-state τ_{PL} / ns	1.9×10^2 [1.3] [1]<br>2.0×10^2 [1.4] [2]	1.4×10^2 [1.2] [3]	1.9×10^1 [1.5] [3]	2.3×10^1,<br>7.9×10^2 [1.2] [1]	5.7×10^1 [1.2] [3]
Solution-state Aerobic Quenching Factor	≈ 9.5 [1]<br>≈ 10.5[2]	≈ 6.1 [3]	≈ 95 [3]	≈ 1.3//2.9 [1]	≈ 3.7 [3]
Anaerobic Solid-state τ_{PL} / ns	1.1×10^3 [3.9] [4]	1.2×10^3 [1.5] [4]	2.6×10^2 [3.2] [4]	3.6×10^2,<br>1.9×10^3 [1.1] [4]	2.3×10^4 [1.2] [4]
Aerobic Solid-state τ_{PL} / ns	7.1×10^2 [3.5] [4]	1.2×10^3 [1.5] [4]	2.7×10^2 [3.1] [4]	1.3×10^2,<br>1.6×10^3 [1.2] [4]	2.3×10^4 [1.8] [4]
Solid-state Aerobic Quenching Factor	≈ 1.55 [4]	≈ 1 [4]	≈ 1 [4]	≈ 2.8//1.2 [4]	≈ 1 [4]

[1] Measured in DMA. [2] Measured in DME. [3] Measured in MeCN. [4] Measured as a powder.

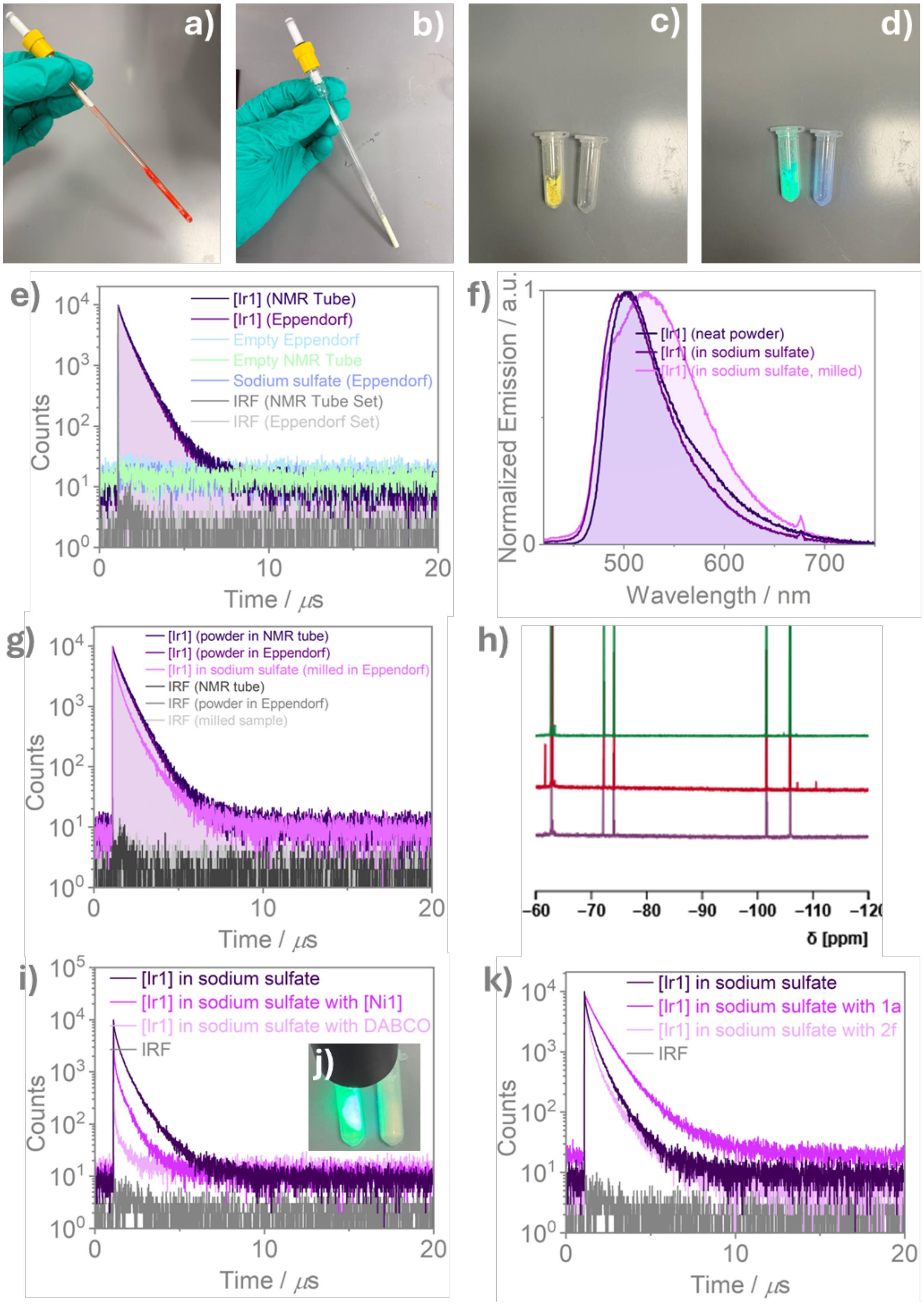

Figure **S6**. Quenching studies. a) Original vessel for measuring the photophysical properties of solid samples: Young's J NMR tube containing **[Ru1]**. b) Young's J NMR tube containing **[Cu1]**.

c) Empty Eppendorf (right) and Eppendorf containing **[Ir1]** (left). d) Empty Eppendorf (right) and Eppendorf containing **[Ir1]** under irradiation by UV torch. e) Time-resolved PL decay of **[Ir1]** in the NMR tube and Eppendorf, and background comparison from the empty vessels. f) Steady state PL of **[Ir1]** measured in an Eppendorf as a neat powder, as a lightly shaken sample in sodium sulfate, and as a milled sample in sodium sulfate. g) Time-resolved PL decay of **[Ir1]** measured in an Eppendorf as a neat powder, and as a milled sample in sodium sulfate. h) ^{19}F NMR of neat **[Ir1]** (green) , and of **[Ir1]** after milling in sodium sulfate for 15 minutes at 25 Hz with 1 × 0.52 g milling ball (red), and with DABCO (purple). i) Time-resolved PL decay of **[Ir1]** measured in an Eppendorf as a milled sample in sodium sulfate, with quenching by DABCO and **[Ni1]**. j) **[Ir1]** in sodium sulfate (left) and with DABCO (right) under UV torch irradiation. k) Time-resolved PL decay of **[Ir1]** measured in an Eppendorf as a milled sample in sodium sulfate, with limited/no quenching by **2f** and **1a**, respectively.

NMR Spectra

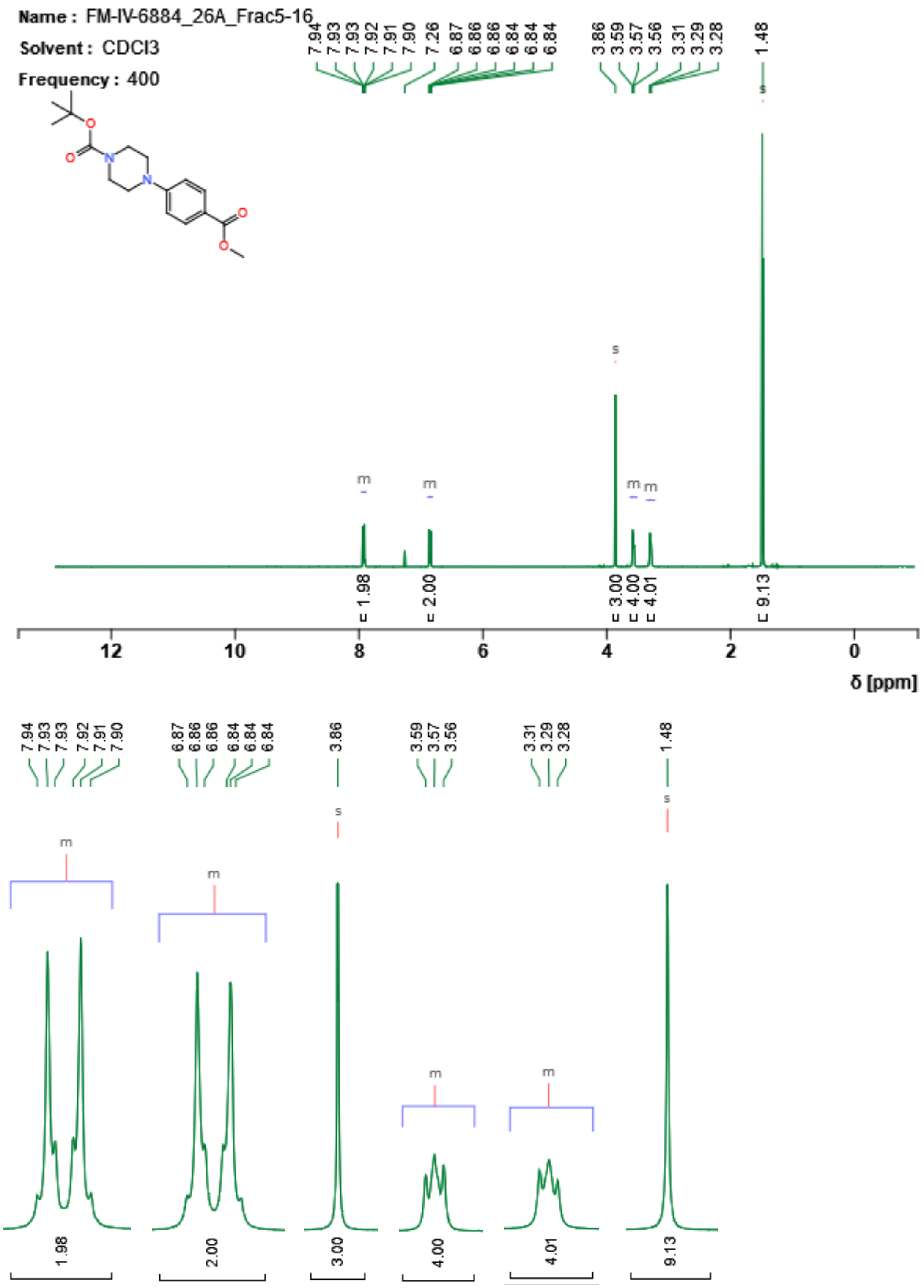

Figure **S7**. ^{1}H NMR spectrum of tert-butyl 4-(4-(methoxycarbonyl)phenyl)piperazine-1-carboxylate (**3a**) in $CDCl_3$.

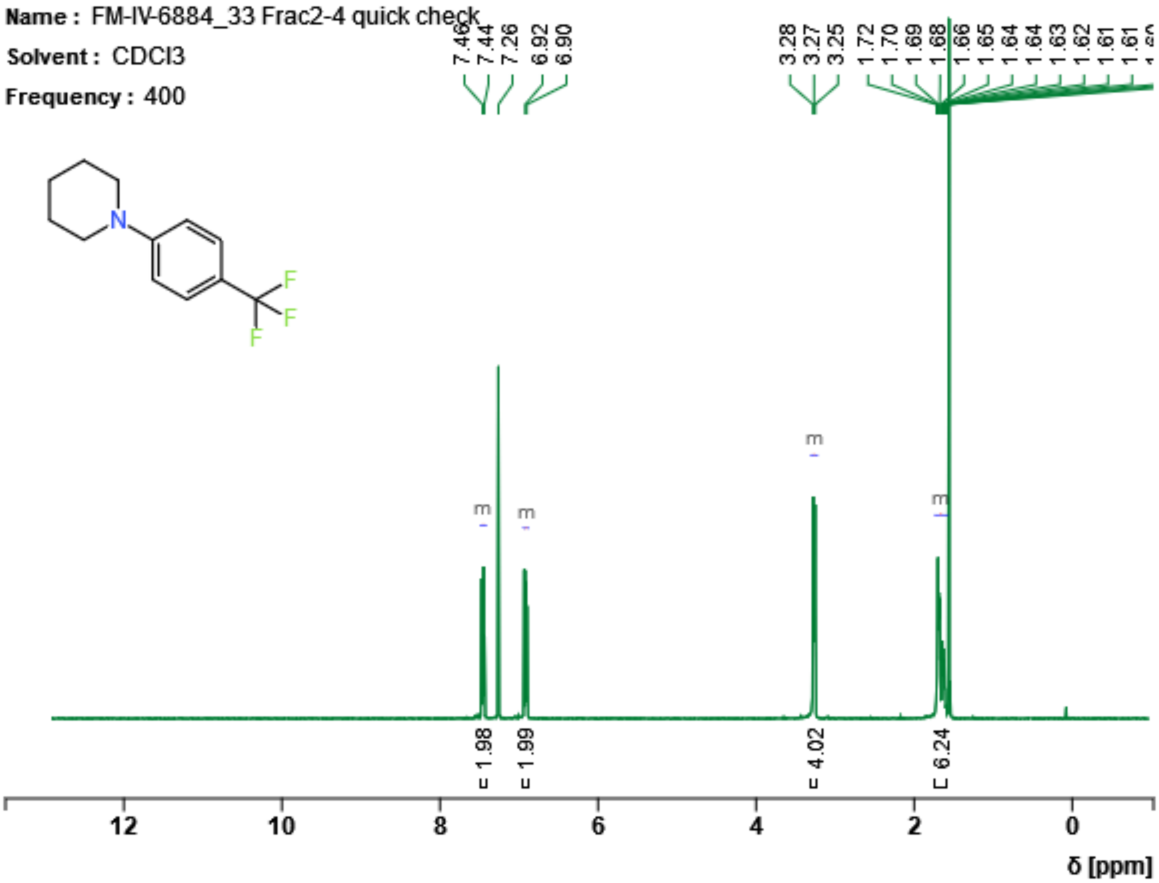

Figure **S8**. ^{1}H NMR spectrum of 1-(4-(trifluoromethyl)phenyl)piperidine (**3b**) in $CDCl_3$.

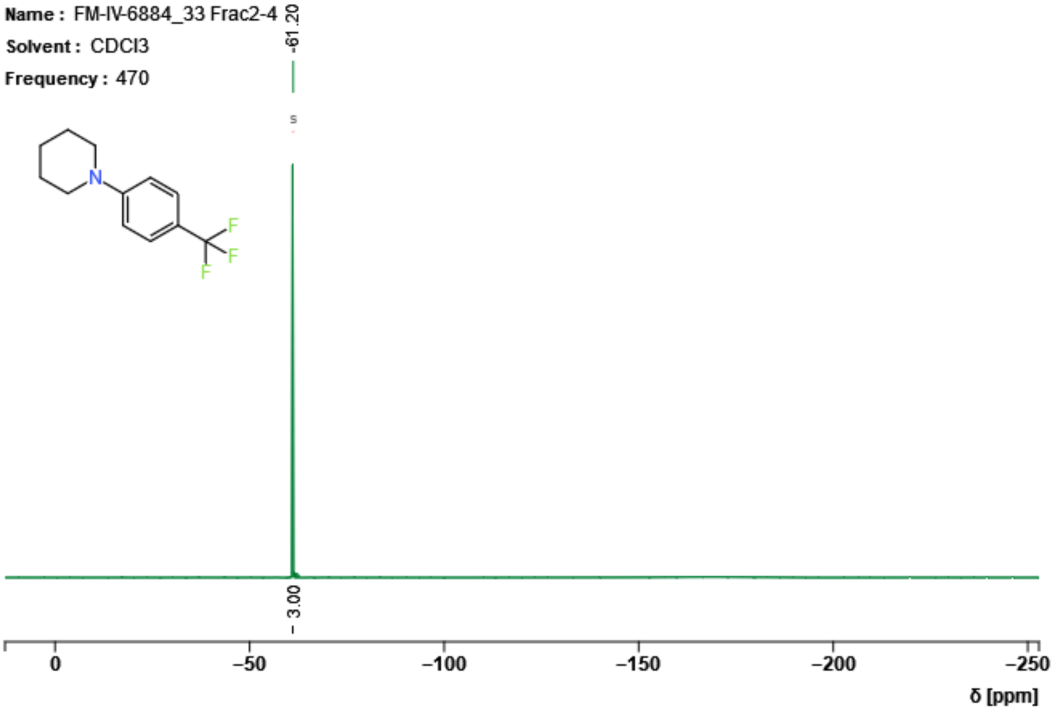

Figure **S9**. ^{19}F NMR spectrum of 1-(4-(trifluoromethyl)phenyl)piperidine (**3b**) in $CDCl_3$.

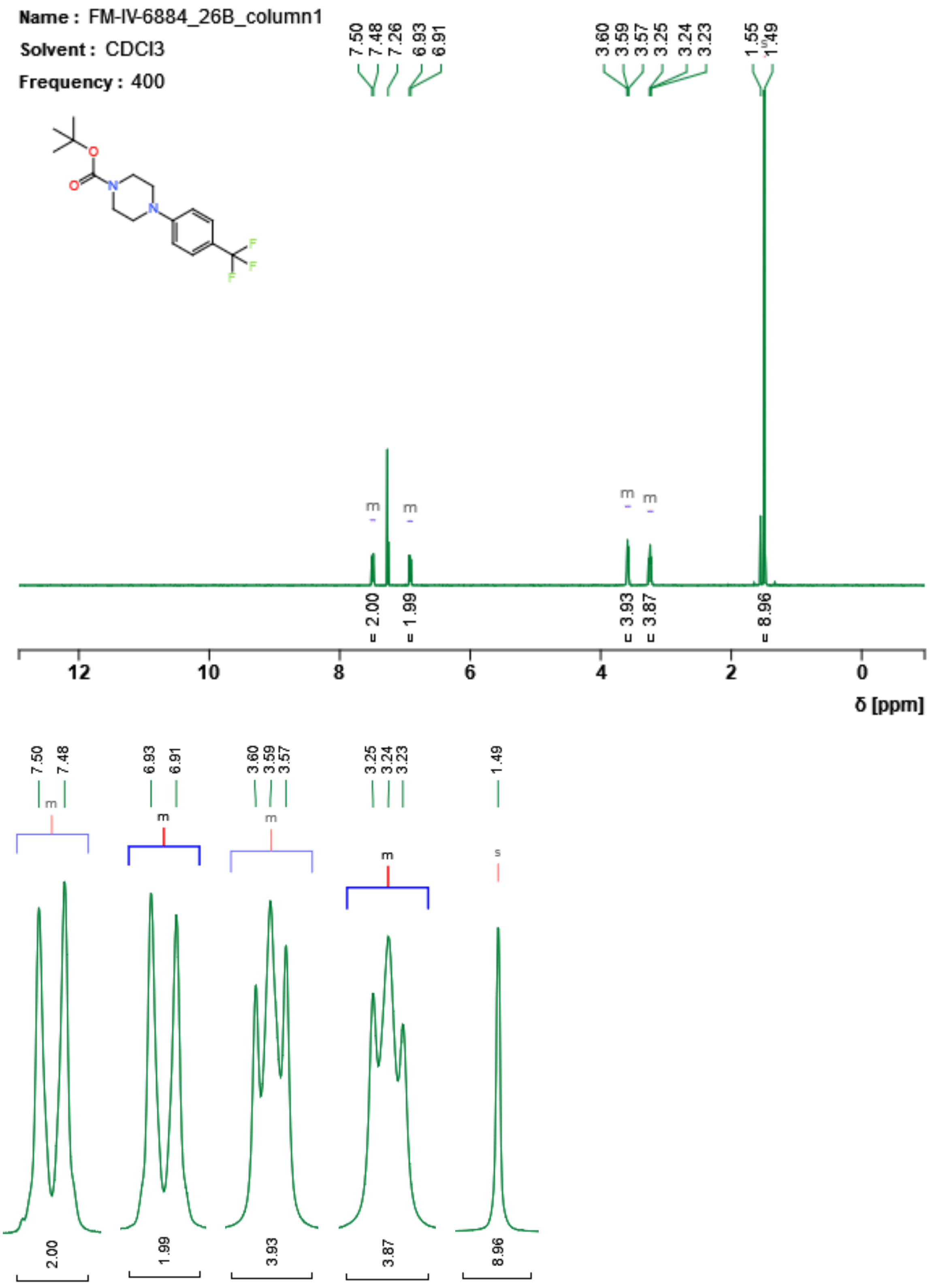

Figure **S10**. ^{1}H NMR spectrum of *tert*-butyl 4-(4-(trifluoromethyl)phenyl)piperazine-1-carboxylate (**3c**) in $CDCl_3$.

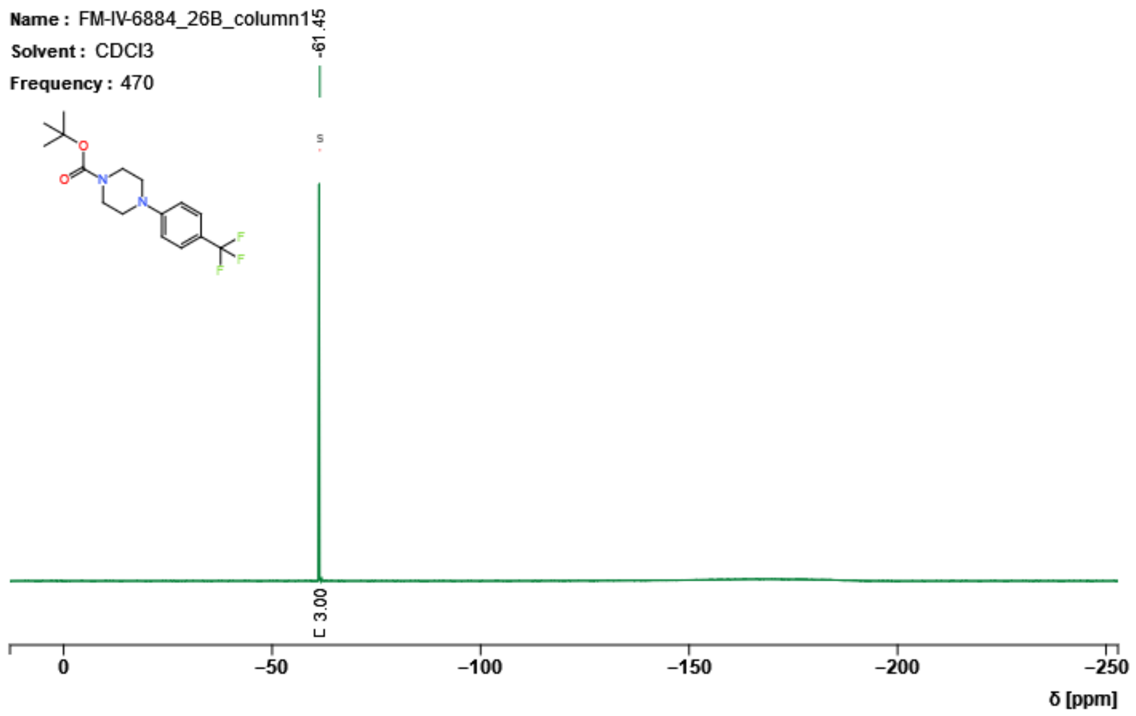

Figure **S11**. ^{19}F NMR spectrum of *tert*-butyl 4-(4-(trifluoromethyl)phenyl)piperazine-1-carboxylate (**3c**) in $CDCl_3$.

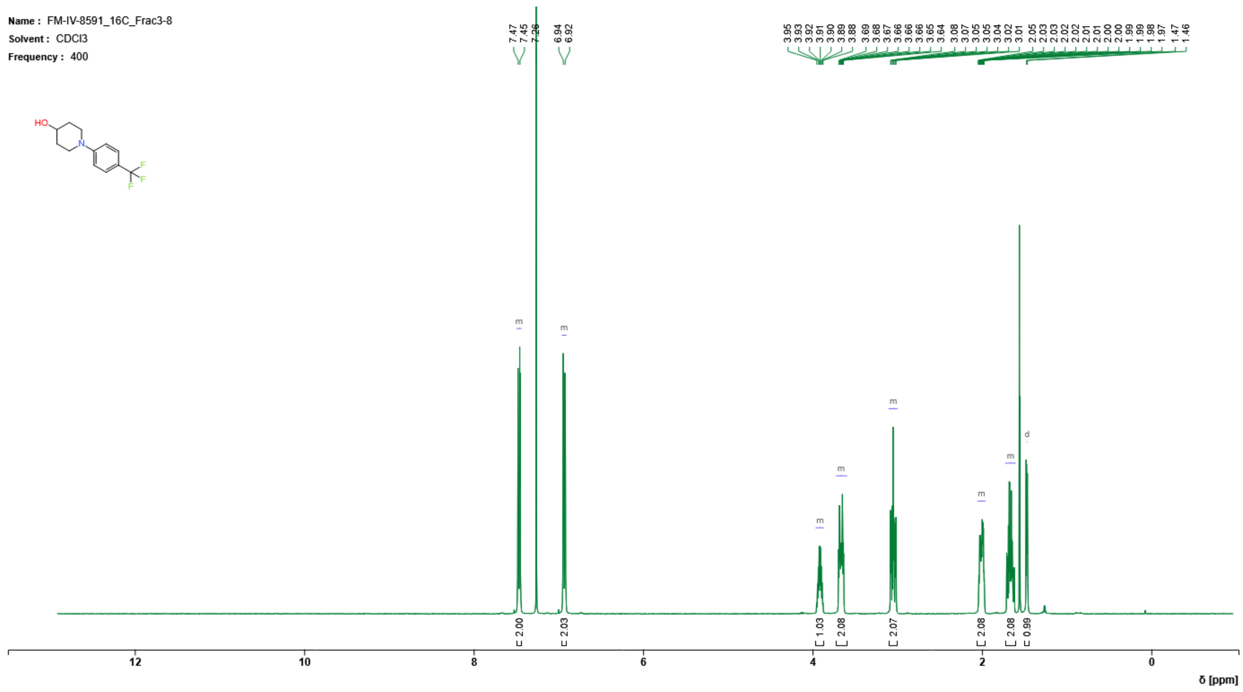

Figure **S12**. ^{1}H NMR spectrum of 1-(4-(trifluoromethyl)phenyl)piperidin-4-ol (**3d**) in $CDCl_3$.

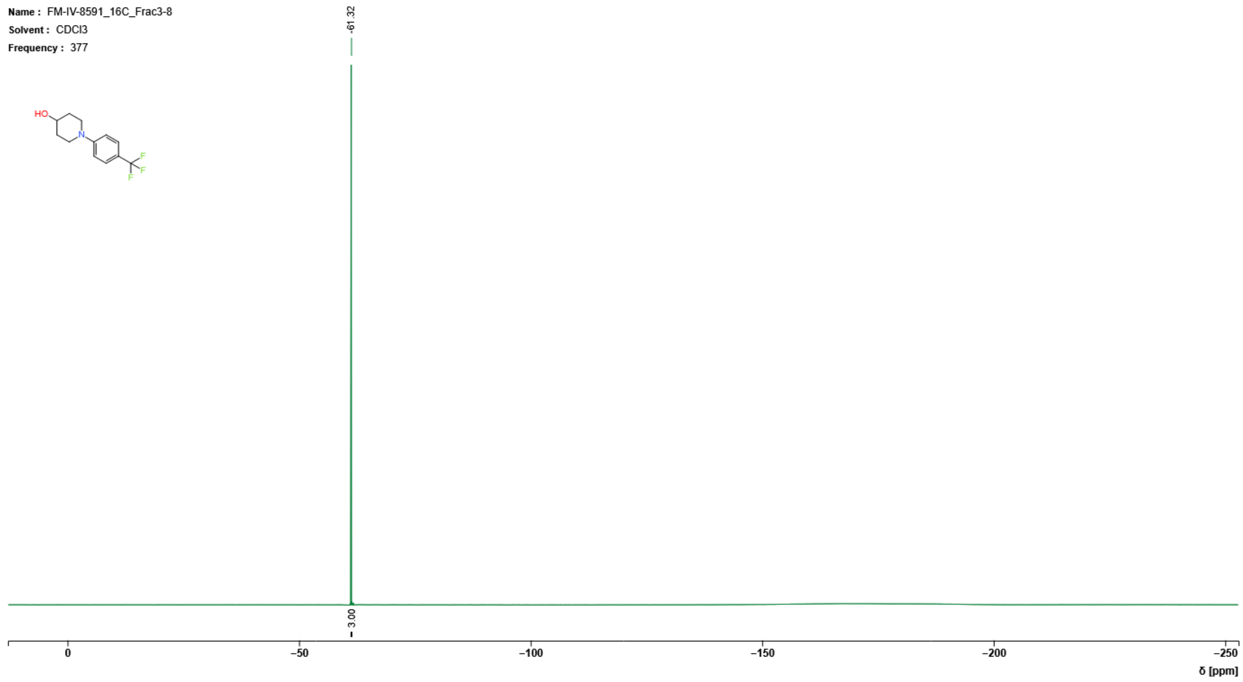

Figure **S13**. ^{19}F NMR spectrum of 1-(4-(trifluoromethyl)phenyl)piperidin-4-ol (**3d**) in $CDCl_3$.

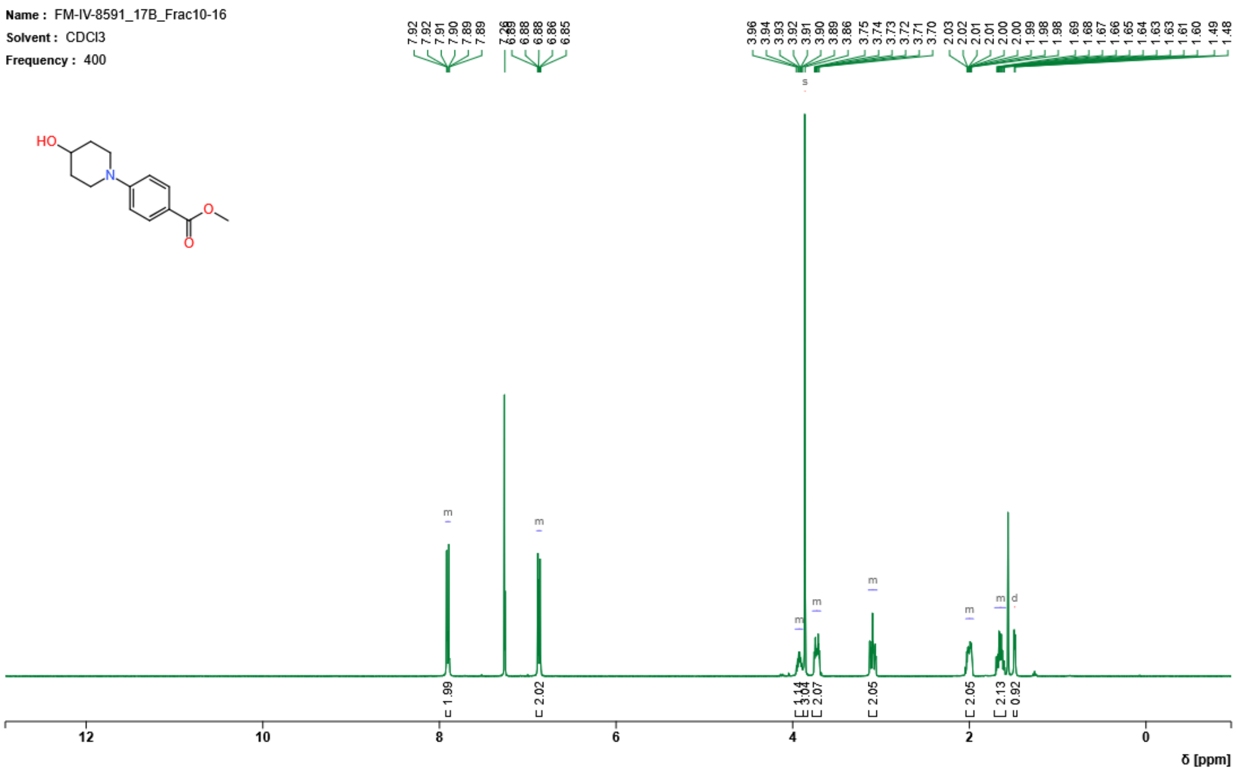

Figure **S14**. ^{1}H NMR spectrum of methyl 4-(4-hydroxypiperidin-1-yl)benzoate (**3e**) in $CDCl_3$.

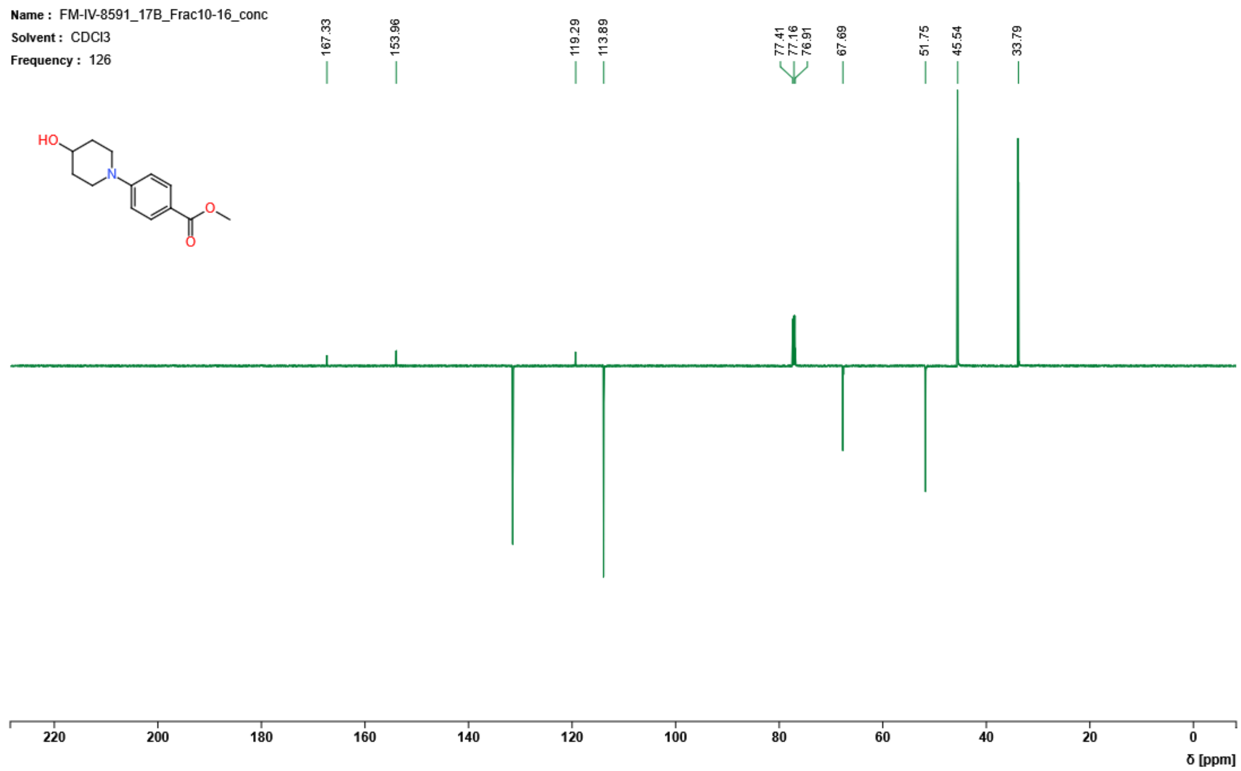

Figure **S15**. ^{13}C NMR spectrum of methyl 4-(4-hydroxypiperidin-1-yl)benzoate (**3e**) in $CDCl_3$.

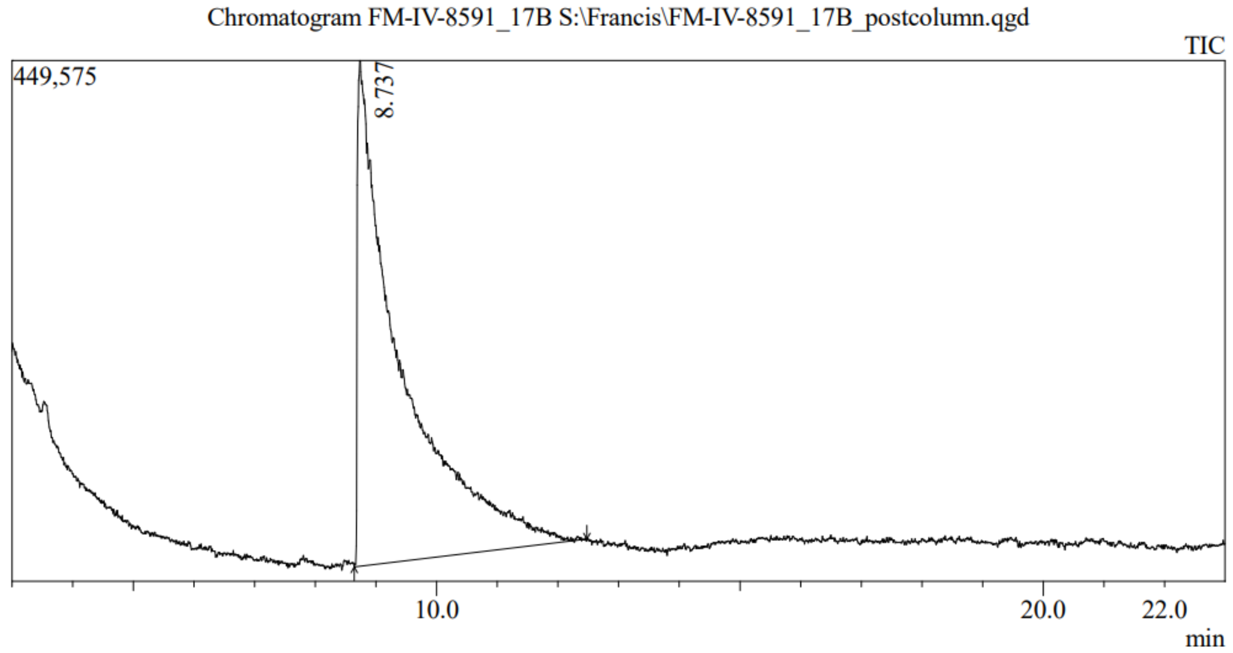

Peak Report TIC

Peak#	R.Time	Area	Area%	Height	A/H	Base m/z	Base Int.
1	8.737	21484559	100.00	436512	49.22	235.15	51801
		21484559	100.00	436512			

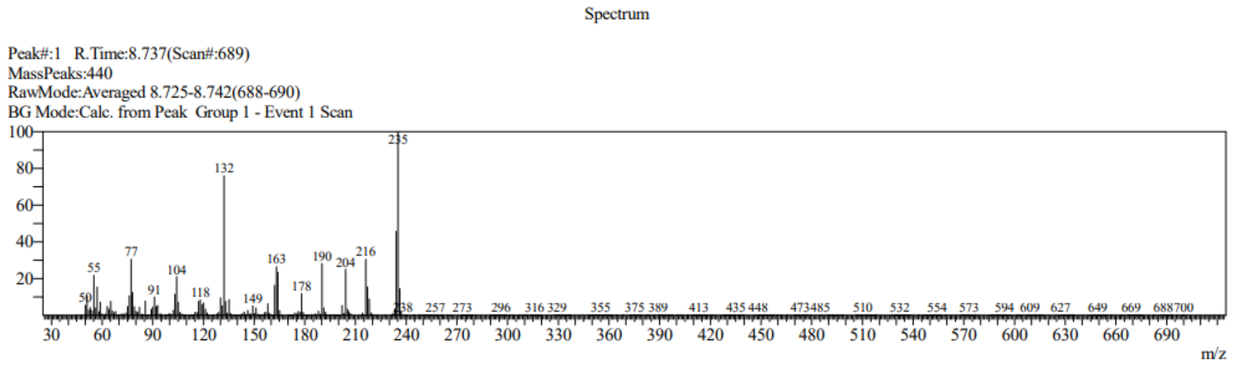

Figure **S16**. GCMS of methyl 4-(4-hydroxypiperidin-1-yl)benzoate (**3e**).

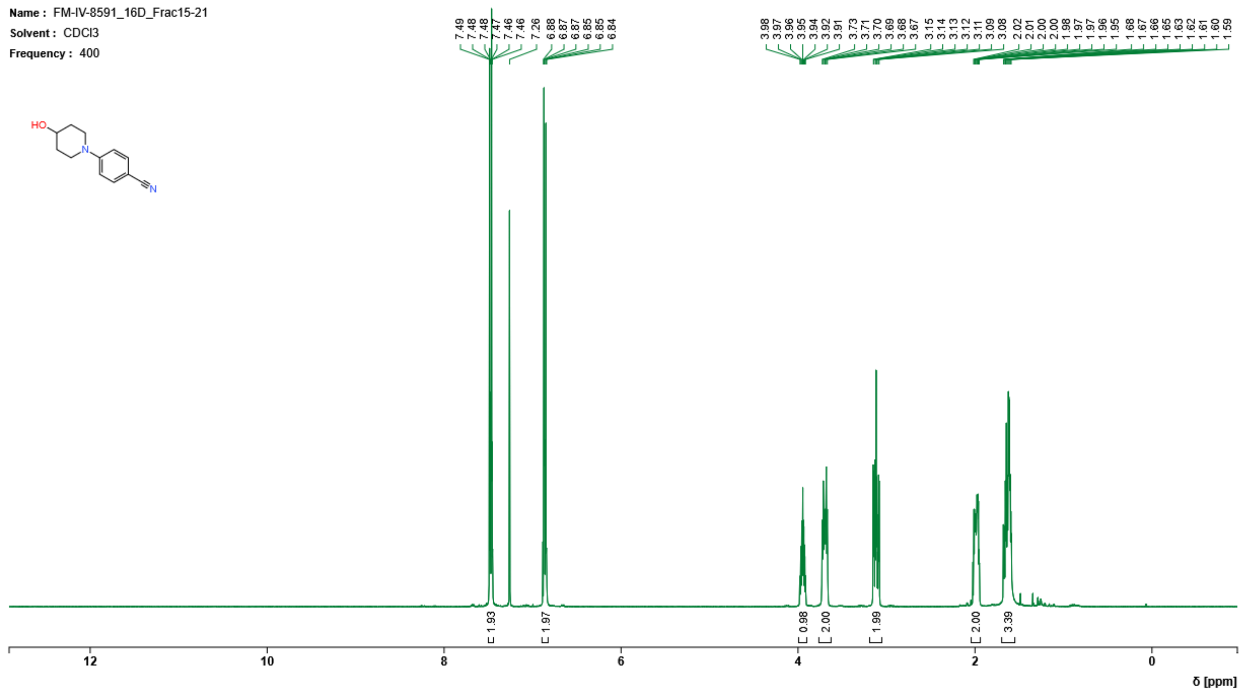

Figure **S17**. ^{1}H NMR spectrum of 4-(4-hydroxypiperidin-1-yl)benzonitrile (**3f**) in $CDCl_3$.

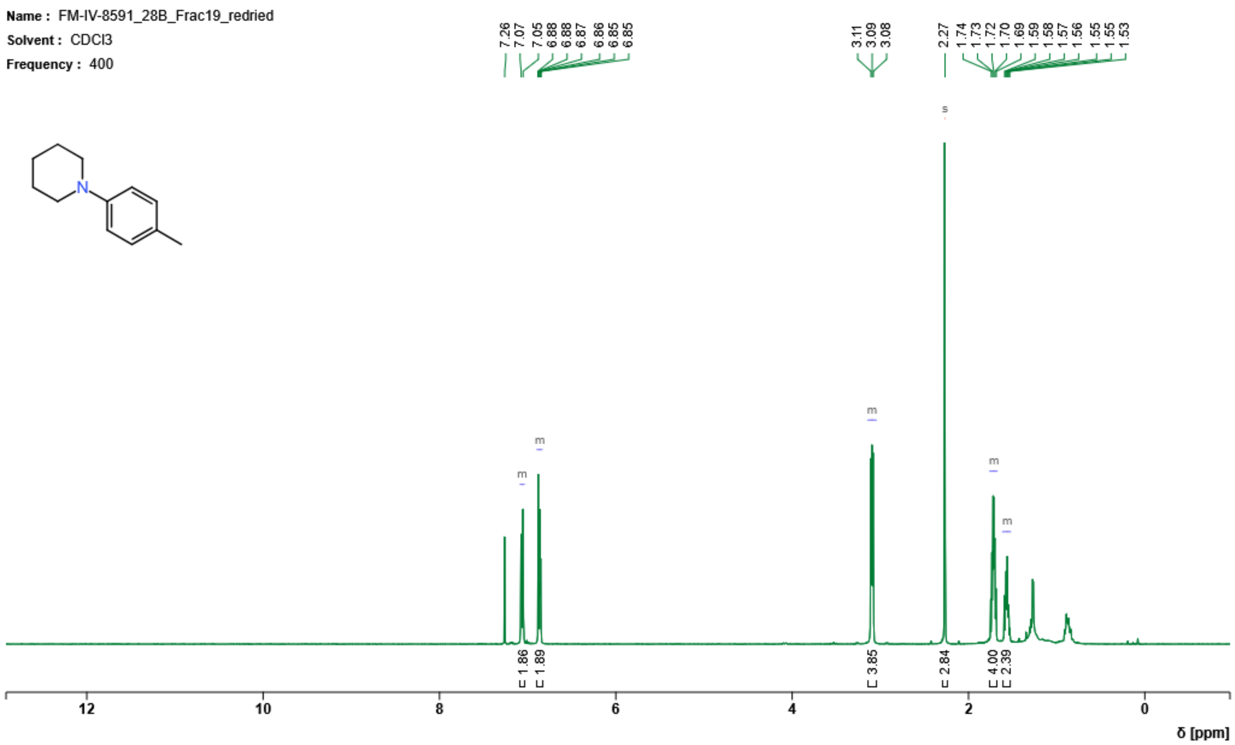

Figure **S18**. ^{1}H NMR spectrum of 1-(*p*-tolyl)piperidine (**3g**) in $CDCl_3$.

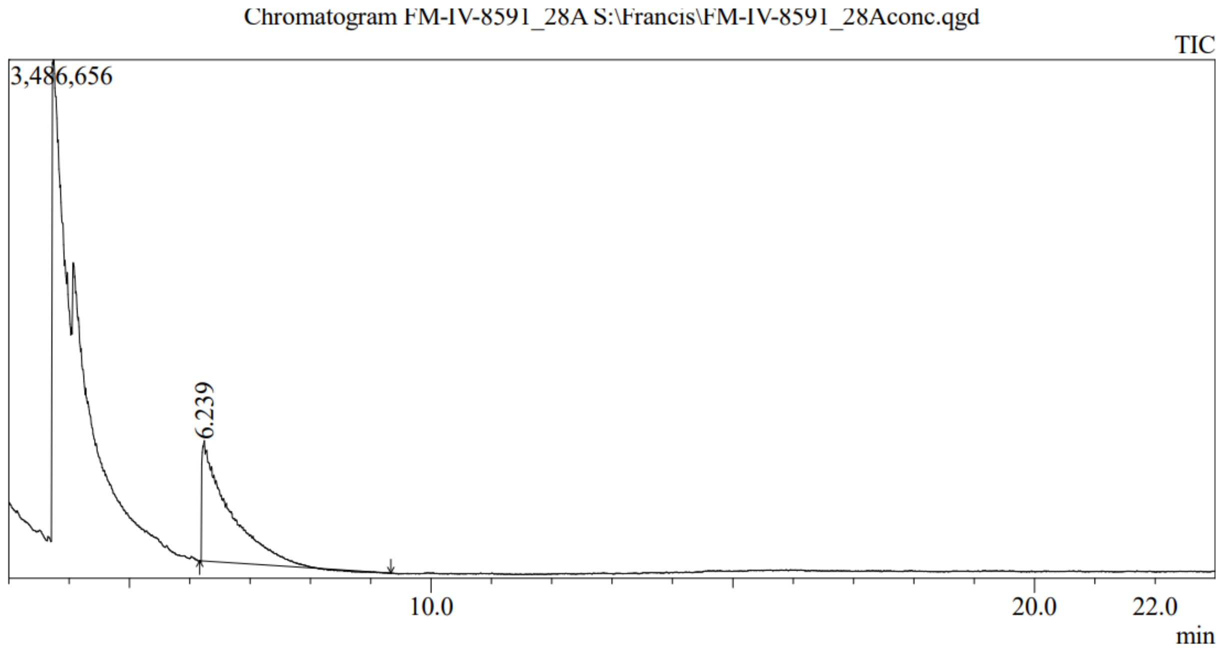

Peak Report TIC

Peak#	R.Time	Area	Area%	Height	A/H	Base m/z	Base Int.
1	6.239	24911861	100.00	809558	30.77	176.10	134417
		24911861	100.00	809558			

Spectrum

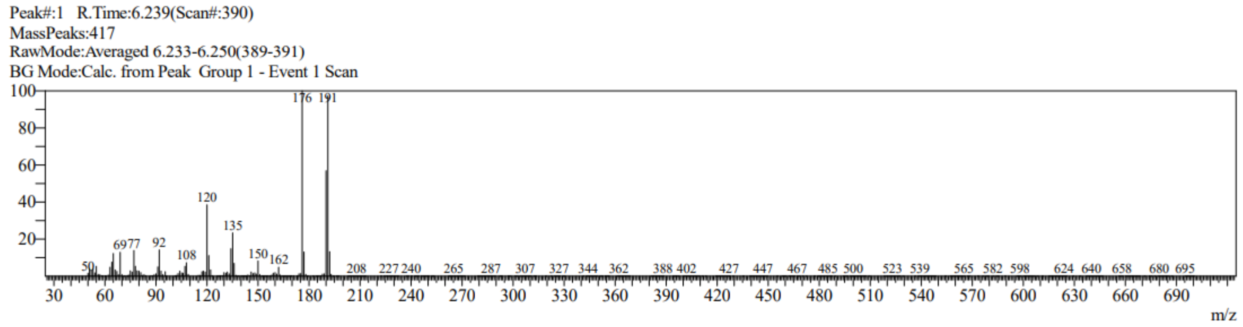

Figure **S19**. Crude GCMS of 1-(4-methoxyphenyl)piperidine (**3h**).

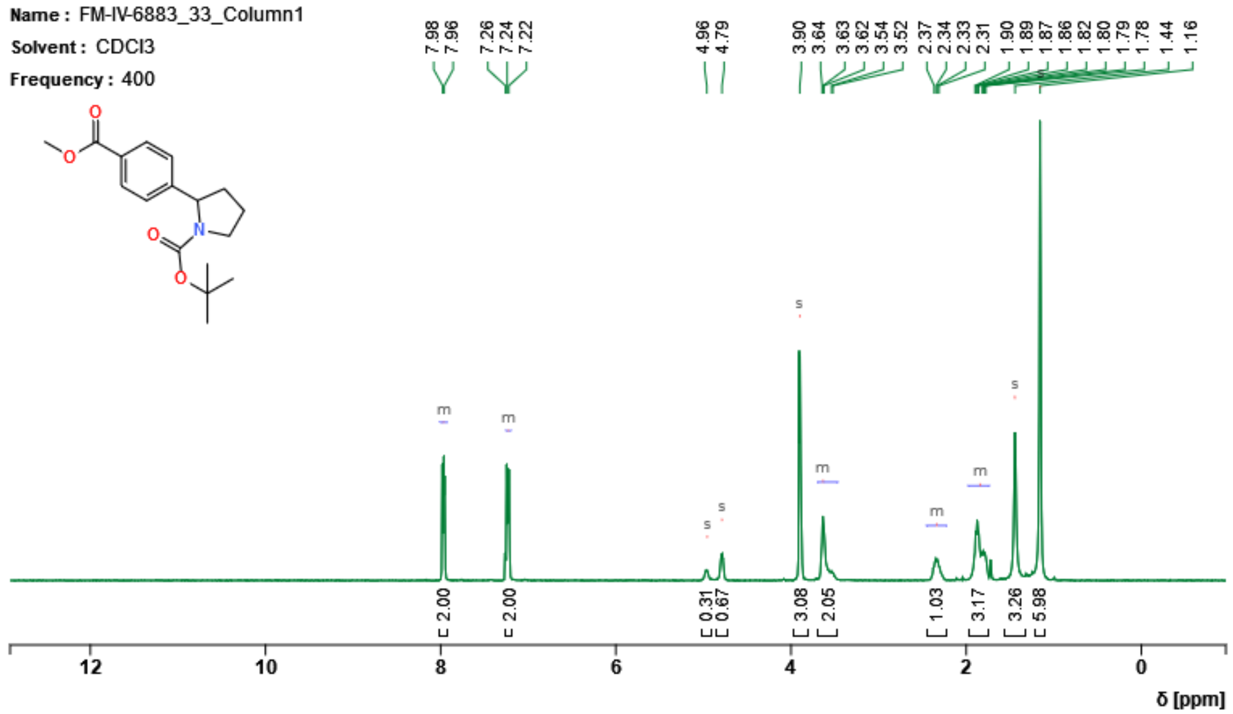

Figure **S20**. ¹H NMR spectrum of *tert*-butyl 2-(4-(methoxycarbonyl)phenyl)pyrrolidine-1-carboxylate (**3i**) in $CDCl_3$.

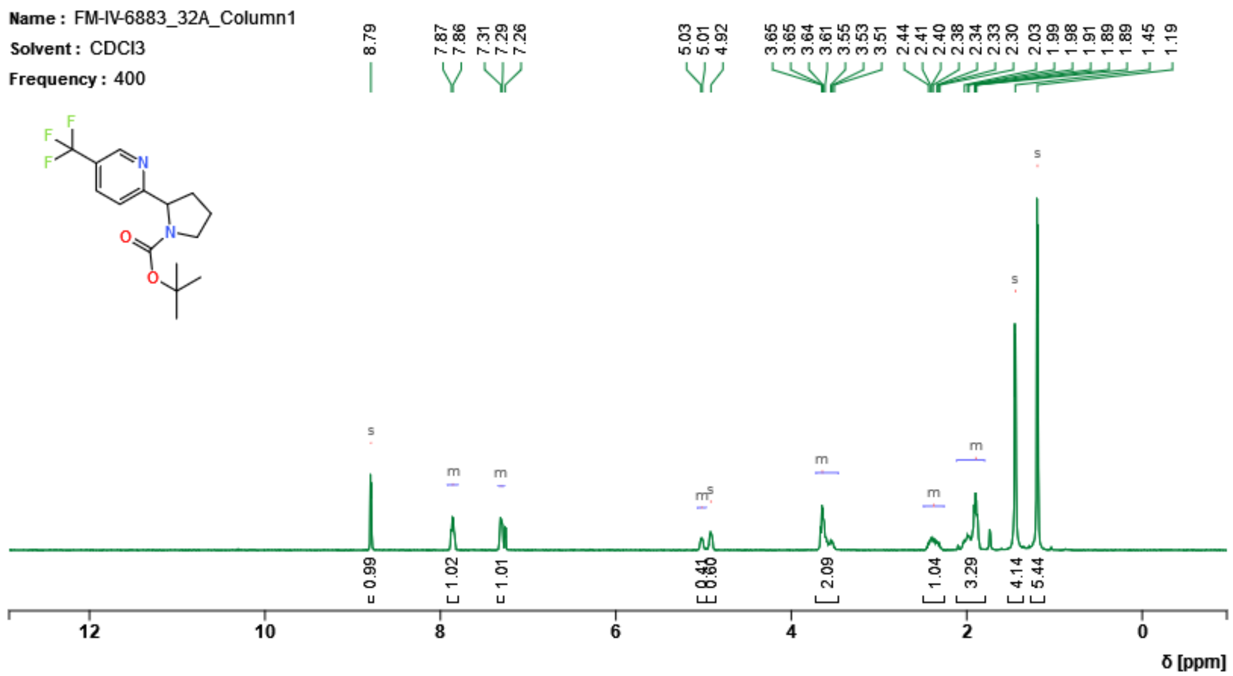

Figure **S21**. ^{1}H NMR spectrum of *tert*-butyl 2-(5-(trifluoromethyl)pyridin-2-yl)pyrrolidine-1-carboxylate (**3j**) in $CDCl_3$.

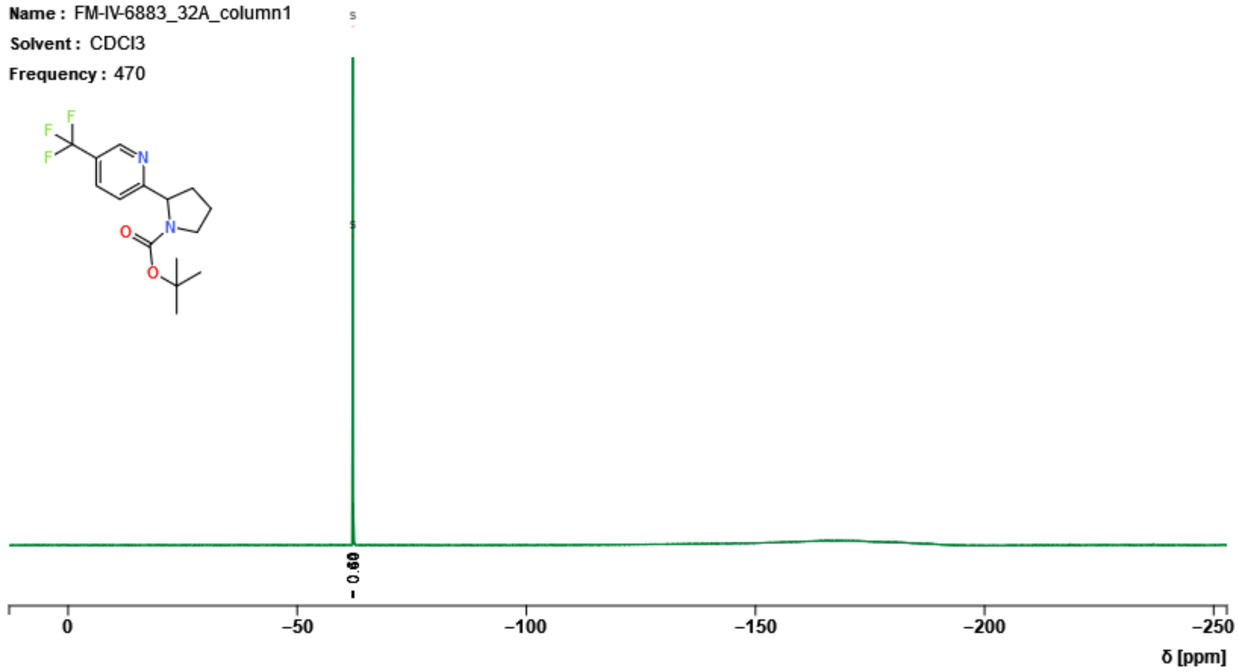

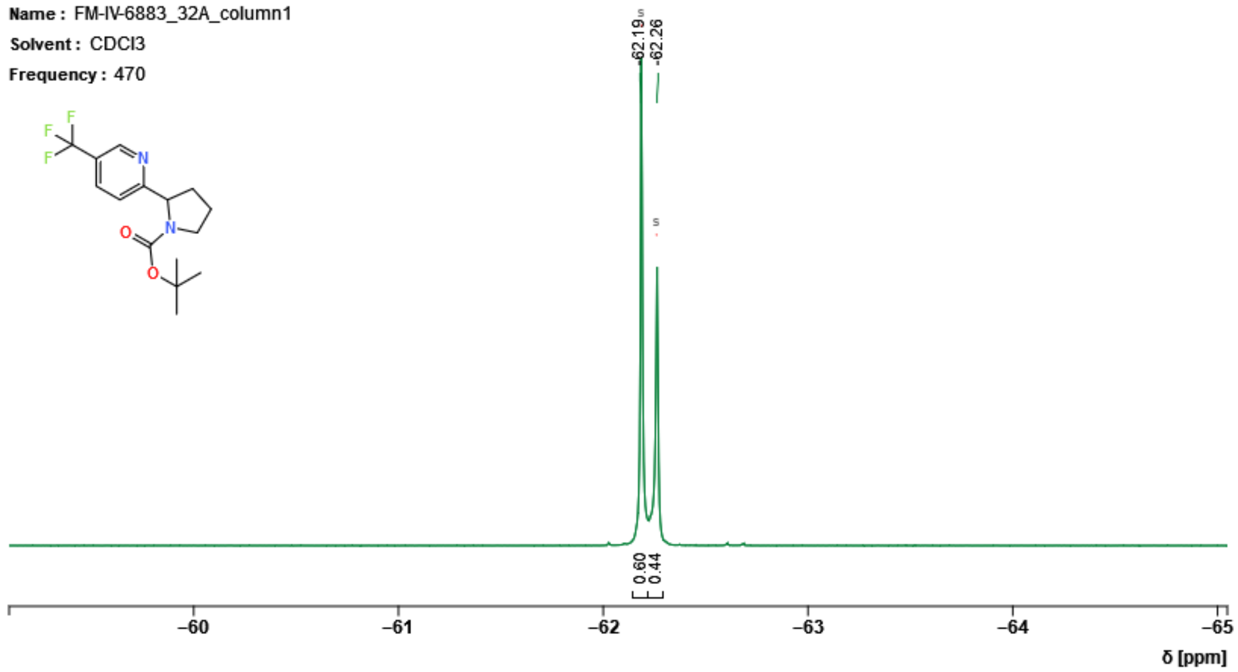

Figure **S22**. ^{19}F NMR spectrum of *tert*-butyl 2-(5-(trifluoromethyl)pyridin-2-yl)pyrrolidine-1-carboxylate (**3j**) in $CDCl_3$.

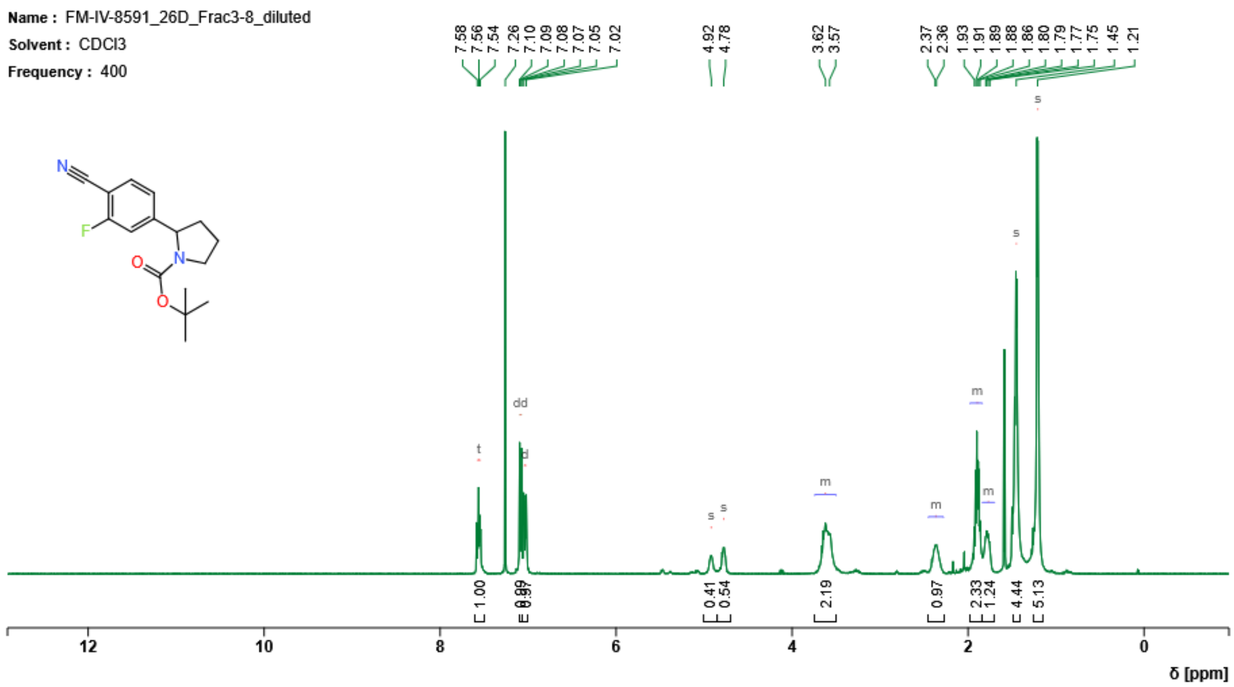

Figure **S23**. ¹H NMR spectrum of *tert*-butyl 2-(4-cyano-3-fluorophenyl)pyrrolidine-1-carboxylate (**3k**) in $CDCl_3$.

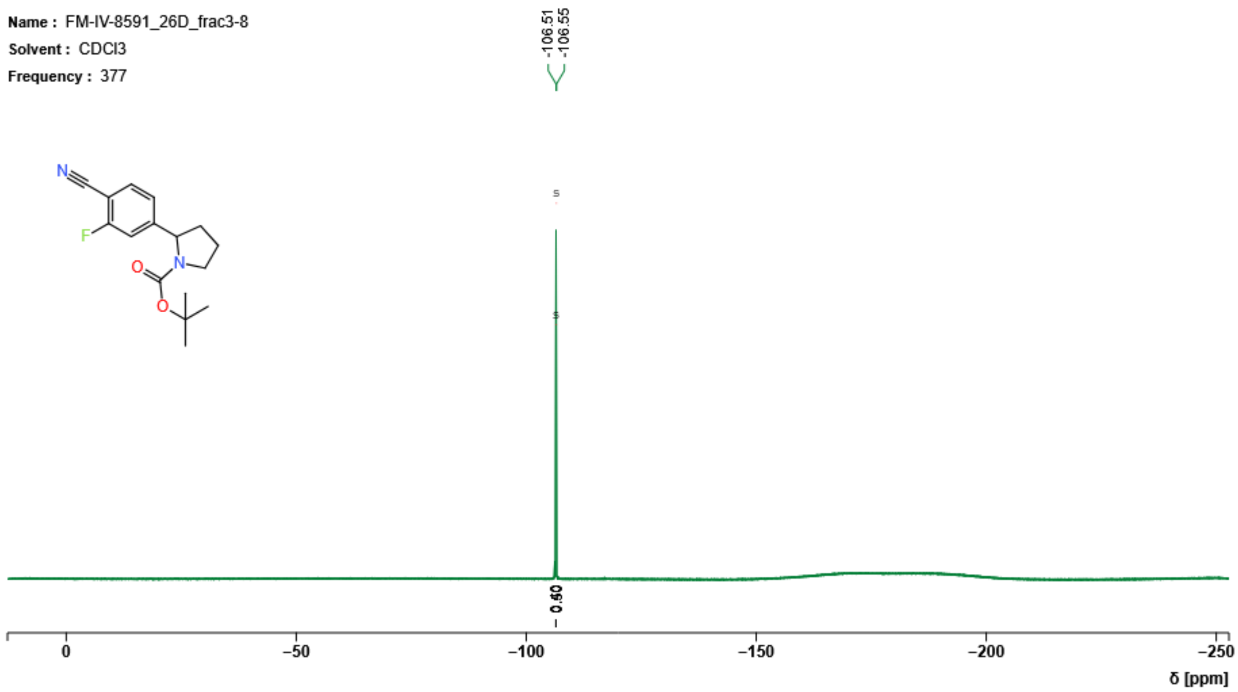

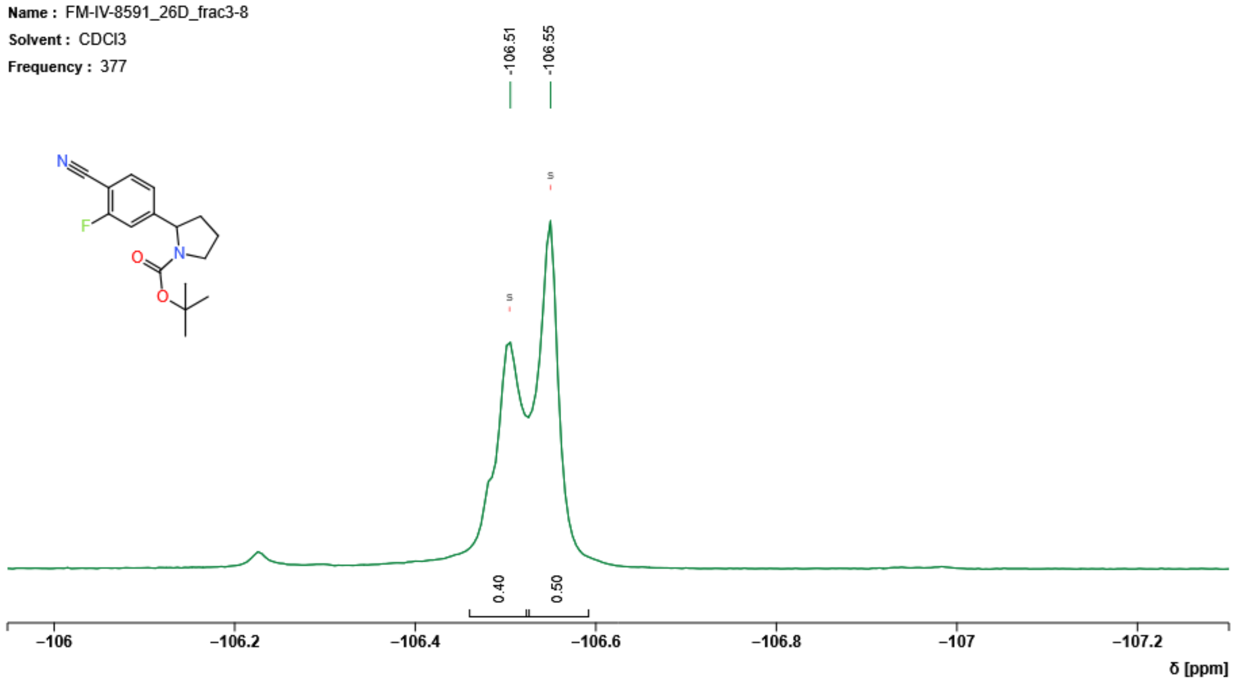

Figure **S24**. ^{19}F NMR spectrum of *tert*-butyl 2-(4-cyano-3-fluorophenyl)pyrrolidine-1-carboxylate (**3k**) in $CDCl_3$.

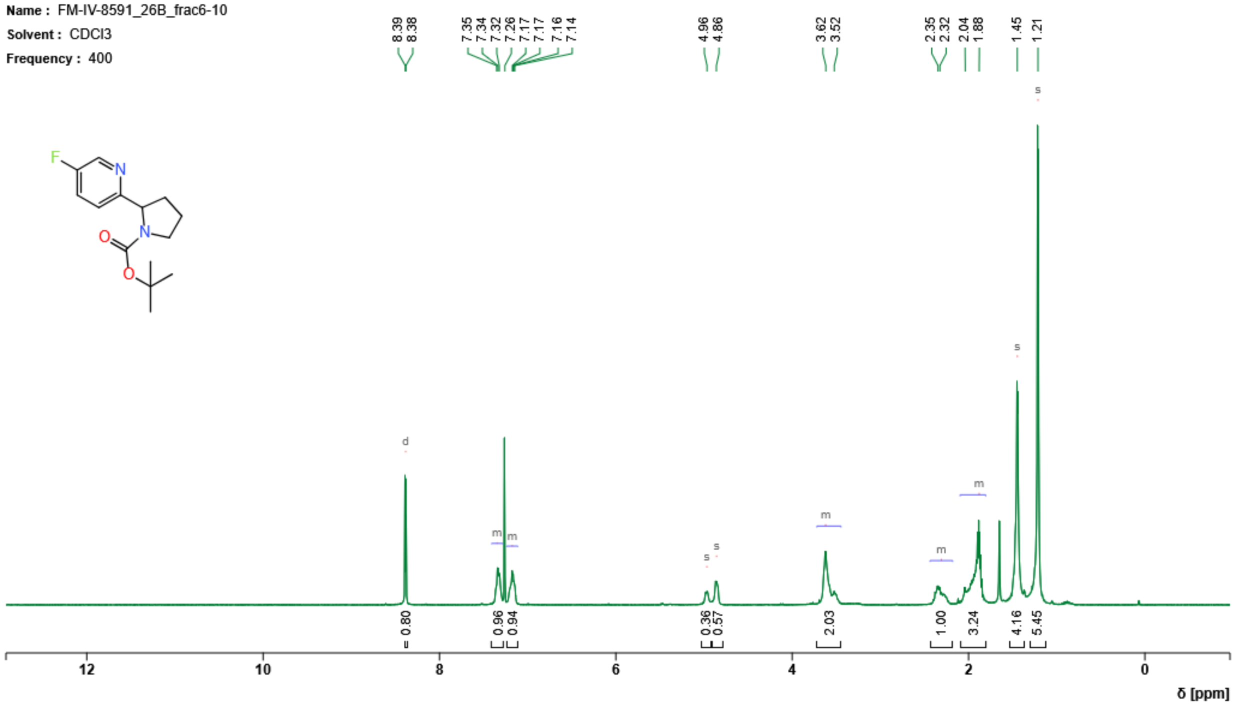

Figure **S25**. ^{1}H NMR spectrum *tert*-butyl-2-(5-fluoropyridin-2-yl)pyrrolidine-1-carboxylate (**3l**) in $CDCl_3$.

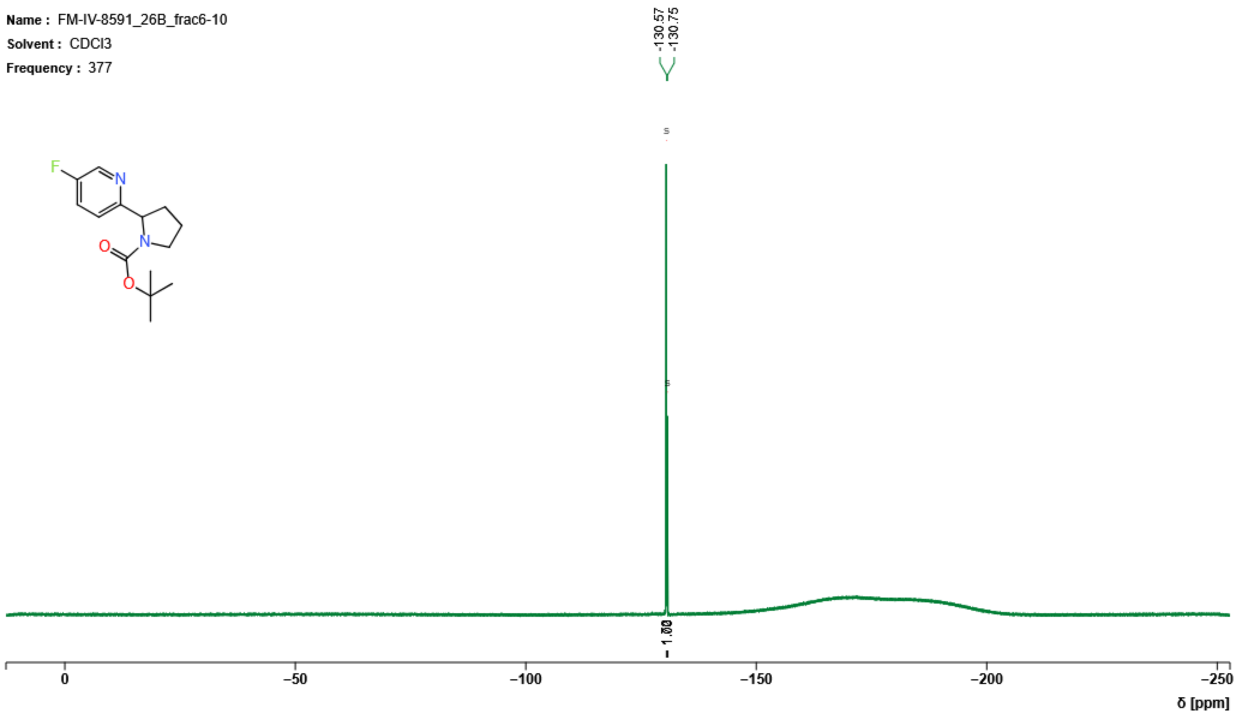

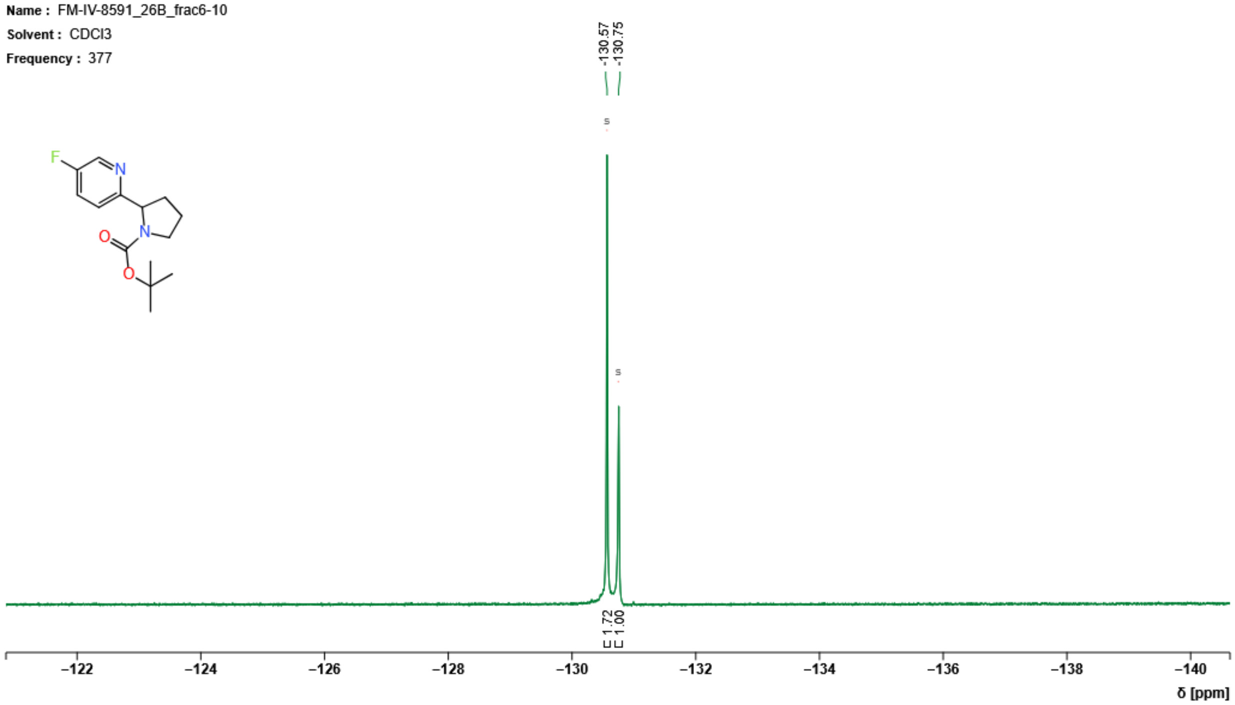

Figure **S26**. ^{19}F NMR spectrum of *tert*-butyl-2-(5-fluoropyridin-2-yl)pyrrolidine-1-carboxylate (**3l**) in $CDCl_3$.

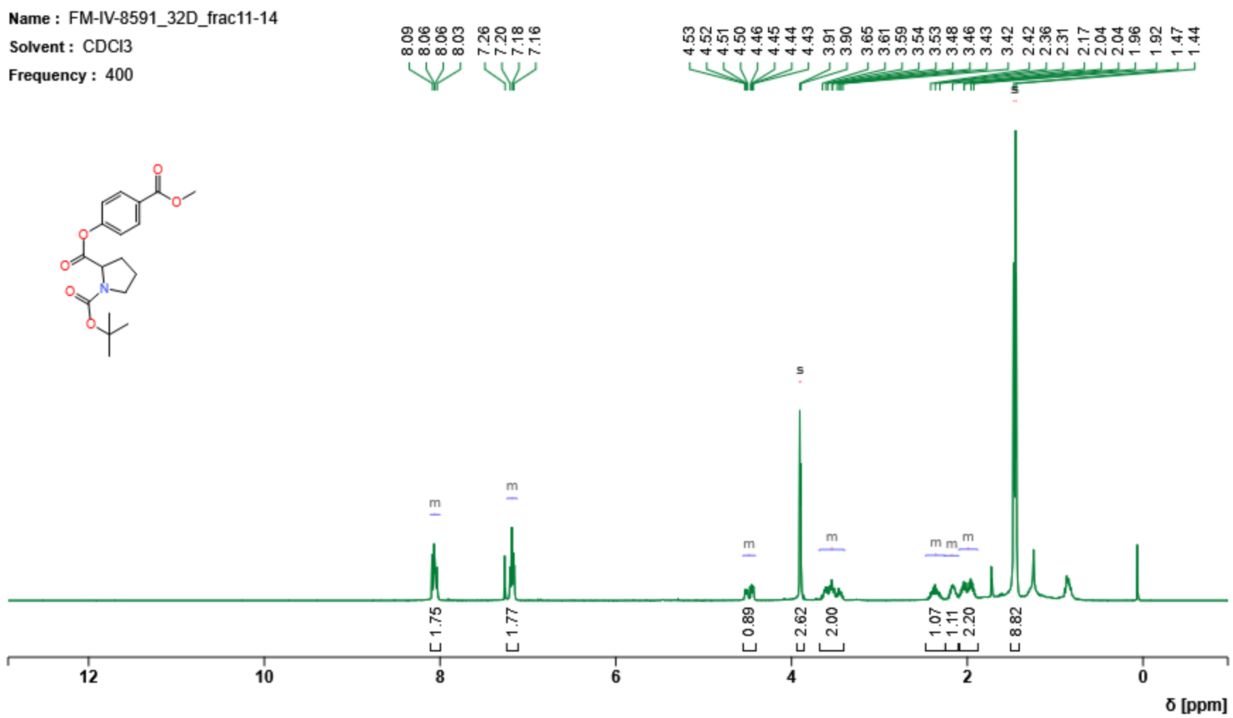

Figure **S27**. ^{1}H NMR spectrum 1-(*tert*-butyl)-2-(4-(methoxycarbonyl)phenyl) pyrrolidine-1,2-dicarboxylate (**3m**) in $CDCl_3$.

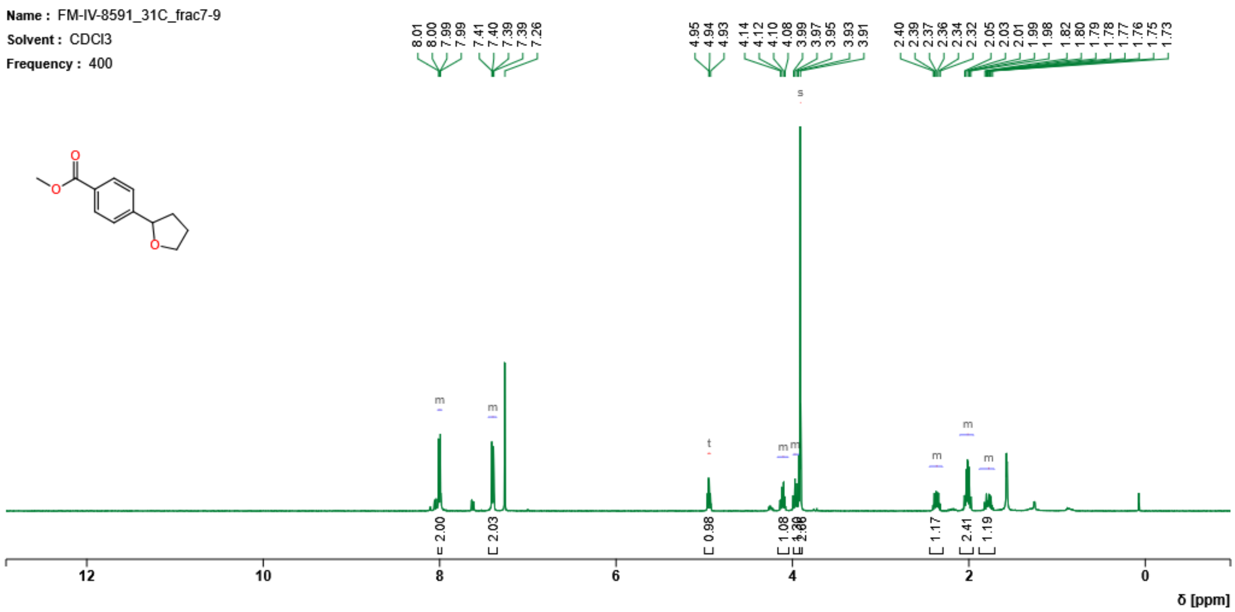

Figure **S28**. ^{1}H NMR spectrum methyl 4-(tetrahydrofuran-2-yl)benzoate (**3n**) in $CDCl_3$.

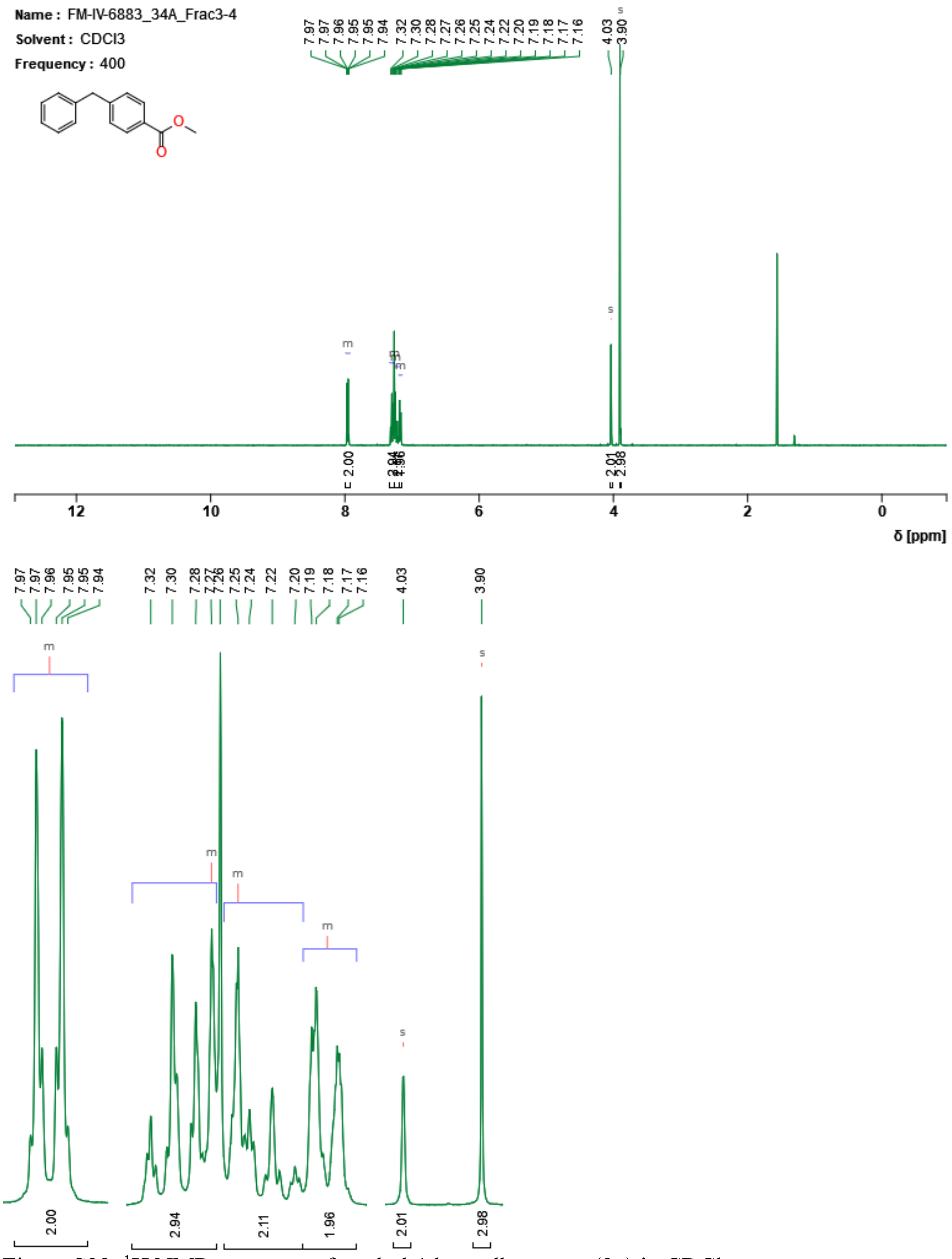

Figure **S29**. ^{1}H NMR spectrum of methyl 4-benzylbenzoate (**3o**) in $CDCl_3$.

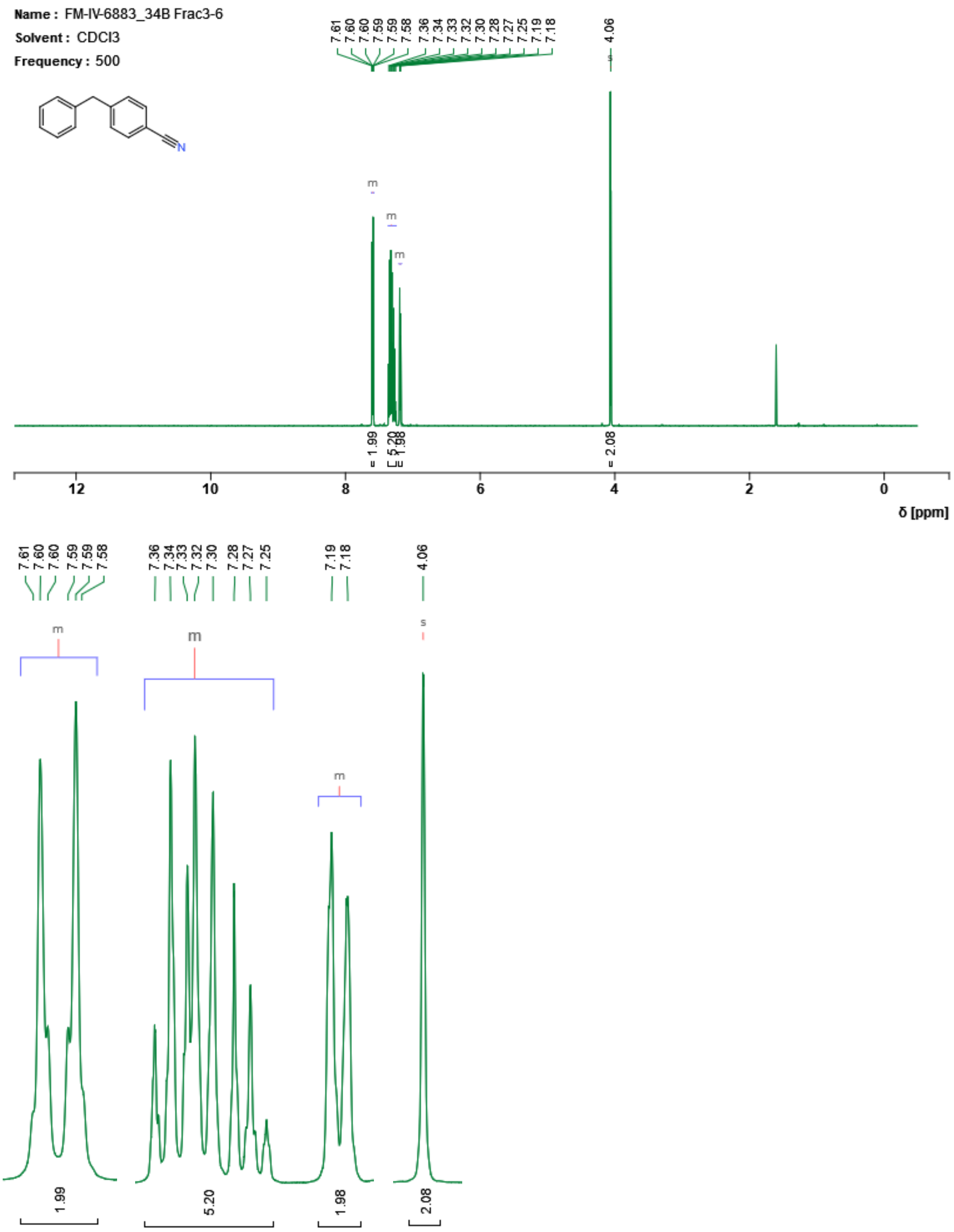

Figure **S30**. ^{1}H NMR spectrum of 4-benzylbenzonitrile (**3p**) in $CDCl_3$.

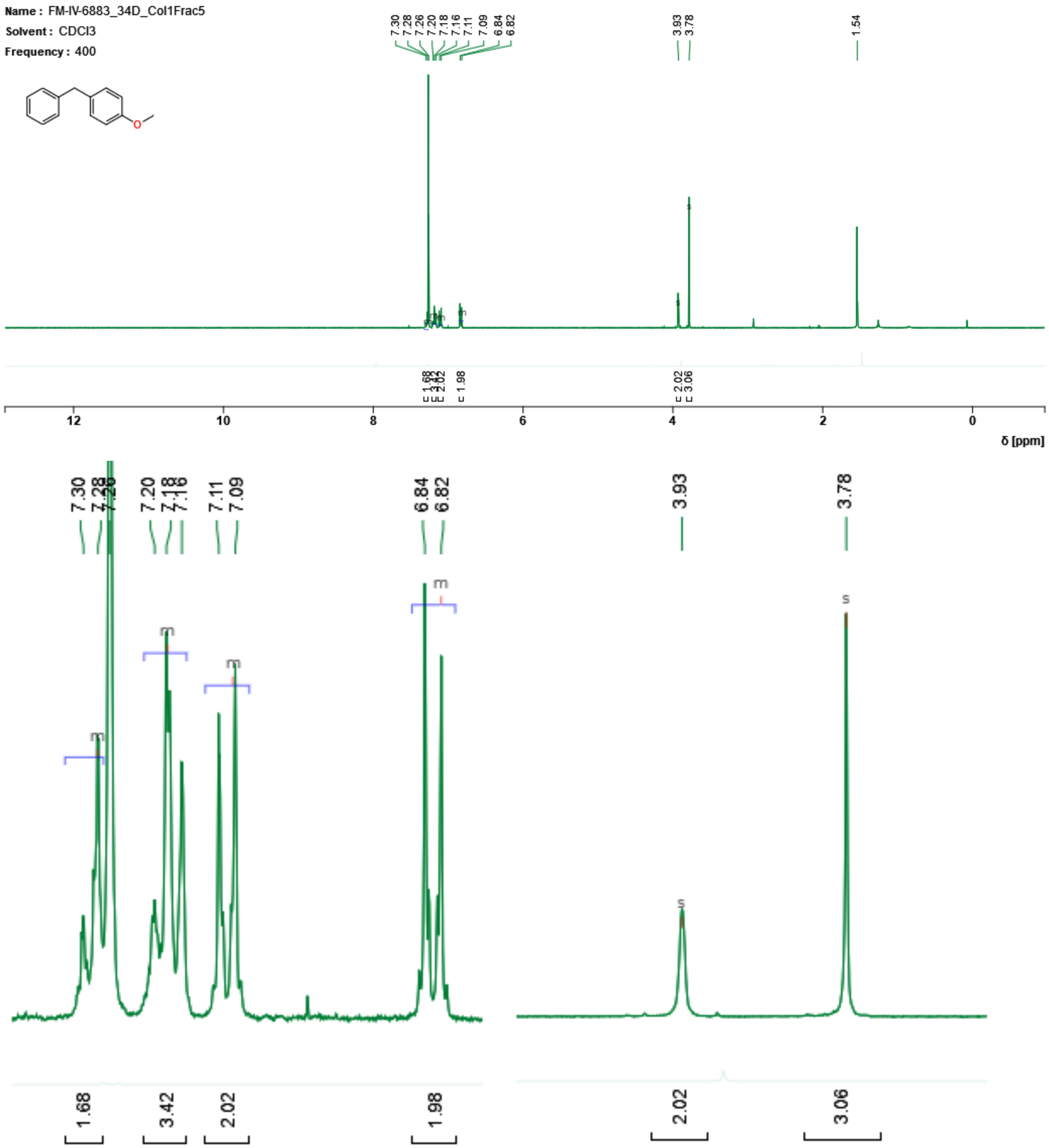

Figure **S31**. ^{1}H NMR spectrum of 1-benzyl-4-methoxybenzene (**3q**) in $CDCl_3$.

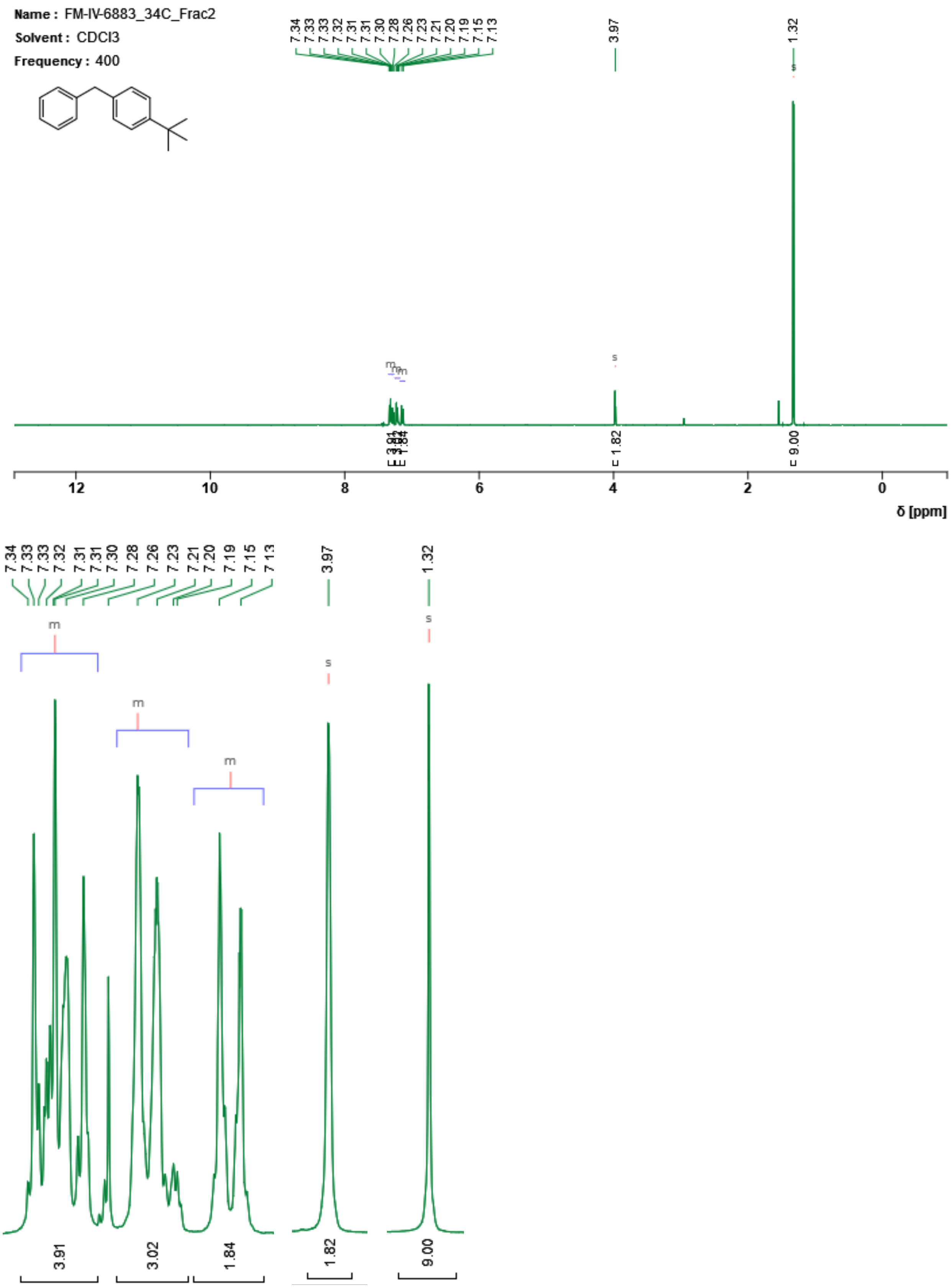

Figure **S32**. ^{1}H NMR spectrum of 1-benzyl-4-(*tert*-butyl)benzene (**3r**) in $CDCl_3$.

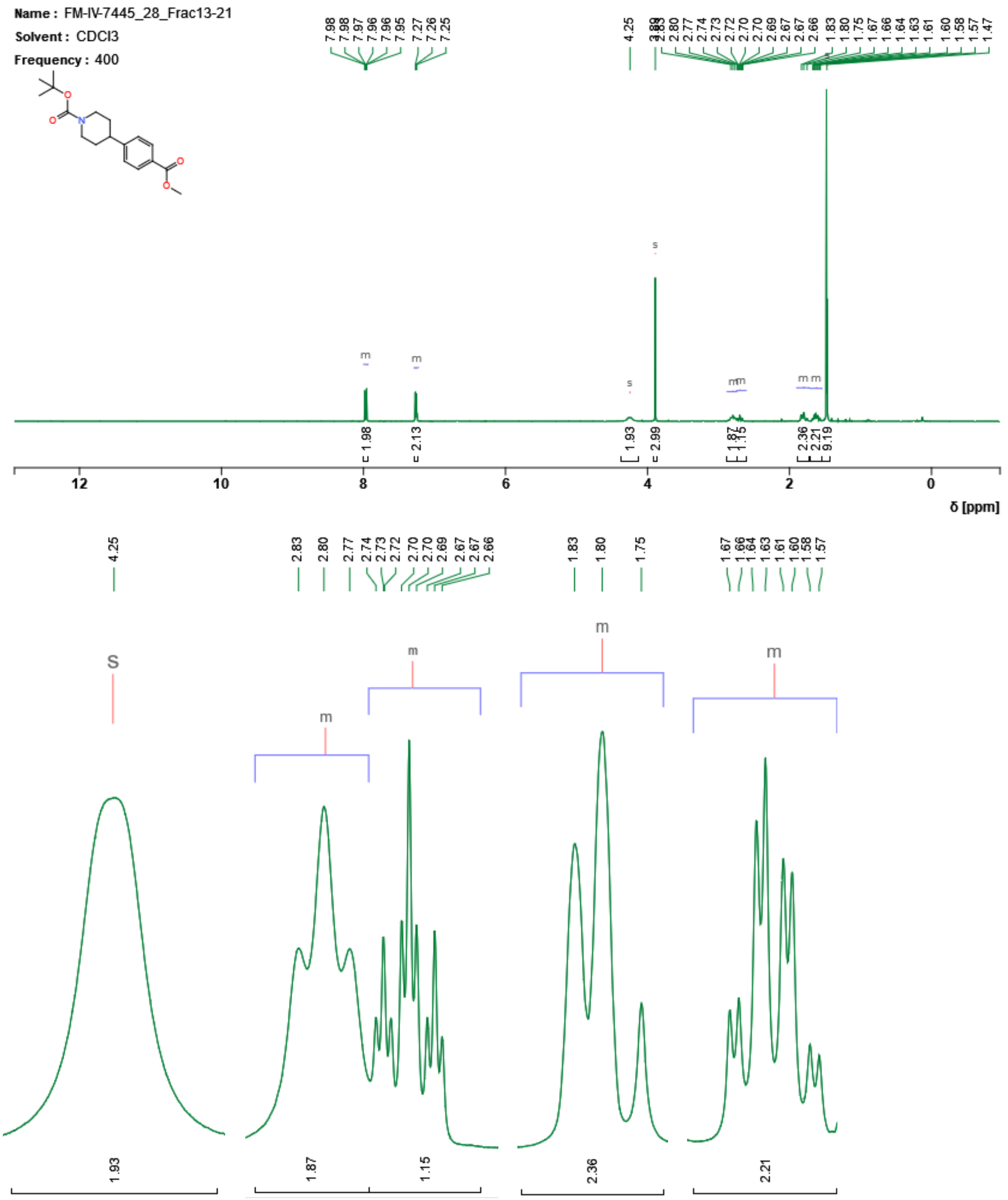

Figure **S33**. ¹H NMR spectrum of *tert*-butyl 4-(4-(methoxycarbonyl)phenyl)piperidine-1-carboxylate (**3s**) in $CDCl_3$.

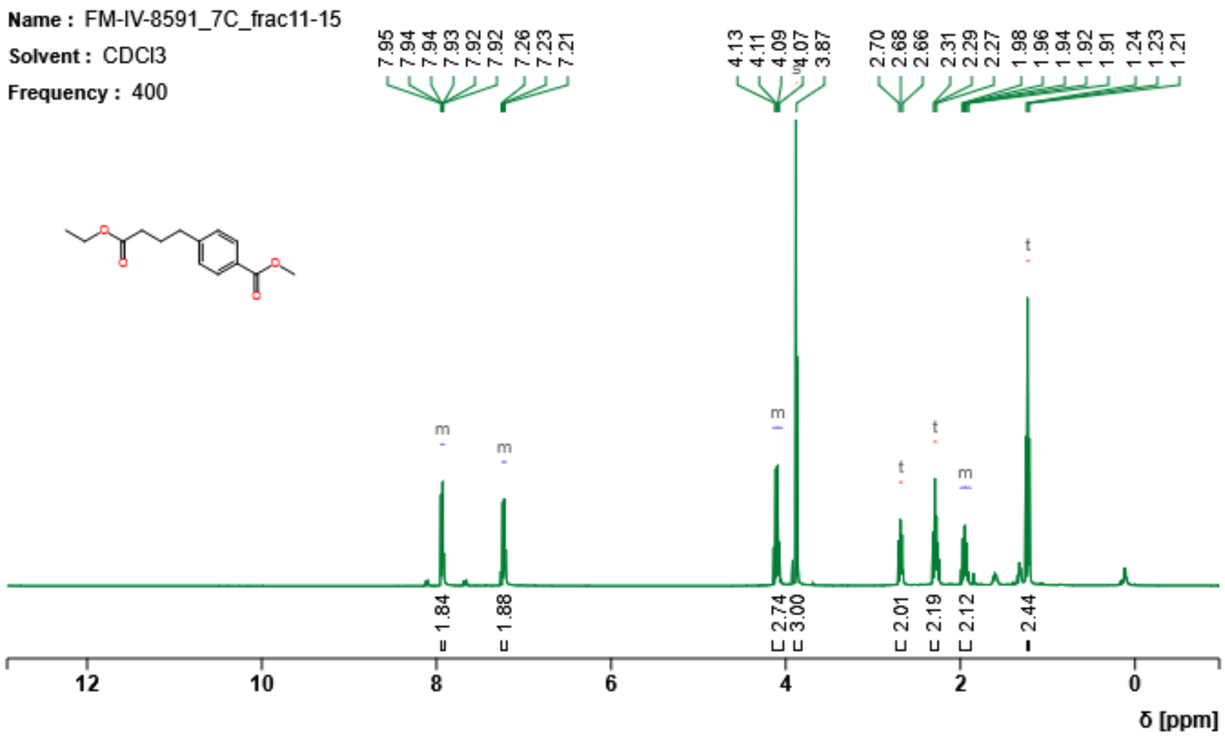

Figure **S34**. ^{1}H NMR spectrum of methyl 4-(4-ethoxy-4-oxobutyl)benzoate (**3t**) in $CDCl_3$.

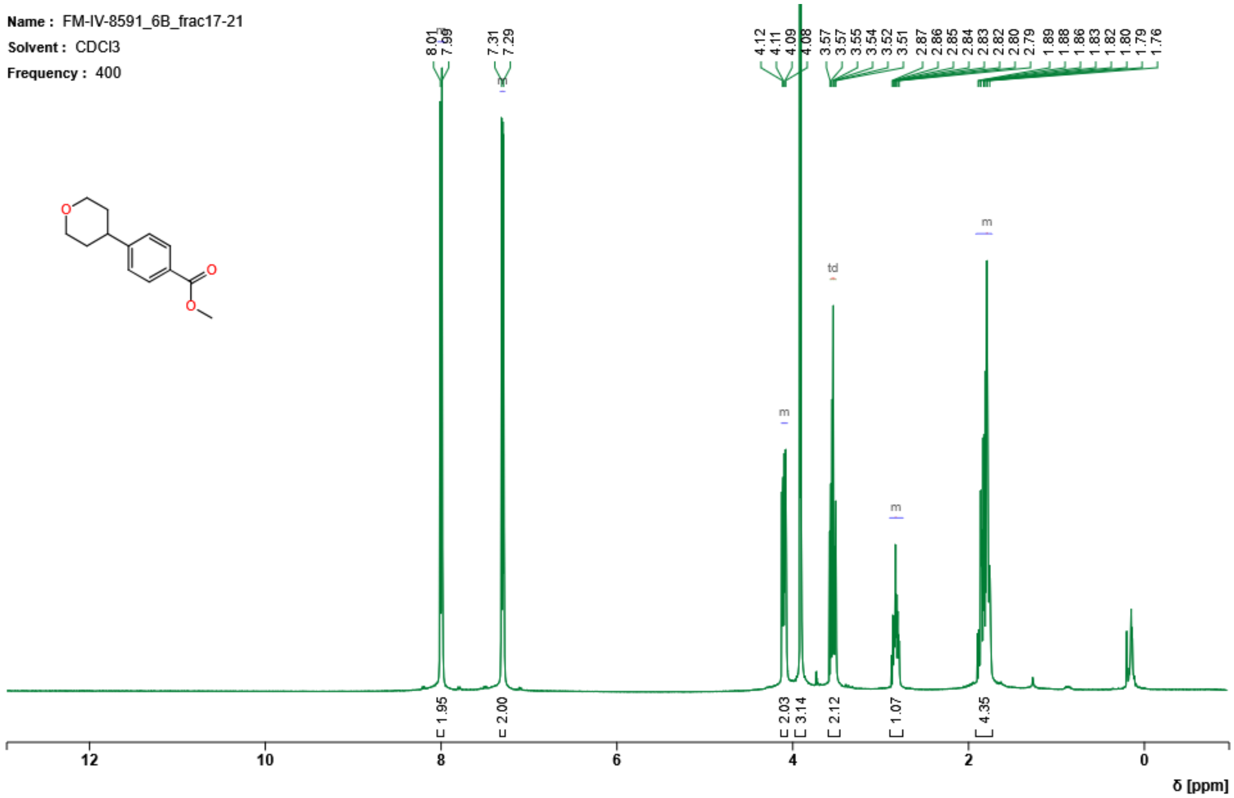

Figure **S35**. ^{1}H NMR spectrum of methyl 4-(tetrahydro-2H-pyran-4-yl)benzoate (**3u**) in $CDCl_3$.

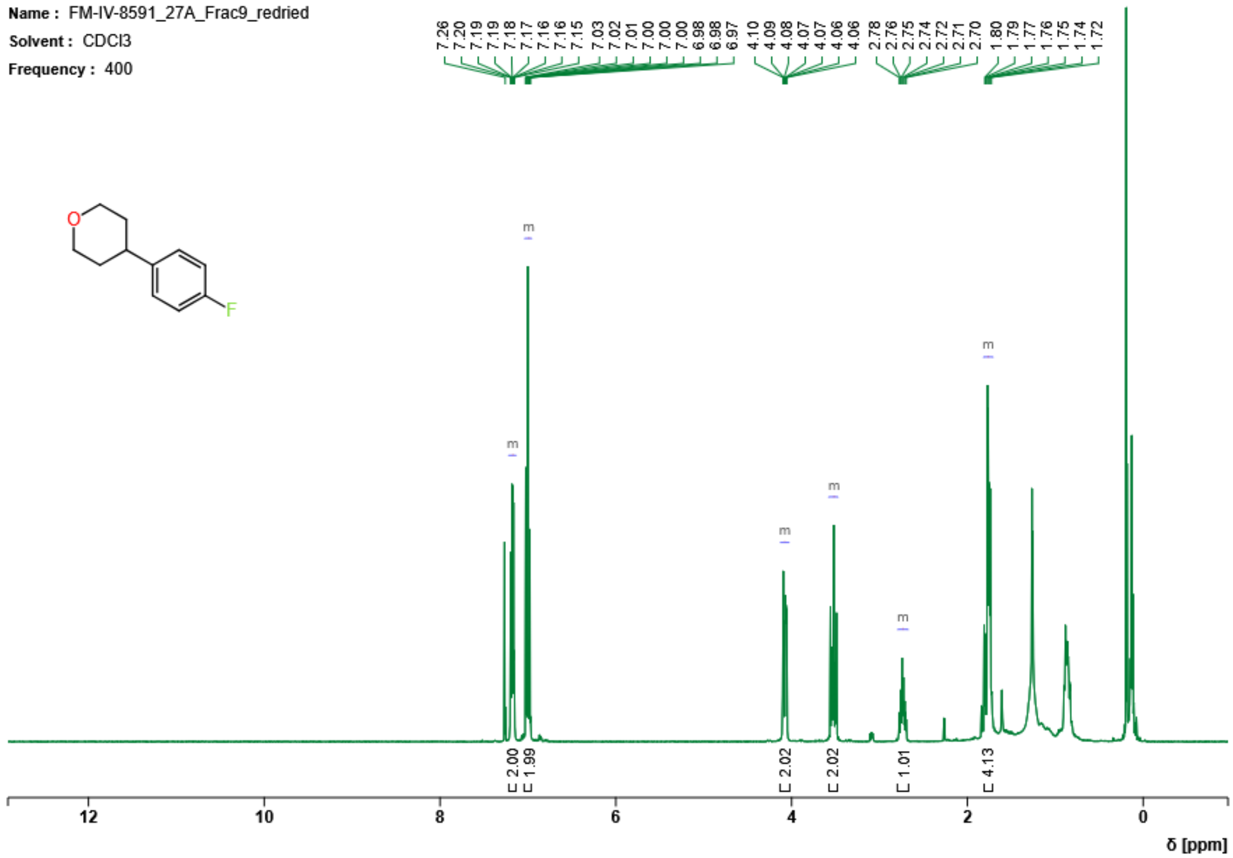

Figure **S36**. ^{1}H NMR spectrum of 4-(4-fluorophenyl)tetrahydro-2H-pyran (**3v**) in $CDCl_3$.

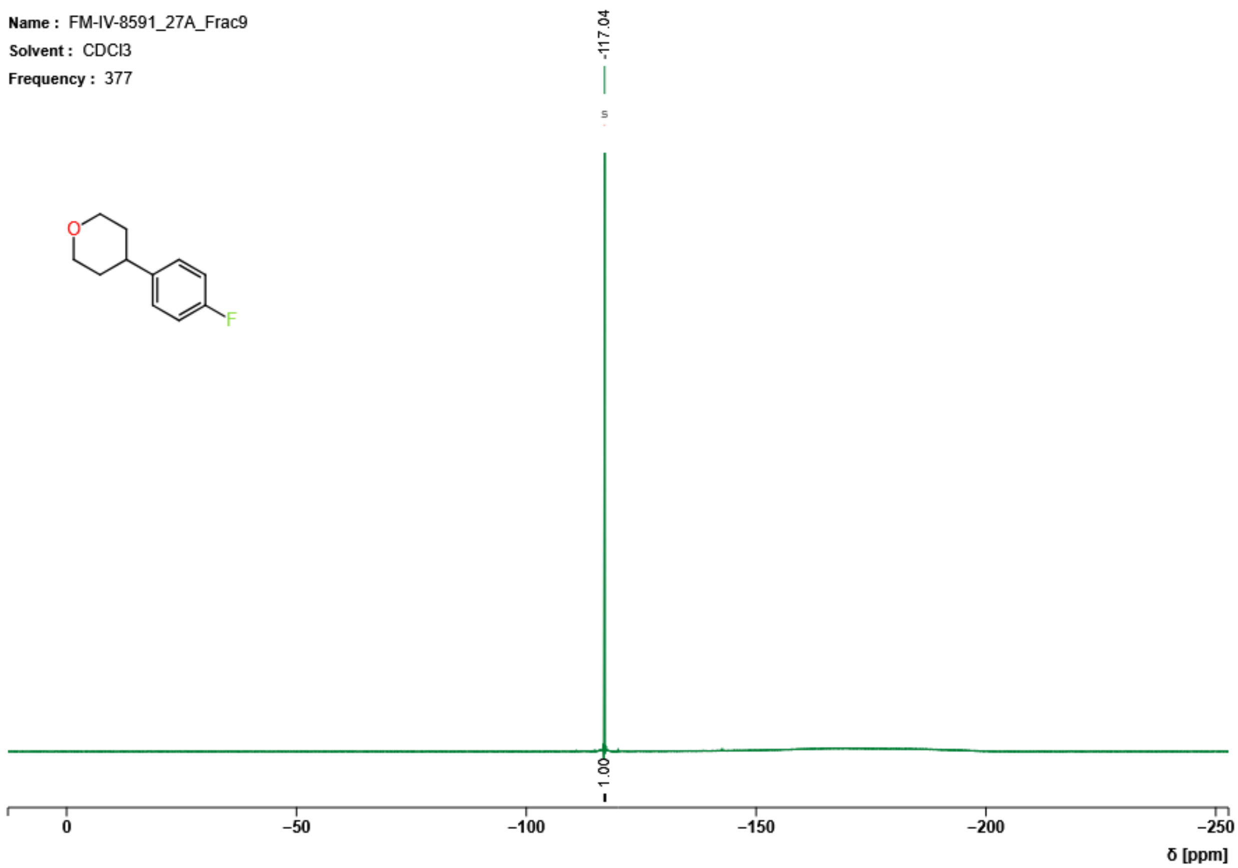

Figure **S37**. ^{19}F NMR spectrum of 4-(4-fluorophenyl)tetrahydro-2H-pyran (**3v**) in $CDCl_3$.

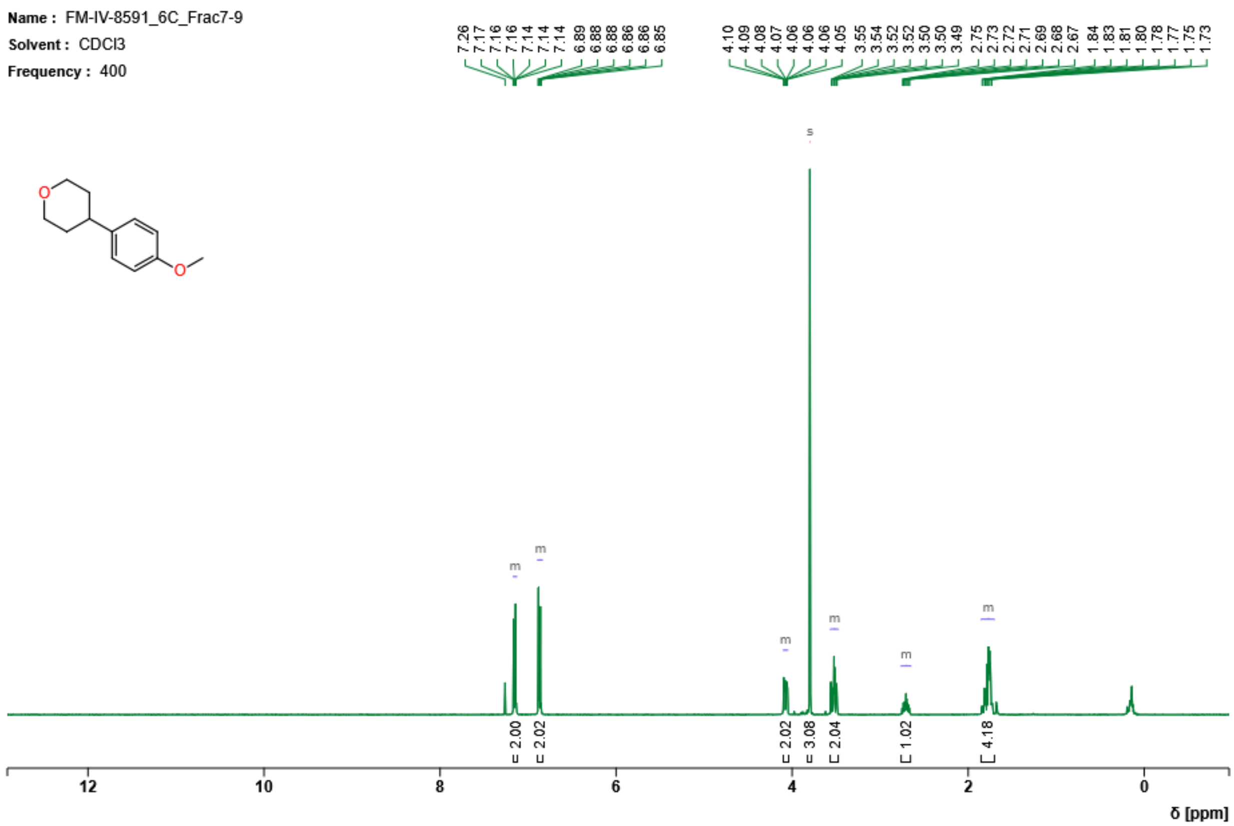

Figure **S38**. ^{1}H NMR spectrum of 4-(4-methoxyphenyl)tetrahydro-2H-pyran (**3w**) in $CDCl_3$.

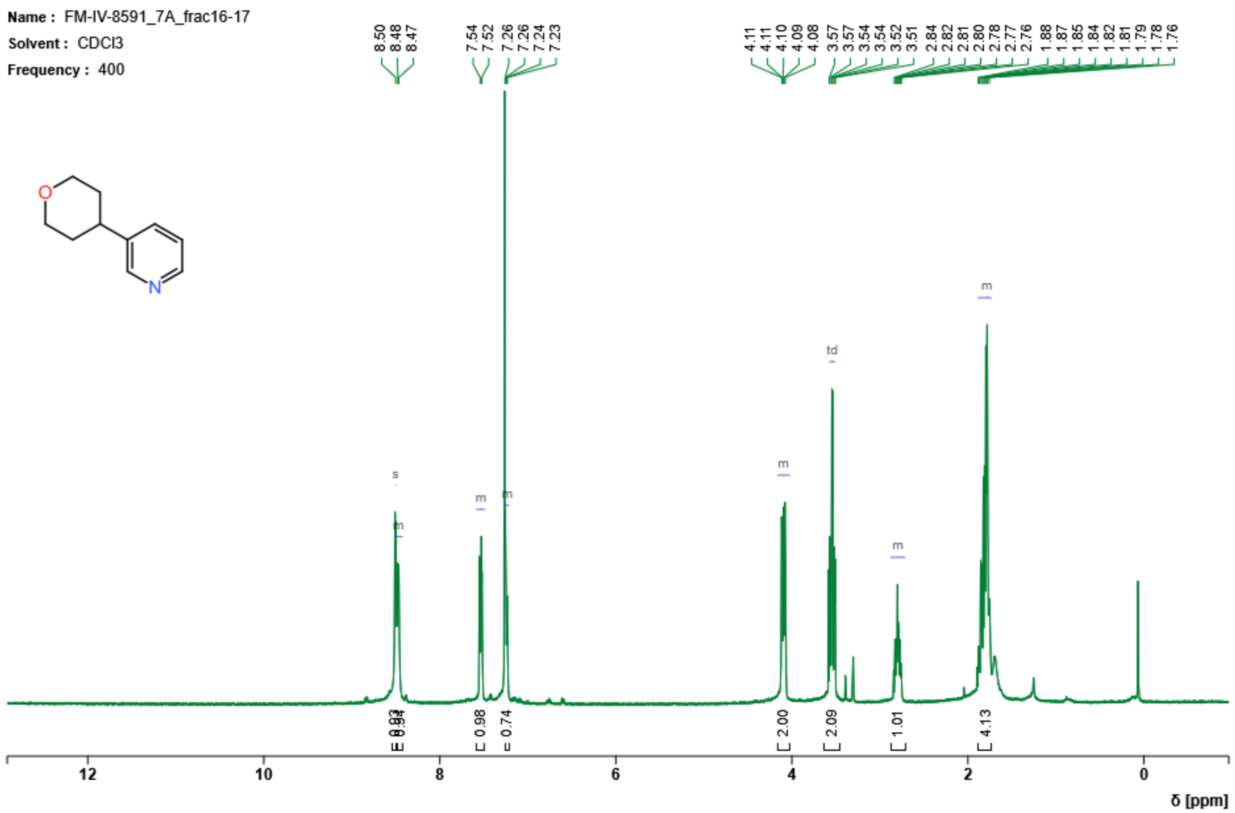

Figure **S39**. ^{1}H NMR spectrum of 3-(tetrahydro-2H-pyran-4-yl)pyridine (**3x**) in $CDCl_3$.

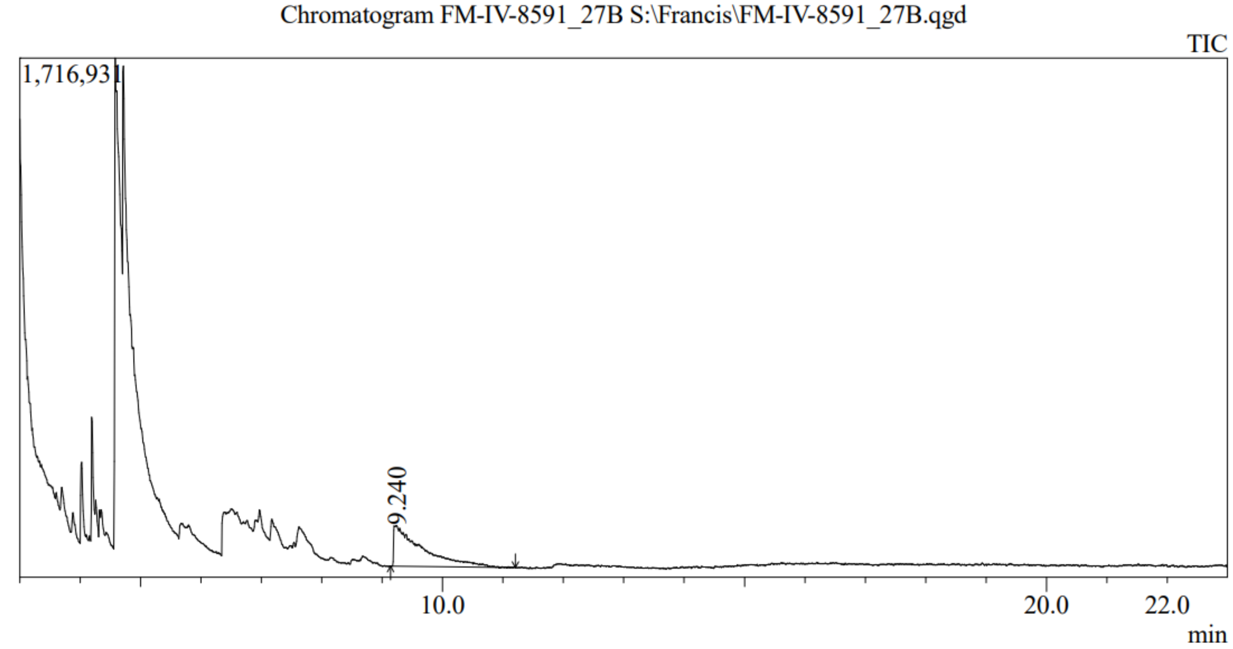

Peak Report TIC

Peak#	R.Time	Area	Area%	Height	A/H	Base m/z	Base Int.
1	9.240	4396056	100.00	134592	32.66	270.20	15758
		4396056	100.00	134592			

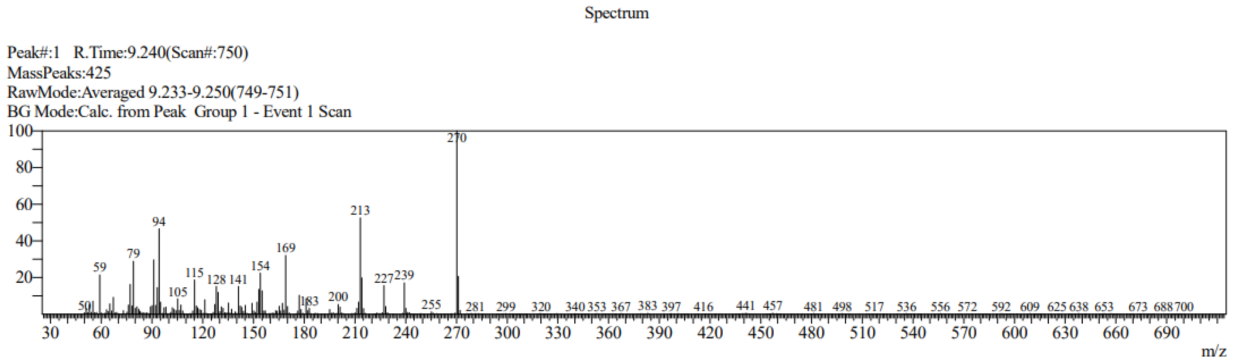

Figure **S40**. Crude GCMS spectrum of methyl 4-(adamantan-1-yl)benzoate (**3y**).

References

(1) Vasu, D.; Fuentes de Arriba, A. L.; Leitch, J. A.; de Gombert, A.; Dixon, D. J. Primary α-tertiary amine synthesis via α-C–H functionalization. *Chem. Sci.* **2019**, *10*, 3401-3407.
(2) Gutiérrez-Bonet, Á.; Tellis, J. C.; Matsui, J. K.; Vara, B. A.; Molander, G. A. 1,4-Dihydropyridines as Alkyl Radical Precursors: Introducing the Aldehyde Feedstock to Nickel/Photoredox Dual Catalysis. *ACS Catal.* **2016**, *6*, 8004-8008.
(3) Wang, C.; Guo, M.; Qi, R.; Shang, Q.; Liu, Q.; Wang, S.; Zhao, L.; Wang, R.; Xu, Z. Visible-Light-Driven, Copper-Catalyzed Decarboxylative C(sp3)−H Alkylation of Glycine and Peptides. *Angew. Chem. Int. Ed.* **2018**, *57*, 15841-15846.
(4) Millward, F.; Zysman-Colman, E. Mechanophotocatalysis: A Generalizable Approach to Solvent-minimized Photocatalytic Reactions for Organic Synthesis. *Angew. Chem. Int. Ed.* **2024**, *63*, e202316169.
(5) Buettner, C. S.; Stavagna, C.; Tilby, M. J.; Górski, B.; Douglas, J. J.; Yasukawa, N.; Leonori, D. Synthesis and Suzuki–Miyaura Cross-Coupling of Alkyl Amine-Boranes. A Boryl Radical-Enabled Strategy. *J. Am. Chem. Soc.* **2024**, *146*, 24042-24052.
(6) Hämmerling, L.; Zysman-Colman, E. Building a photocatalyst library of MR-TADF compounds with tunable excited-state redox potentials. *Chem Catal.* **2024**, *4*, 101061.
(7) Fan, Y.; Kang, D. W.; Labalme, S.; Lin, W. A Spirobifluorene-Based Covalent Organic Framework for Dual Photoredox and Nickel Catalysis. *J. Am. Chem. Soc.* **2023**, *145*, 25074-25079.
(8) Sun, R.; Qin, Y.; Nocera, D. G. General Paradigm in Photoredox Nickel-Catalyzed Cross-Coupling Allows for Light-Free Access to Reactivity. *Angew. Chem. Int. Ed.* **2020**, *59*, 9527-9533.
(9) Kudisch, M.; Lim, C.-H.; Thordarson, P.; Miyake, G. M. Energy Transfer to Ni-Amine Complexes in Dual Catalytic, Light-Driven C–N Cross-Coupling Reactions. *J. Am. Chem. Soc.* **2019**, *141*, 19479-19486.
(10) Mao, X.; Hu, W.; Wu, M.; Jin, Y.; Zhao, J.; Xu, Y.; Li, B.; Wang, W.; Wu, Y.; Zhang, J.; et al. Discovery of a Novel Non-invasive AR PROTAC Degrader for the Topical Treatment of Androgenetic Alopecia. *J. Med. Chem.* **2024**, *67*, 22218-22244.
(11) XU, H. L., Jia; SHI, Jingjing; ZANG, Yi; SUN, Dandan; LIU, Mingliang; XIE, Rongrong; YOU, Erli; GAO, Lixin; TAN, Qian. FXR SMALL MOLECULE AGONIST AND PREPARATION METHOD THEREFOR AND USE THEREOF. EP3957640A1.
(12) Song, D.; Qin, Y.; Liu, Y.; Wang, J.; Yang, H.; Zheng, Z.; Xu, F.; Bao, X.; Chen, G. A recyclable ciprofloxacin polymer ligand for copper-catalyzed coupling of (hetero)aryl halide aminations. *New J. Chem.* **2022**, *46*, 19100-19103.
(13) Luo, J.; Zhang, J. Donor–Acceptor Fluorophores for Visible-Light-Promoted Organic Synthesis: Photoredox/Ni Dual Catalytic C(sp3)–C(sp2) Cross-Coupling. *ACS Catal.* **2016**, *6*, 873-877.
(14) Zuo, Z.; Ahneman, D. T.; Chu, L.; Terrett, J. A.; Doyle, A. G.; MacMillan, D. W. C. Merging photoredox with nickel catalysis: Coupling of α-carboxyl sp3-carbons with aryl halides. *Science* **2014**, *345*, 437-440.
(15) Jakobi, M.; Sparr, C. Streamlined Synthesis of Aminoacridinium Photocatalysts with Improved Photostability. *Org. Process Res. Dev.* **2022**, *26*, 2756-2760.
(16) Tellis, J. C.; Primer, D. N.; Molander, G. A. Single-electron transmetalation in organoboron cross-coupling by photoredox/nickel dual catalysis. *Science* **2014**, *345*, 433-436.

(17) Lukas, F.; Findlay, M. T.; Fillols, M.; Templ, J.; Savino, E.; Martin, B.; Allmendinger, S.; Furegati, M.; Noël, T. Graphitic Carbon Nitride as a Photocatalyst for Decarboxylative C(sp2)C(sp3) Couplings via Nickel Catalysis. *Angew. Chem. Int. Ed.* **2024**, *63*, e202405902.
(18) Zhang, P.; Le, C. C.; Macmillan, D. W. C. Silyl Radical Activation of Alkyl Halides in Metallaphotoredox Catalysis: A Unique Pathway for Cross-Electrophile Coupling. *J. Am. Chem. Soc.* **2016**, *138*, 8084-8087.
(19) Borlinghaus, N.; Schönfeld, B.; Heitz, S.; Klee, J.; Vukelić, S.; Braje, W. M.; Jolit, A. Enabling Metallophotoredox Catalysis in Parallel Solution-Phase Synthesis Using Disintegrating Reagent Tablets. *J. Org. Chem.* **2021**, *86*, 16535-16547.
(20) Gandini, T.; Dolcini, L.; Di Leo, L.; Fornara, M.; Bossi, A.; Penconi, M.; Dal Corso, A.; Gennari, C.; Pignataro, L. Metallaphotoredox CO and CN Cross-Coupling Using Donor-Acceptor Cyanoarene Photocatalysts. *ChemCatChem* **2022**, *14*, e202200990.
(21) Lim, C. H.; Kudisch, M.; Liu, B.; Miyake, G. M. C-N Cross-Coupling via Photoexcitation of Nickel-Amine Complexes. *J. Am. Chem. Soc.* **2018**, *140*, 7667-7673.
(22) Saeb, R.; Boulenger, B.; Cornella, J. "Naked Nickel"-Catalyzed Amination of Heteroaryl Bromides. *Org. Lett.* **2024**, *26*, 5928-5933.
(23) Park, B. Y.; Pirnot, M. T.; Buchwald, S. L. Visible Light-Mediated (Hetero)aryl Amination Using Ni(II) Salts and Photoredox Catalysis in Flow: A Synthesis of Tetracaine. *J. Org. Chem.* **2020**, *85*, 3234-3244.
(24) Constantin, T.; Górski, B.; Tilby, M. J.; Chelli, S.; Juliá, F.; Llaveria, J.; Gillen, K. J.; Zipse, H.; Lakhdar, S.; Leonori, D. Halogen-atom and group transfer reactivity enabled by hydrogen tunneling. *Science* **2022**, *377*, 1323-1328.
(25) Bryden, M. A.; Millward, F.; Lee, O. S.; Cork, L.; Gather, M. C.; Steffen, A.; Zysman-Colman, E. Lessons learnt in photocatalysis – the influence of solvent polarity and the photostability of the photocatalyst. *Chem. Sci.* **2024**, *15*, 3741-3757.
(26) Till, N. A.; Tian, L.; Dong, Z.; Scholes, G. D.; MacMillan, D. W. C. Mechanistic Analysis of Metallaphotoredox C–N Coupling: Photocatalysis Initiates and Perpetuates Ni(I)/Ni(III) Coupling Activity. *J. Am. Chem. Soc.* **2020**, *142*, 15830-15841.
(27) Zhao, P.; Luo, Y. W.; Xue, T.; Zhang, A. J.; Lu, J. X. Nickel-catalyzed Electrochemical Coupling of Phenyl Halide and Study of Mechanism. *Chin. J. Chem.* **2006**, *24*, 877-880.
(28) DiLuzio, S.; Kannadi Valloli, L.; Kudisch, M.; Chambers, D. T.; Rumbles, G.; Reid, O. G.; Bird, M. J.; Sayre, H. J. Reconceptualizing the IrIII Role in Metallaphotoredox Catalysis: From Strong Photooxidant to Potent Energy Donor. *ACS Catal.* **2024**, *14*, 11378-11388.
(29) Yasu, Y.; Koike, T.; Akita, M. Visible Light-Induced Selective Generation of Radicals from Organoborates by Photoredox Catalysis. *Adv. Synth. Catal.* **2012**, *354*, 3414-3420.
(30) Pavlishchuk, V. V.; Addison, A. W. Conversion constants for redox potentials measured versus different reference electrodes in acetonitrile solutions at 25°C. *Inorg. Chim. Acta* **2000**, *298*, 97-102.
(31) Connell, T. U.; Fraser, C. L.; Czyz, M. L.; Smith, Z. M.; Hayne, D. J.; Doeven, E. H.; Agugiaro, J.; Wilson, D. J. D.; Adcock, J. L.; Scully, A. D.; et al. The Tandem Photoredox Catalysis Mechanism of [Ir(ppy)2(dtbbpy)]+ Enabling Access to Energy Demanding Organic Substrates. *J. Am. Chem. Soc.* **2019**, *141*, 17646-17658.
